# Rapid parallel adaptation despite gene flow in silent crickets

**DOI:** 10.1038/s41467-020-20263-4

**Published:** 2021-01-04

**Authors:** Xiao Zhang, Jack G. Rayner, Mark Blaxter, Nathan W. Bailey

**Affiliations:** 1 School of Biology, University of St Andrews, St Andrews, Fife, KY16 9TH UK; 2 Tree of Life, Wellcome Sanger Institute, Cambridge, CB10 1SA UK

**Keywords:** Evolution, Genetic variation, Gene expression, Sequencing, Entomology

## Abstract

Gene flow is predicted to impede parallel adaptation via de novo mutation, because it can introduce pre-existing adaptive alleles from population to population. We test this using Hawaiian crickets (*Teleogryllus oceanicus*) in which ‘flatwing’ males that lack sound-producing wing structures recently arose and spread under selection from an acoustically-orienting parasitoid. Morphometric and genetic comparisons identify distinct flatwing phenotypes in populations on three islands, localized to different loci. Nevertheless, we detect strong, recent and ongoing gene flow among the populations. Using genome scans and gene expression analysis we find that parallel evolution of flatwing on different islands is associated with shared genomic hotspots of adaptation that contain the gene *doublesex*, but the form of selection differs among islands and corresponds to known flatwing demographics in the wild. We thus show how parallel adaptation can occur on contemporary timescales despite gene flow, indicating that it could be less constrained than previously appreciated.

## Abstract (web summary)

Gene flow is classically thought to impede local adaptation via parallel evolution. However, a genomic study on Hawaiian crickets from different island populations finds evidence of parallel adaptation to the same lethal parasitoid in spite of strong ongoing gene flow.

## Introduction

Adaptive evolution can be surprisingly repeatable. Convergent phenotypic evolution occurs when similar selection pressures drive the spread of functionally identical traits at the organismal level but in different populations^1,2^. One way this can occur is through adaptive introgression, by repeatedly introducing the same pre-existing adaptive allele from one population into others, whereupon they are selected. In this scenario, gene flow is predicted to play an important role in moving the same allelic variants, which share a single mutational origin, among populations^3^. In contrast, parallel evolution occurs when traits that are functionally identical at the organismal level nonetheless have independent mutational origins at the genetic level^3,4^. In the latter case, uniquely arisen adaptive genetic variants are independently selected in different populations, and theory strongly predicts that high levels of gene flow will reduce the likelihood of parallel evolution^5–10^. For that reason, the most compelling examples of parallel evolution are those that have been documented in highly isolated populations, for example, the independent selection of different genetic variations that enable rabbits (*Oryctolagus cuniculus*) on different continents to survive myxoma virus^11^, and the repeated adaptive deletion of the *Pitx1* gene, which causes stickleback (*Gasterosteus aculeatus*) pelvic reduction in different spatially isolated lake systems^12,13^. Exceptions to this general pattern are only predicted when selection is extreme or mutation rates are particularly high^3,14^, but these scenarios are difficult to empirically test in natural systems. Most adaptive novelties arose and spread in the distant past, necessitating the use of sophisticated techniques to distinguish processes such as incomplete lineage sorting and gene flow^15–17^. Dissecting the mutational origins of novel adaptive traits undergoing convergent phenotypic evolution, while simultaneously inferring gene flow, is complicated by these sorts of demographic developments occurring during and after episodes of adaptive evolution^18,19^. Thus a question at the centre of arguments about evolvability and adaptive potential remains largely unsolved: Can parallel evolution underpinned by independent mutations occur despite high levels of gene flow, and if so, how?

**Fig. 1:**
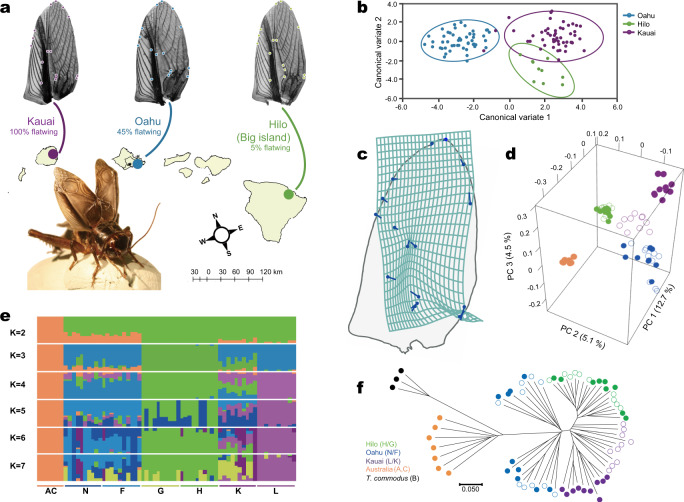
Geographic, morphological and genetic variation in silent Hawaiian crickets. **a** Representative right forewings of flatwing *T. oceanicus* males from populations on the Hawaiian islands of Kauai, Oahu and the Big Island (Hilo). Map drawn using QGIS v2.18.24 (http://qgis.org). Dots on wing veins indicate geometric morphometric landmarks, and colours reflect the scheme used in this manuscript if not stated otherwise (purple = Kauai, blue = Oahu, green = Hilo). Bottom left photo: A normal-wing male for comparison (Photo credit: N.W. Bailey). **b** Flatwing wing venation differs among the three populations. Canonical variates analysis (CVA) showing multivariate differences in wing veins of flatwing males from Kauai, Oahu and Hilo. Source data are provided as a Source Data file. **c** Deformation grid illustrating the major differences in forewing venation between flatwing males from the three populations. The outline indicates a generic male forewing, and arrows show vectors representing landmark variation on CV1 (scaling factor of 10). **d** There is clear population genetic differentiation among the three islands. Genome-wide differentiation among *T. oceanicus* populations shown in a three-dimensional PCA scatter diagram based on autosomal SNPs. Australian *T. oceanicus* outgroup samples are indicated in orange. Solid circles = normal-wing males, open circles = flatwing males. **e** However, population structure analysis indicated an optimal group size of *K* = 5, likely reflecting our inclusion of lab-raised normal-wing Kauai males and lab-raised flatwing Hilo males. Different colours indicate genetically distinct groups. **f** Neighbour-joining phylogenetic tree based on autosomal SNPs. Symbols and colour coding for *T. oceanicus* samples are as above, and black circles = *T. commodus* outgroup. The scale bar represents the level of genetic similarity.

We addressed this question using a unique system of rapidly evolving field crickets (*Teleogryllus oceanicus*) on the Hawaiian archipelago. Typically, male crickets attract females for mating by rubbing their wings together to produce a conspicuous, long-range acoustic signal. However, in approximately 2003, a variant segregating in the manner of a single locus on the X chromosome—*flatwing*—arose and spread in a population on the island of Kauai^20^. This adaptive allele erases sound-producing structures on males’ wings during development, causing them to resemble the undifferentiated wings of females (Fig. 1a)^21^. Silence protects these ‘flatwing’ males from fatal attack by an acoustically orienting endoparasitoid fly, *Ormia ochracea*^20^. The combination of a novel adaptive variant and acoustically orienting parasitoids has driven the recent, rapid evolution of male silence, increasing the abundance of flatwing males from 0% to over 95% in ca. 20–30 generations^20^. Protective flatwing male morphs were found 2 years later on the neighbouring islands of Oahu, and 5 years later on the Big Island (hereafter Hilo), and evidence from the field shows that the flatwing morph is now fixed at 100% in the Kauai population (Fig. 1a)^21–25^. The flatwing male morph has never been found outside of Hawaii. Previous research using lab-reared lines provided evidence that both Kauai and Oahu *flatwing* loci (*fw*^*K*^ and *fw*^*O*^, respectively) show the same mode of inheritance on the X chromosome yet are predominately associated with different restriction site associated DNA (RAD) markers, suggesting they are independently generated alleles^21,23^.

Here, we take advantage of the repeated evolutionary origin and spread of flatwing crickets in multiple Hawaiian island populations to test the expected trade-off between gene flow and rapid parallel adaptation via independent mutational events, which is predicted by standard evolutionary models^5–10^. We first compare morphometric differences among flatwing male crickets on three islands of the Hawaii archipelago, then resequence 70 cricket genomes at high coverage: normal-wing and flatwing males from all Hawaiian populations, plus a non-parasitized, normal-wing Australian population and a sister species outgroup *T. commodus*. Association and selection analyses localise *flatwing* to different loci in each of the three Hawaiian populations but also highlight a putative genomic hotspot of adaptation, which has been subject to distinct processes that have left different signatures of selection in each population. By reconstructing historical population demographic patterns both autosome-wide and for X-linked loci, we find evidence of extensive, recent, and ongoing genome-wide gene flow among all three populations, but no evidence of *flatwing* introgression. We reconstruct the historical demographic population genetics of these populations as well as signatures of selection on adaptation hotspots in each. We interrogate a candidate region identified by the analyses above, containing the gene *doublesex* (*dsx*), which has been widely implicated in sex-specific adaptive traits in other insects, by quantifying gene expression in developing wingbuds of different male morphs on two islands where flatwing crickets exist in appreciable abundance (Kauai and Oahu). Our results reveal that parallel evolution can occur despite gene flow, indicate how this process occurs, and implicate the *doublesex* pathway in the repeated origins of an adaptive, male-feminising trait in wild insects.

## Results

### Morphological analysis indicates unique wing adaptations

Structures formed by specialised veins on the forewings of male crickets normally produce sound when males move their wings back and forth across one another during singing. However, these structures are either absent or severely reduced in flatwing males. Thus feminised, flatwing males are prevented from making sound even though they still move their wings in the motor pattern characteristic of singing, but without producing audible sound (Fig. 1a)^20,23,26^. Flatwing venation differs between Kauai and Oahu males and appears to be under independent genetic control^23^, so to test whether the wing venation of flatwing males is morphologically distinct among all three populations we studied here, we collected right forewings of 114 wild flatwing male crickets over multiple years from populations on the islands of Kauai, Oahu and Hilo (Supplementary Table 1). Landmark-based geometric morphometric analysis^23,27^ revealed that wing venation of flatwing males differed markedly among the three populations (multivariate analyses of variance: all *P* < 0.001, Supplementary Table 2). We then used canonical variate analysis to visualise the main sources of variation among flatwing males from the three islands. All three populations were distinguishable with Hilo flatwings more similar to Kauai flatwings than to Oahu flatwings (Fig. 1b, c), though this must be interpreted cautiously owing to the small number of Hilo flatwing crickets in the wild. However, this pattern is visible to the naked eye (Fig. 1a). Hilo and Kauai flatwing individuals tend to have completely lost their scraper—the thickened ridge of tissue on the wing margin, which engages the other wing during movement—but a vestigial scraper has been retained by most Oahu flatwings (Fig. 1a, c). Despite this variation, all flatwing males are functionally silent^26^. Although all flatwing males had reduced or absent resonators (enlarged wing cells that vibrate during singing), the degree of this reduction varied among populations (Fig. 1a).

### Background genetic structure of cricket populations under convergent fly selection

We resequenced whole genomes of 70 male crickets to an average depth of 25×. Twenty males (10 of each morph; flatwing and normal-wing) were derived from parasitized populations on each of three Hawaiian islands (Kauai, Oahu and Hilo) (Fig. 1a). Seven normal-wing male *T. oceanicus* from Australia plus three males of the sister species *T. commodus* (which also have normal-wing morphology) were also sequenced. Supplementary Table 3 provides further sampling details. Australian populations are not known to be subject to selection pressure from the parasitoid fly (Fig. 1a), making them a useful comparison in selection analyses, and the sibling species *T. commodus* was used as an outgroup (Fig. 1 and Supplementary Table 3, Supplementary Data 1). All crickets were wild-caught with the following exceptions: Because normal-wing crickets now appear to be extinct in the Kauai population^24,28^, and flatwing crickets only exist in extremely low abundance in the Hilo population^23^, we resequenced lab-reared individuals established from wild-caught ancestors for these groups.

More than 96% of the re-sequencing reads successfully mapped to the *T. oceanicus* reference genome^21^, confirming the quality of this re-sequencing dataset. We used a customised pipeline to account for potential biases caused by outgroups or sequencing errors to obtain a total of 107 million high-quality single nucleotide polymorphisms (SNPs) (Supplementary Data 2). To examine background genome-wide population structure excluding any sex-linked or flatwing-associated effects, a neighbour-joining tree based on pairwise genetic distances was constructed using autosomal SNP data (Fig. 1f). In Hawaii, individuals grouped into three population-specific clades, and Oahu was most closely related to the Australian outgroup (Fig. 1f and Supplementary Fig. 2). Flatwing and normal-wing individuals were not split into two subclades except for Kauai individuals (purple circles of Fig. 1d, f and Supplementary Figs. 2, 3), which likely reflects founder effects among these lab-reared samples. Principal component analysis (PCA) and genetic structure analysis are consistent with the NJ tree (Fig. 1d, e and Supplementary Fig. 1). Morphometric and autosomal genetic data yielded notably similar patterns of relatedness among island populations: Kauai and Hilo were more similar to each other than either was to Oahu despite their greater geographic distance. These morphometric and genomic patterns implied two possible mechanisms for the repeated, rapid, adaptive evolution of flatwing crickets across the Hawaiian archipelago. Under the first scenario, this similarity could be due to adaptive introgression of one causative *flatwing* variant from island to island. Alternatively, the phenotypic expression of different independent *flatwing* variants could be influenced by different genomic background effects, with the similarity of flatwings in Kauai and Hilo caused by their more similar genetic backgrounds.

### Recent, extensive and ongoing gene flow among Hawaiian cricket populations

Migration could have facilitated the adaptive introgression of *flatwing* variants from population to population, whereupon they spread under selection from the fly^29^. In the wild, populations on different islands are assumed to be isolated from one other because of the distinct geographical barrier of the sea; *T. oceanicus* from Hawaii are not known to be capable of sustained flight such as would be required for long-distance dispersal and would have great difficulty crossing many miles of open ocean. However, in Hawaii, human activities including the transport of agricultural goods, tourists and residents make these barriers surmountable. To evaluate gene flow, we assessed the presence and extent of admixture among the three island populations and reconstructed their demographic history. Only wild individuals were used in these analyses.

**Fig. 2:**
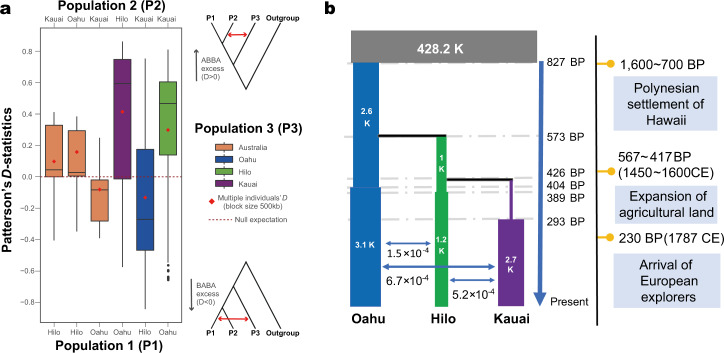
Extensive gene flow among three Hawaiian island populations of *T. oceanicus*. **a** ABBA–BABA tests illustrate significant gene flow among all three Hawaiian populations on the X chromosome. Sites used in the test are derived in *T. oceanicus* compared to the outgroup (*T. commodus*) but differ among P1 and P2 *T. oceanicus* individuals. Tests with Australian *T. oceanicus* populations are included as control comparisons (left three bars) and the plot shows all possible topological permutations of the three Hawaiian island populations relevant for the ABBA–BABA test (right three bars). The null expectation of no gene flow is indicated by *D* = 0. Positive *D* indicates an excess of shared derived alleles—evidence for greater gene flow—between populations 2 and 3 in the comparison (ABBA excess), whereas negative *D* indicates evidence for greater gene flow between populations 1 and 3 in the comparison (BABA excess). The significance of gene flow among island populations was also supported by all comparisons between all Hawaiian scenarios and Australian scenarios (two-sided *t*-tests: all *P* < 0.001; *n* = 700 independent *D*-values for each Australian control; *n* = 1000 each for Kauai and Hilo; *n* = 2000 for Oahu. Statistical details are provided in Supplementary Table 4). Box plots show upper quartile, median, and lower quartile values, 1.5× interquartile ranges, and outliers. Multiple individuals’ *D* tests using a smaller block size are represented by red diamonds. Source data are provided as a Source Data file. **b** The most likely demographic history of Hawaiian *T. oceanicus* populations reconstructed using Fastimcoal2 indicates recent, extensive gene flow among all islands. The ancestral population is shown in grey. Bar width for each population indicates estimated effective population sizes (numbers indicated in each block) and black horizontal lines represent population divergence. Blue horizontal arrows and the numbers underneath them indicate per-generation migration rates, i.e. the estimated rate of symmetrically exchanged migrants between populations every generation; these episodes of gene flow were inferred to have started approximately within the past 300–400 years and are ongoing. This reconstruction provided evidence that all three populations experienced ancient bottlenecks, plus recent population expansions accompanied by gene flow. An approximate timeframe indicating initial human colonisation of the Hawaiian archipelago, agricultural activity, and European contact is provided on the right of the panel. The population demographic reconstruction for *T. oceanicus* indicates that the crickets are likely to have accompanied the first human arrivals to Hawaii.

We used two approaches to evaluate gene flow. First, we implemented Patterson’s *D* statistics (ABBA–BABA test)^30^ using X chromosome data because we were specifically interested in understanding gene flow that could affect the movement of *flatwing* alleles among populations. This analysis revealed considerable gene flow among the three Hawaiian island populations (right three bars of Fig. 2a), which was considerably and significantly higher than that involving Australian populations (orange bars of Fig. 2a and Supplementary Table 4, Supplementary Data 3). All comparisons yielded *D*-values which deviated significantly from the null hypothesis (*D* = 0), which is consistent with common observations that modest but significant gene flow can be detected among geographically distinct distant populations within the same species^31–33^. The key observation is that all Hawaii island scenarios showed significantly more extreme *D*-values compared to gene flow involving Australian populations (two-sided *t*-tests: all *P* < 0.001; Supplementary Table 4). In addition, multiple individuals’ *D*^34^ test using a much smaller block size showed similar results (Fig. 2a and Supplementary Table 5). Although it would be problematic to directly compare *D*-values of the X chromosome and autosomes due to differences in effective population size, we nevertheless note that the X (LG1) showed the strongest signals of gene flow, and the large majority of autosomal linkage groups (LGs) showed qualitative patterns consistent with the X (Supplementary Fig. 4).

Second, to test more detailed scenarios of gene flow and its timing we used the joint site frequency spectrum (SFS) of autosomal SNPs implemented in Fastsimcoal2^35^. The latter analysis is more sensitive but relies on several assumptions that preclude using X-linked markers, so we evaluated 62 models using the SFS of autosomal SNPs to reconstruct the recent demographic history of Hawaiian crickets, incorporating divergence time, changes in effective population size, and gene flow (Supplementary Fig. 5). The models included seven divergence patterns possible for three extant populations plus one assumed ancestral population, all possible scenarios of island permutation for each divergence pattern, and two gene flow scenarios (i.e. whether or not recent gene flow occurred among the three islands) for each permutation.

The results indicate that gene flow among all three islands started recently, and occurred continuously until the present, rather than transiently. Almost all scenarios assuming recent gene flow were more probable than those without gene flow (Supplementary Data 4). The best-fit model suggested that crickets appeared in Oahu first, more than 800 years ago. Then, the Hilo population split from the Oahu population about 570 years ago. Finally, about 400 years ago, the Kauai population diverged from the Hilo population. All three populations experienced ancient bottlenecks followed by recent population expansions, with symmetrical gene flow starting within the last 300–400 years and continuing today (Fig. 2b and Supplementary Data 4). These dates coincide with the history of the Polynesian settlement of Hawaii, followed by expansion of the human population, and the arrival of European explorers, respectively (Fig. 2b). We also used Pairwise Sequentially Markovian Coalescent (PSMC) models to infer more ancient demographic history (Supplementary Fig. 6)^36^. The ancestral effective population size of Hawaiian crickets showed a peak about 20,000 years ago followed by a decline involving at least a twofold decrease in the effective population size coinciding with the last glacial maximum^37^, likely reflecting their putative ‘out-of-Australia’ origins and island-hopping across the Pacific^38,39^.

### Genomic hotspots implicated in rapid parallel adaptation of silent crickets

**Fig. 3:**
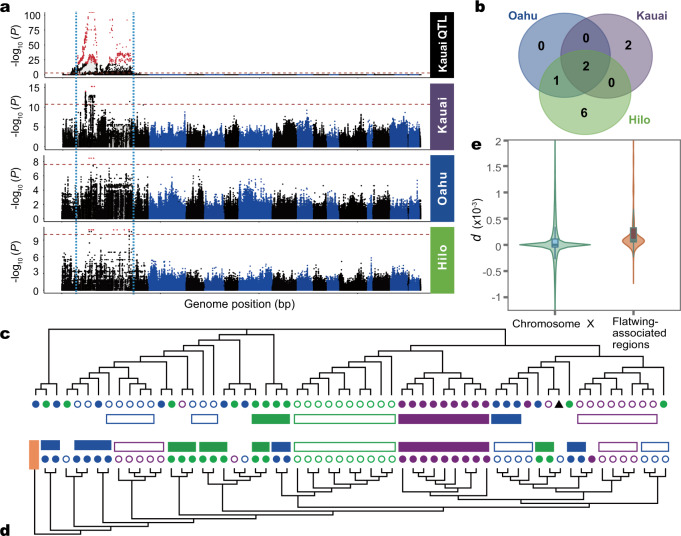
Putative genomic hotspots containing different flatwing-associated loci in cricket populations on three Hawaiian islands. **a** Flatwing association mapping. Data from a previously published flatwing QTL^21^ are shown on top, with red symbols showing flatwing-associated SNPs and blue vertical lines the extent of the QTL on the X-linkage group (left-most block of black markers). Horizontal dashed lines indicate FDR-corrected significance thresholds, and alternating shades of black and blue indicate different linkage groups. For the lower three panels, the most significant markers are defined as described in the main text using the strictest criterion and plotted in red. **b** Venn diagram showing candidate X chromosome scaffolds associated with the flatwing phenotype for each population, all detected using the most stringent Oahu criterion. **c** Unrooted maximum-likelihood consensus tree for the two flatwing-associated scaffolds shared by all islands. **d** Rooted maximum-likelihood consensus tree for all 1 kb flanking regions of all flatwing-associated SNPs. **c**, **d** Normal-wing males are represented with solid circles and flatwing males with open circles. Clustered individuals (≥2) are highlighted using filled (normal-wing) and open (flatwing) boxes if they were from the same island population and shared the same wing phenotype. Black triangles represent the individual used as the reference genome^21^. Orange rectangle represents seven Australian individuals, which were used as an outgroup. **e** Violin plot of net genetic distance (*d*) outside and within flatwing-associated regions on chromosome X between flatwing Kauai and flatwing Oahu samples. Distances were calculated in 10 kb sliding windows with 2.5 kb steps (*n* = 66,211 and 437 windows, respectively). Values for upper quartile, median, and lower quartile, plus 1.5× interquartile ranges, are indicated with box plots in the middle of the violin plots. Additional statistical details are provided in Supplementary Fig. 26a.

To dissect the genetic basis of convergent phenotypic evolution in silent Hawaiian crickets and test whether it has been driven by adaptive introgression or parallel evolution, we performed three independent genome-wide association studies (GWAS), one for each island. Because of the extreme scarcity of Kauai normal-wing males and Hilo flatwing males, GWAS involving these two morphs used lab stocks established from the offspring of wild-caught individuals (see ‘Methods’ section). Our previous quantitative trait locus (QTL) mapping of flatwing in Kauai yielded a broad peak occupying approximately one-third of the X chromosome (redrawn from ref. ^21^ using pseudomolecule coordinates (bp); top panel of Fig. 3a). Here, we substantially narrowed this candidate region to three small SNP datasets varying from 5 to 615 flatwing-associated SNPs, spanning 3–17 scaffolds of the X chromosome (Fig. 3a and Supplementary Table 6, Supplementary Data 5). Among all flatwing-associated SNPs, 83 overlapped with 32 protein-coding genes, most of which have a role in *D. melanogaster* wing development or other relevant developmental processes (Supplementary Data 6).

It was important that inferences from separate GWAS analyses were comparable across Hawaiian populations containing flatwing males; that is, flatwing-associated SNPs needed to be detected with a uniform criterion for false discovery rate (FDR) in all three analyses. We therefore subsetted the Hilo and Kauai flatwing-associated SNPs according to the most conservative criterion based on the Oahu GWAS (Fig. 3a, red dots), for which all samples from both morphs had been obtained from the wild. Both the initial, relaxed set of flatwing-associated markers and the second, conservative set of flatwing-associated markers yielded the same pattern: no flatwing-associated SNP was shared by any of the three populations (Supplementary Table 6, Supplementary Data 5). This pattern was consistent with different genetic causes of flatwing in Kauai, Oahu and Hilo, however, we found that different flatwing-associated SNPs in the populations were sometimes located on the same scaffold. Two of these candidate flatwing-associated scaffolds were shared by all three islands (Fig. 3b and Supplementary Fig. 7, Supplementary Table 7). Considering that one scaffold is a physically continuous genetic region, one possible explanation is that these regions contain potential ‘genomic hotspots’ of adaptation in which recurrent mutations lead to the evolution of flawing morphology. Alternatively, this pattern could be caused by adaptive introgression of the same causative *flatwing* mutation(s) being masked in our analysis by potential biases of performing three separate GWASs, for example, due to population genetic structure. To further evaluate the idea that parallel evolution occurred in genetic hotspots, we established maximum-likelihood phylogenetic trees using SNPs extracted from (1) flatwing-associated scaffolds shared by different populations, (2) GWAS candidate SNP sets from all populations combined, (3) each of the three population-specific GWAS candidate SNP sets separately, and (4) flanking regions of these flatwing-associated SNPs. Given our precise knowledge of how recently flatwing appeared in each population^20,22,23^, if a single *flatwing* allele had arisen and introgressed among populations, then trees built using restrictive sets of markers strongly linked to the flatwing phenotype should show elevated relatedness of all flatwing individuals regardless of their population origin compared to normal-wing individuals. None of the unrooted trees using Hawaiian individuals, nor rooted trees with Australian individuals as outgroups, supported such an adaptive introgression scenario: flatwing individuals from different populations did not all cluster together as would be expected if flatwing-associated regions were shared due to recent introgression across populations (Fig. 3c, d and Supplementary Figs. 8–25). Because the sex of crickets is determined by an XX/XO (female/male) system, and only males were used here (except the one reference genome that was represented by triangle), these trees can also be considered to suggest relationships of different haplotypes.

We further tested this idea using samples for which wild-caught flatwing individuals were resequenced, i.e. Kauai and Oahu flatwings, to avoid confounds of using lab-bottlenecked individuals. If *flatwing* introgressed very recently through these Hawaiian populations, a divergence of flatwing-associated allele(s) between population pairs should be lower than the divergence of other loci on the X chromosome^40–42^. Instead, we found that net nucleotide divergence (*d*) between Oahu and Kauai wild flatwing individuals at flatwing-associated regions was significantly greater than that for all other X chromosome regions (Fig. 3e, two-sided *t*-test: *P* < 0.001; *n* = 437 sliding windows within flatwing region, *n* = 66,211 sliding windows outside flatwing region; additional statistical details are reported in Supplementary Fig. 26a), while absolute divergence (*d*_xy_) between flatwing-associated regions versus other X chromosome regions was similar (Supplementary Fig. 26b, see ‘Methods’ section). These results, combined with a lack of shared flatwing-associated SNPs and structural variants and long-range, complete linkage disequilibrium on flatwing-associated scaffolds (see below) supported the recent independent origins of flatwing crickets on different islands.

Structural variants (SVs) can have a strong influence on adaptive evolution in insects^43,44^. We identified a total of 6971 deletions, 4482 translocations, 3385 inversions, 211 insertions and 17,670 copy number variation regions (CNVRs) located in the X chromosome among Kauai, Oahu and Hilo populations (Supplementary Fig. 27, Supplementary Table 8). If the same structural variants were associated with flatwing morphology on different islands, this would be inconsistent with independent mutational origins of flatwing in those populations. However, we did not uncover any candidate association between structural variants and the flatwing phenotype within islands except for Kauai (Supplementary Fig. 27 and Supplementary Data 7-8), consistent with the hypothesis of parallel evolution.

### Signatures of selection on flatwing-associated regions differ across populations

**Fig. 4:**
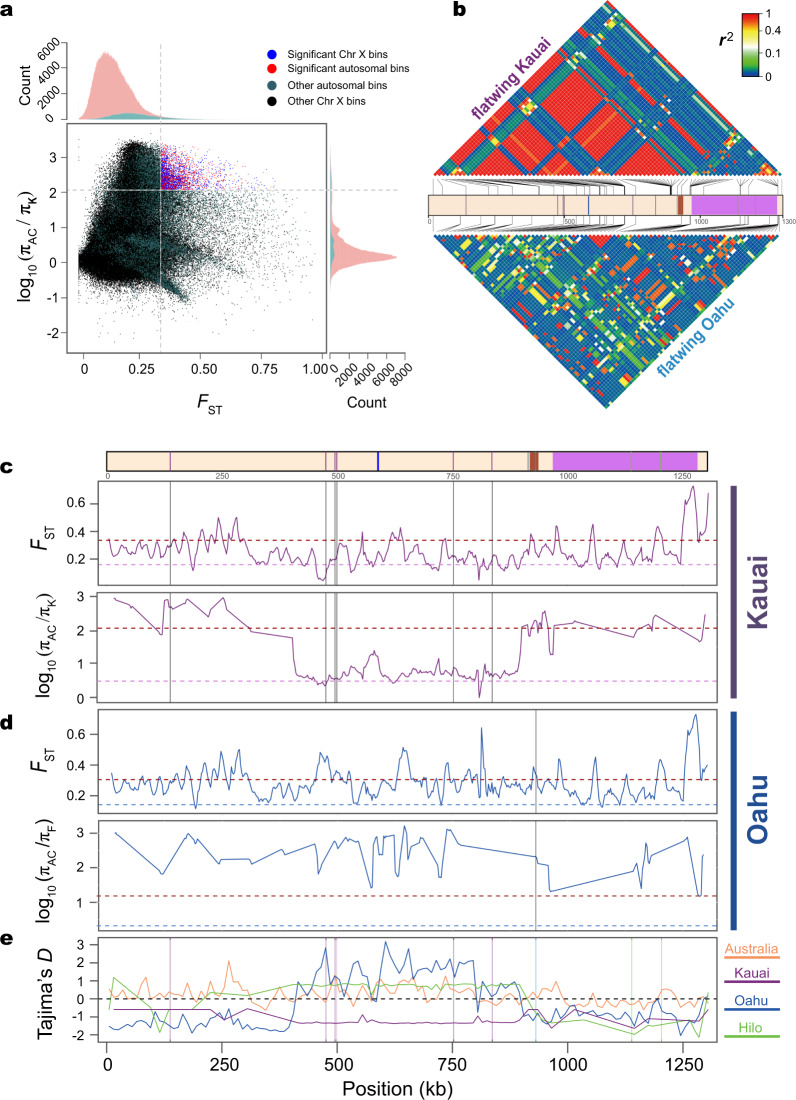
Parallel adaptation is associated with different signatures of selection on putative flatwing hotspot, scaffold 18404. **a** Loci showing signatures of selective sweeps in wild Kauai flatwing males were identified by examining the distribution of fixation index (*F*_ST_) and relative diversity (log_10_[*θ*_*π*,unselected_normal-wing_/*θ*_*π*,selected_flatwing_]) between flatwing Kauai individuals and normal-wing Australian individuals. Datapoints represent 10 kb sliding windows calculated in 2.5 kb increments across the entire genome. Blue points and red points correspond to X chromosome and autosomal bins, respectively, showing the most significant selective-sweep signatures (top 5% of the empirical distribution). **b** Patterns of linkage disequilibrium (LD) along the flatwing-associated scaffold 18404. Top and bottom heatmaps show pairwise LD (*r*^2^) between SNPs (top—Kauai, bottom—Oahu), and SNPs were filtered as detailed in the ‘Method’ section to ensure LD is comparable between flatwing males from Kauai vs. Oahu. The colour scale refers to the value of *r*^2^. Protein-coding genes and flatwing-associated SNPs are indicated by blocks and bars with different colours respectively (*Prospero* = brown, *Doublesex* = purple) in the middle panel. **c**–**e**, Signatures of selection on scaffold 18404 associated with parallel evolution in Hawaiian populations. Genes and flatwing-associated SNPs are shown in the top panel using the same scheme as in **b**. *F*_ST_ and *π* were calculated using sliding-window analyses with 10 kb windows for **c** Kauai and **d** Oahu. *π*_ratio_ = *θ*_π,unselected_normal-wing_/*θ*_π,selected_flatwing_. Horizontal dashed lines represent mean whole-genome value for the values shown in each panel, and dark red horizontal dashed lines in **c** and **d** represent the top 5% threshold values. **e** Tajima’s *D* for all wild-caught individuals. Kauai flatwing (purple), Oahu (blue, all samples combined), Hilo normal-wing (green), Australian normal-wing (orange). The dashed horizontal line indicates the Tajima’s *D* null hypothesis for neutrality.

To examine the genomic signatures of selection on flatwing males, we identified selective-sweep regions by comparing π log-ratios vs. population differentiation (*F*_ST_) between wild flatwing Hawaiian crickets and normal-wing Australian crickets, which are not under selection pressure from *O. ochracea* (Fig. 4a)^33,45^. Approximately 1460 putative selective-sweep regions were identified, comprising ca. 1% of the genome. Fifteen flatwing-associated SNPs were located in the selective-sweep regions (Supplementary Table 9). The two flatwing ‘hotspot’ scaffolds identified by GWAS analyses and shared by all islands (Contig18404_pilon and Contig6636_pilon, hereafter ‘scaffold 18404’ and ‘scaffold 6636’; Fig. 3b) were also recovered in this independent selective-sweep analysis (Fig. 4 and Supplementary Figs. 28–30).

Selection is expected to reduce genetic diversity and elevate linkage disequilibrium (LD)^33,45^. For all wild populations, flatwing individuals repeatedly showed significantly lower nucleotide diversity (*π*) than normal-wing individuals in the flatwing hotspot scaffolds (Wilcoxon rank-sum tests, all *P* < 0.001, 5% of empirical distribution, Fig. 4c, d and Supplementary Fig. 30a, b). *F*_ST_ between flatwing and normal-wing individuals was also significantly elevated above genome-wide levels (Fig. 4c, d and Supplementary Fig. 30). Consistent with recent extreme positive selection, we observed unusually long-range complete LD and significantly negative Tajima’s *D*-values in Kauai, in which rapid evolution completely fixed the flatwing morphotype within ca. 15 years^22,24,25^ (top panels of Fig. 4b and Supplementary Figs. 28, 29). However, the Oahu population showed a much different signature of selection in this region (bottom panel of Fig. 4b, e). For Oahu flatwing individuals, the flanking regions of flatwing-associated SNPs showed only a very modest elevation of LD (Supplementary Fig 28). In addition, when we analysed all the wild Oahu samples (including flatwing and normal-wing), Tajima’s *D* was significantly positive at this strongly selected region (Fig. 4e). This pattern of Tajima’s *D* is consistent with balancing selection, which in turn is consistent with field observations that the population in Oahu persistently contains around 45% flatwing males^22^. The discovery of a new morph of silent male, which is known to protect against the fly, ‘curly-wing’, in Oahu raises the intriguing possibility that balancing selection arises as a result of a competing, adaptive male-silencing variant, which exerts its phenotypic effects not through disrupting male wing venation, as in flatwing, but instead through a disruption of the 3-dimensional structure of the wings^25^. Finally, in contrast with Kauai and Oahu populations, Tajima’s *D-*values of Australian and Hilo populations fluctuate around 0 in this region (Fig. 4e).

### Flatwing gene expression during wing development differs across islands and implicates *doublesex*

We performed a separate gene expression study to examine the developmental genetic basis of the flatwing phenotype and test whether this is linked to the putative hotspot identified above, scaffold 18404. Our GWAS and selection analyses highlighted two important candidate genes, *prospero* (*pros*) and *doublesex* (*dsx*), and three flatwing-associated SNPs supported in the selection analysis were directly located within introns of these genes (Fig. 4c, top panel). *Pros* and *dsx* both have known roles in insect wing development. *Pros* is a transcription factor targeting *Tollo*, which induces cell sorting in *Drosophila* wings^46,47^. *Dsx* is also a transcription factor widely involved in sex-specific developmental patterning^48,49^, with intriguing relevance to the evolution of sex-biased wing venation in flatwing crickets. For example, a wing-specific isoform of *dsx* acts as a master regulatory switch in the butterfly *Papilio polytes*, switching an entire polymorphic, mimetic wing pattern on and off in females^50^.

**Fig. 5:**
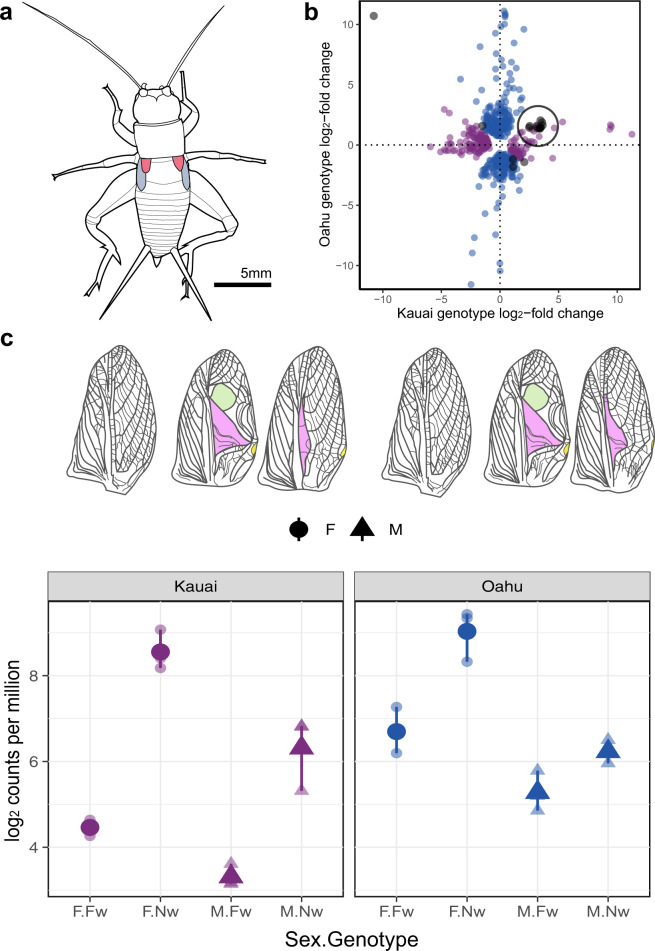
Differential gene expression analysis of developing male forewings. **a** Diagram of the penultimate nymphal instar from which right dorsal forewing buds were sampled. Forewings are highlighted in red, hindwings in blue. Diagram drawn by JG Rayner. **b** The majority of transcripts identified as differentially expressed between *normal-wing* and *flatwing* genotypes in Kauai (purple) and Oahu (blue) were non-overlapping, with the exception of 15 transcripts identified in black. Ten of the latter showed coordinated changes in expression (highlighted with a circle). Log_2_-fold changes > 0 on either axis indicate normal-wing biased expression. **c** Top panel: diagrams of forewing phenotypes for female, flatwing and normal-wing males from Kauai and Oahu^23^. Flatwing males’ wings are feminised to different degrees in Kauai vs. Oahu, but female forewings do not noticeably differ between genotypes. Bottom panel: corresponding patterns of relative expression of *doublesex* in females and males from each population. Large points show means, small points show individual observations for each replicate, and lines indicate the minimum and maximum values. Source data are provided as a Source Data file. Diagram is drawn by JG Rayner.

Crickets are hemimetabolous and wing venation is established early during nymphal development^51^, and there are widespread gene expression differences between *flatwing* and *normal-wing* genotypes during embryonic development even prior to hatching^21^. Crickets develop wingbuds from thoracic precursors, which eventually become externally articulated during later nymphal stages^52^. As such, we performed gene-level differential expression analyses of developing wingbuds at the earliest stage when they become freely articulated, two instars prior to adult eclosion (Fig. 5a). We collected wingbud samples from pure-breeding *normal-wing* and *flatwing* lines that we previously established from Kauai and Oahu populations (no such lines were available for Hilo), and expression analysis reinforced the importance of the genomic region containing *dsx* to the manifestation of independently derived flatwing phenotypes from different populations.

There was little overall similarity in wingbud gene expression changes associated with *flatwing* genotypes from Kauai vs. Oahu (Fig. 5b). In Kauai, 190 transcripts were uniquely DE between morphs, and in Oahu 449 transcripts were uniquely DE between morphs. Just 10 transcripts showed correlated differential expression in both populations, and all of them were downregulated in *flatwing* genotypes. Two of these consistently DE transcripts were located on scaffold 18404. One was *dsx*, the other a transcript with no functional annotation from an overlapping region of the scaffold; both were among a cluster of transcripts that showed correlated morph-associated expression differences between islands (Supplementary Fig. 31). The extent of *dsx* downregulation in *flatwing* genotypes mirrored the completeness with which song-producing structures on male wings were feminised in each population; that is, *dsx* was more downregulated in Kauai flatwing males, where male-specific wing venation is more reduced (Fig. 5c). *Dsx* showed higher overall expression in females, but was also sensitive to genotype with lower expression in *flatwing* carriers. These sex-specific effects of *dsx* are consistent with sex-specific developmental effects that have been found in other organisms, such as horned beetles (*Onthophagus taurus*) in which *dsx* dysregulation affects the development of male and female horn length in opposing directions^48,49^. We found no evidence of sex- or morph-segregating *dsx* isoforms. We cannot exclude that these might be detected at a different developmental stage or in different tissues, but note with interest that there are now multiple exceptions to the expectation of sex-specific splicing in arthropods^53^. The expression patterns of the above two transcripts were further validated by re-analysing an independent, previously collected RNA-seq dataset (see ‘Methods’ section). *Prospero* was lowly expressed in wingbud tissues (mean count-per-million of 0.35) so did not pass expression filtering of transcripts, nor showed any evidence of differential expression between genotypes after relaxing the filtering threshold (unadjusted *P* > 0.88) (Supplementary Fig. 33). These results implicate a well-known regulator of sex-specific phenotypic development in the rapidly evolved loss of male song in *T. oceanicus*, and provide further evidence that a genomic adaptation hotspot has a vital role in determining multiple independently derived male-silencing phenotypes. Although it is currently not possible to establish whether *dsx* expression is causative or an associated downstream effect of flatwing development, these findings suggest a tentative genetic mechanism of flatwing determination. Mutations in this region may alter the expression level of *dsx*, and contribute to spatio-temporal variation of downstream genes.

## Discussion

Understanding the genetic mechanisms of adaptive evolution is a major focus of evolutionary biology^54^. Dissecting the mechanisms underlying convergent phenotypic evolution is extremely useful for gaining a better understanding of adaptation more generally: why, in some cases, do adaptations in different populations and species share a genetic origin, whereas in other cases functionally equivalent adaptations are the result of multiple independent evolutionary origins? Gene flow is generally thought to counteract the generation and maintenance of such parallel adaptations because it can introduce pre-existing adaptive alleles into populations under selection pressure, which can then be directly selected faster than de novo adaptive mutations can arise^3–10^. However, our results from silent Hawaiian crickets surprisingly show that this need not be the case.

The recent, abrupt, and rapid evolution of flatwing male crickets in multiple Hawaiian populations is one of the fastest rates of convergent phenotypic evolution documented in the wild^21,23^. We found readily detectable variation in the morphology of adaptive flatwing phenotypes from populations on Kauai, Oahu and Hilo, and these morphotypes were associated with different genetic loci. There were multiple signals of independent origins of the silent flatwing male phenotype in different island populations: (1) distinct morphometric patterns of flatwing venation on different islands, (2) entirely non-overlapping flatwing-associated SNPs among all three populations, (3) all phylogenetic trees for flatwing-associated genetic regions falsified a scenario of adaptive introgression (4) greater net nucleotide divergence at flatwing-associated regions (5) lack of shared flatwing-associated structural variants (6) long-range, complete linkage disequilibrium on flatwing-associated scaffolds in Kauai but not Oahu (7) little overall similarity in wingbud gene expression changes associated with flatwing genotypes from different islands. This parallel adaptation occurred despite considerable recent, ongoing gene flow among all three populations. *T. oceanicus* in the Hawaiian islands have, like many island species, lost long-distance flight capability, and migration appears to be maintained by human activities, which became more frequent as human settlement increased 300 years ago (Fig. 2b).

Flatwing phenotypes from different islands were associated with a small number of shared putative adaptation hotspots in the genome, and signatures of selection on these varied from population to population and corresponded to known demographic dynamics in the wild. Follow-up gene expression analyses gave further support for the involvement of a transcription factor well-known for controlling sexually dimorphic morphological traits, *doublesex*, in multiple origins of adaptive male wing feminisation. These results illustrate how strong selection associated with genomic hotspots of adaptation can overwhelm the generally predictable influence of gene flow. It remains an open question what level of gene flow would inhibit the parallel evolution of male-silencing variants in this system. However, it is notable that the gene flow we detected in *T. oceanicus* was particularly strong compared to estimates in other species^55–57^. The precise dynamics of mutation, migration and drift in shaping adaptation is a longstanding and complex problem, so in the silent flatwing cricket system and in other systems undergoing rapid convergent evolution, it would be illuminating to perform evolve and resequence experiments to define threshold levels of gene flow beyond which parallel mutations localised within distinct populations become swamped by adaptive variants originating from other populations.

The tension between mutation and migration in shaping the dynamics of adaptive evolution is a central idea in evolutionary theory, which predicts that adaptive introgression should reduce the role of de novo mutations when selection is extreme and gene flow is high^3,5,14^. Exceptions may occur when mutation rates are particularly high, but it has been challenging to evaluate whether these trade-offs shape convergent phenotypic evolution in a predictable fashion. Most well-characterised cases of parallel evolution via independent mutation involve a clear trade-off between mutation and migration. For example, recurrent pelvic reduction in freshwater populations of the stickleback *Gasterosteus aculeatus* occurs due to a hyper-mutable enhancer region of the transcription factor *Pitx1*^12,13^. Affected populations are isolated due to the repeated invasion of separate freshwater systems, yet high mutation rate of an important genomic hotspot of adaptation has facilitated parallel evolution of adaptive pelvic reduction in each habitat. In contrast, introgressive hybridisation between different species of mice introduced a pesticide resistance allele from *Mus spretus* to *M. musculus domesticus*, which was subsequently selected due to the increased application of anticoagulant pesticides in the mid-1900s^58^. Similarly, there is compelling evidence that adaptive loci associated with mimetic wing patterning in *Heliconius* butterflies have introgressed from species to species, facilitating convergent adaptive radiations^16,59^. It is important to note that shared derived adaptations introduced via migration as well as uniquely evolved adaptations can co-occur, and compelling examples have been observed in the context of antimalarial resistance in *Plasmodium falciparum*^60^. However, the evolutionary processes underlying the origins of these adaptations overwhelmingly obey the mutation-migration trade-off. Our findings contradict this general tendency and raise the interesting possibility that incompatibilities between unique, derived adaptive alleles could act as constraints on convergent evolution via introgressive hybridisation. A hypothetical example is where one coevolved system is resistant to introgression of alternative alleles because the latter are individually maladaptive in combination with the resident system. There is precedence for such genomic incompatibilities to affect adaptive variants, for example the homozygous lethal inversion polymorphism in the ruff, *Philomachus pugnax*^61^. In the cricket system, it is known that *flatwing* haplotypes from Kauai (*fw*^*K*^) and from Oahu (*fw*^*O*^) can be expressed in alternative genomic backgrounds^23,27^, but the fitness consequences of this are not known. Future work testing constraints on convergent evolution via adaptive introgression is necessary to evaluate this scenario.

Empirical examples that violate this predictable trade-off are rare. Part of the reason for this may be the well-known challenges of disentangling demographic signal from introgression or independent mutational origins when convergent evolution is supposed to have occurred in the distant evolutionary past^19^, but the silent Hawaiian cricket system is uniquely suited to overcome this issue owing to the recent origin and spread of flatwing morphs in all three populations studied here^22^. If, as our results suggest, parallelism is less constrained by gene flow than previously appreciated, it is worth considering what factors favour such an outcome. The functional genetic basis of adaptive traits and the phenotypic nature of the adaptation may each have important roles in relaxing this constraint. In crickets and other ensiferan insects, forewing venation is a sexually dimorphic trait critical for sexual reproduction. Signals emitted by resonating membranes on the wings are major components of the mate recognition system, often the first long-range signal detected by receivers, and they have a well-known role in sexual selection, reproductive isolation and diversification^62^. Male forewings and the signals they produce are highly variable across ensiferan taxa^27,63–65^. Traits showing an obvious signature of extensive evolutionary variation have, by definition, arisen through the action of repeated genetic mutation, and could therefore be particularly disposed to recurrent parallel evolution. In addition, loss of an existing complex trait may be more evolvable than gain of a novel trait^66^, because disrupting the developmental genetic pathways underlying a complex structure such as a cricket wing resonator or a stickleback’s pelvic girdle can be achieved through simple genetic change affecting one of many loci. Involvement of the *doublesex* pathway in the evolution and development of flatwing male crickets is consistent with such an evolutionary mode of action. *Dsx* is an essential regulator of sexual dimorphism in insects, for example, controlling the development of sexually dimorphic horns in the dung beetle *Onthophagus taurus*^67^ and the Japanese rhinocerous beetle *Trypoylus dichotomus*^68^, female-limited wing mimicry in *Papilio* butterflies^50,69,70^, sexual morphology in *Drosophila melanogaster*^48,49^, and male scent organs in *Bicyclus* butterflies^71^.

The emergence and spread of parallel adaptations in populations subject to gene flow are liable to be biased towards highly evolvable traits such as those functioning in sexual selection and towards genomic hotspots of adaptation where relatively simple changes result in functional losses. Given the increasing research focus on evolutionary rescue and rapid adaptation in wild populations, the speed of such adaptation, its repeatability, and the factors favouring it are important to dissect and translate to applied contexts, to mitigate resistance evolution in agricultural pests and human pathogens, and also to design effective interventions to cope with negative consequences of anthropogenic environmental change^72^. The repeated loss of song in Hawaiian crickets under pressure from eavesdropping parasitoids provides a compelling test case in a wild population undergoing ‘real-time’ evolutionary adaptation, showing that extremely rapid parallel adaptation can occur despite evolutionary forces traditionally thought to impede it.

## Methods

### Morphometric analyses of male wings

Right forewings of wild flatwing male crickets were collected from three Hawaiian islands and mounted between two microscope slides (Supplementary Table 1). Fifty-two Kauai flatwings, 52 Oahu flatwings, and 10 Hilo flatwings were sampled. The unbalanced sample size was caused by the extremely low frequency of flatwing crickets in the wild in Hilo^22^. Slides were photographed using a Leica DFC295 digital camera mounted on a Leica M60 dissecting microscope. We performed a landmark-based geometric morphometric analysis of these photos^27^. Specifically, landmark data were generated by manually placing 16 landmarks on each wing photograph using the tpsDig2 (v2.31)^73^ as described in Fig. 1a. The criteria for placement of each landmark are identical to those used by Pascoal et al.^23^ and Bailey et al.^27^. Morphological comparisons were then conducted using MorphoJ (v1.07a), which extracts shape information by performing a Procrustes superimposition on all the landmark data^74^. A covariance matrix generated from Procrustes distances was then used to perform principal component analysis (PCA) without a priori information of group (i.e. island) identity. The PCA results and corresponding group information were then analysed using a multivariate analysis of variance (MANOVA) in R v. 3.5.2 to test whether flatwings from the three groups were morphologically different. They were, so we used a canonical variates analysis (CVA) to visualise the differences among islands. CVA is designed to maximise the separation of three populations, producing axes, which best explain inter-group variation^23^. A scatter diagram and a transformation grid plot were plotted to visualise the CVA results.

### Sampling, DNA extraction and whole-genome re-sequencing

Seventy individuals were collected for whole-genome re-sequencing (Supplementary Table 3). We used ten flatwing males and ten normal-wing males from each Hawaiian island (Kauai, Oahu and Hilo), seven Australian *T. oceanicus* males and three males of a closely related, but reproductively isolated sister species from Australia, *T. commodus*. The normal-wing males from Hilo and flatwing males from Kauai were collected from the wild in 2017. However, normal-wing males were non-existent in the Kauai population at that time, and flatwing males were extremely scarce in the Hilo population, so for these groups we used laboratory specimens from stock populations derived from previous collections at exactly the same locations; this enabled us to breed extremely large population sizes to obtain enough of the rare morphs for re-sequencing. For Oahu crickets, both flatwing and normal-wing samples were from wild populations. Australian individuals were collected from the wild in Australia in 2011. Single hind legs of each sampled individual were collected and preserved in 100% ethanol. Genomic DNA was extracted from legs using the CTAB method. The quantity of the extracted DNA was measured using a Qubit dsDNA kit. The quality of the DNA samples was evaluated using Nanodrop, 1% agarose gel electrophoresis, AATI Fragment Analyser and the Standard Sensitivity Genomic DNA Analysis Kit. The Hamilton MicroLab STAR liquid handling system was used to pre-normalise DNA samples before library preparation. DNA was sheared to a 450 bp mean insert size using a Covaris LE220 focused-ultrasonicator. Sequencing libraries were prepared using Illumina SeqLab specific TruSeq Nano High Throughput library preparation kit, Hamilton MicroLab STAR and Clarity LIMS X (4.2) Edition, and then clustered onto a HiSeqX Flow cell v2.5 on cBot2s. The clustered flow cell was transferred to a HiSeqX for sequencing using a HiSeqX Ten Reagent kit v2.5.

### Sequence quality checking, read alignment and SNP identification

Demultiplexing was performed using bcl2fastq (v2.20, Illumina), allowing 1 mismatch when assigning reads to barcodes. Adaptors were trimmed during the demultiplexing process. Raw reads that included ≥5% unidentified nucleotides (N) or more than 65% low-quality nucleotides (Phred quality value ≤ 7) were discarded^33^. Before alignment, index files of the field cricket (*T. oceanicus*) reference were generated using bwa and samtools (v1.3.1)^75^. The filtered pair-end sequence reads were then mapped to the reference genome^21^ using BWA-MEM (v0.7.12) with default parameters^76^. Secondary alignments with flag ≥ 255 were discard using a customised Perl script^33^. Samtools (v1.3.1) was used to assess coverage individually. We used samtools and picard (2.14.1) to sort, format and index the resulting binary alignment map (BAM) files and remove PCR duplicates. To increase the accuracy of alignments around indel regions, we detected indels and re-aligned these regions using RealignerTargetCreator and IndelRealigner functions in GATK (v3.7.0)^77^. All following analyses were conducted based on these re-aligned BAM files. We detected SNPs and indels following GATK best practice workflows^78^. Briefly, raw SNPs and indels were first detected individually using HaplotypeCaller. CombineGVCFs was than applied to merge SNPs of different individuals from the same population. The VCF records of multiple populations were then re-genotyped and aggregated using GenotypeGVCFs. Following the authors’ recommendations, hard-filtering criteria (Qual By Depth < 2.0, Fisher Strand > 60.0, RMS Mapping Quality < 40.0, Mapping Quality Rank-Sum Test < −2.5, Read Pos Rank-Sum < −1.0) were decided manually based on density plots of the raw SNP dataset and applied using SelectVariants and VariantFiltration. To further improve the accuracy of genotyping and economise computing resources, SNPs of extremely short scaffolds (≤5 kb) were not called. In consideration of the fact that sex of crickets is determined by an XX/XO (female/male) system^21^, all males were fixed to have homozygous genotypes of X-linked loci^79^. Low-quality SNPs potentially caused by sequencing error were also discarded using a customised Perl script. Specifically, all SPNs with Phred-scaled quality score < 30 or with abnormal depth (lower than one-third or higher than three times the average sequencing depth of a particular individual) were filtered^33^. The X scaffold was defined according to the previously published linkage map^21^. For SNPs located in X-linked scaffolds, the criteria of abnormal depth were set as the half that of the autosomes. Heterozygous calls of X-linked SNPs were fixed to homozygous based on the allele depth if the depth of one allele was greater than three times the other one. If none of these alleles were supported by the supermajority, the genotype was treated as unknown. Loci that had unknown genotypes in more than 25% of all individuals were also excluded. To avoid bias caused by outgroups (unrelated populations), multiple SNP datasets were generated independently and utilised accordingly for the following analyses (Supplementary Data 2).

### Phylogenetic, principal component (PC) and population structure analyses

We used PLINK (v1.90b6.8)^80^ to calculate identity-by-state based on SNP datasets. The resulting distance matrix was then passed to PHYLIP (v3.696)^81^ to construct neighbour-joining phylogenetic trees (Fig. 1f and Supplementary Figs. 2, 3). *T. commodus* individuals were used as an outgroup. We generated consensus trees by performing bootstrap replication 1000 times and merging them. Consensus trees were then labelled and visualised using MEGA (v10.0.5)^82^ and FigTree (v1.4.4). We used the smartpca programme in EIGENSOFT (v7.2.1)^83^ to perform the principal component analysis (PCA) and visualised the result by plotting a scatter diagram using rgl and ggplot2 R packages^84^. We used ADMIXTURE (v1.3.0) to estimate population structure with the numbers of ancestral populations (*K*) from 2 to 7 after thinning the SNP set for linkage disequilibrium using PLINK with an *r*^2^ threshold of 0.1^85^.

### Genetic diversity and linkage disequilibrium analyses

We used VCFtools (v0.1.16)^86^ to calculate genetic diversity with a sliding-window size of 10 kb and a step size of 2.5 kb. To calculate linkage disequilibrium, the original SNP dataset was first divided into subsets of each population using vcftools. The split VCF files were then converted to haploview-recognised format using PLINK. Haploview (v4.2) calculated correlation coefficients (*r*^2^), which are used to infer pairwise linkage disequilibrium^87^. To visually depict LD patterns on scaffold 18404 (Fig. 4b), a reasonable number of SNPs dispersed across the whole scaffold is necessary. However, different sets of positions were polymorphic in flatwing Oahu and flatwing Kauai individuals, which makes direct visual comparison difficult to interpret. To minimise visual bias caused by using different SNP sets, we used the flatwing Oahu group as a standard and then selected the corresponding SNP in the Kauai group; for sites at which the same SNP was unavailable in Kauai, the nearest one in its flanking regions was selected (Supplementary Data 9).

### Gene flow and demographic history inference

To avoid bias caused by inbreeding, only wild-caught, unrelated samples were used to infer gene flow and demographic history of Hawaiian cricket populations. We used the ABBA–BABA (*D*-statistic) test^30,88^ and multiple individuals’ *D*-statistic test^34^ to detect gene flow among different populations. Six different scenarios were statistically tested. To do so, BAM files of individuals from three Hawaiian populations, one Australian population and one outgroup population (*T. commodus*) were passed to ANGSD (v0.930), which counted the number of ABBA and BABA sites and calculated *D*-statistic metrics^34,88^. We inferred relatively late (within 10,000 generations) demographic parameters including the divergence of three populations, bottlenecks and migrations from the site frequency spectrum (SFS) using Fastsimcoal^35^. To minimise bias caused by potential inbreeding, linkage and selection, the following criteria were applied to further filter SNP datasets before format conversion: (i) Only autosomal scaffolds were retained, based on previous linkage map, (ii) SNPs with an *r*^2^ > 0.3 were excluded using the sliding-window method, (iii) PLINK was used to exclude one of the individual pairs, which had a proportion identity-by-descent outlier, (iv) SNPs located inside genic regions were excluded, (v) Sites with unknown genotype were also filtered. The filtered SNP dataset was converted to Arlequin format using PGDSpider (v2.1.1.5)^89^. We used Arlequin (v3.5)^90^ to produce the minor allele SFS (folded SFS) and joint multidimensional SFS. Using Fastsimcoal (v2.6.0.3), we tested 62 models including effective population size changes, different divergence scenarios, presence or absence of gene flow, and permutations of three island populations. For each model, we performed 50 independent parameter inferences. For each run, 100,000 simulations per likelihood and 40 Expectation Conditional Maximisation (ECM) cycles were performed to estimate parameters with maximum-likelihood. We compared different models using the Akaike Information Criterion (AIC), and estimated the 95% confidence interval of the best model using 30 parametric bootstraps^35^. We used the Pairwise Sequentially Markovian Coalescent (PSMC) method to reconstruct the most recent common ancestor (TMRCA) distribution, infer the divergence time from now to the TMRCA, and finally infer the history of effective population size^36^. Since local density of heterozygotes information was used by PSMC, X-linked scaffolds were excluded from this analysis. To further improve the accuracy of this analysis, only wild individuals with the highest sequencing depth (top 2, sequencing coverage > 26×) were used. We used samtools to generate diploid consensus sequences based on individual BAM file. Loci with abnormal depth were masked by N. PSMC (v0.6.5-r67) was used to infer historical effective population size with a mutation rate of 3.5 × 10^−9^ and a generation time of 0.25 year^91,92^.

### Genome-wide association analysis

The flatwing phenotype is known to be inherited as a single locus, X-linked trait. Sex of crickets are determined by XX/XO (female/male) system, and the expression of *flatwing* is male-limited^21^. We used flatwing and normal-wing males to perform three independent, basic case/control association tests for each Hawaiian population sampled. For Oahu, 10 wild flatwing males and 10 wild normal-wing males were used. Because of the rarity of flatwing males in the Hilo population, and normal-wing males in the Kauai population, it was impossible for us to collect enough samples in the wild. Hence, laboratory stocks were used (see above). Thus for Hilo, we used 10 normal-wing wild males and 10 flatwing lab-reared males. For Kauai, because of the inbreeding of lab-reared normal-wing individuals (caused by the absence of wild normal-wing males), the association analysis was performed between 10 wild Kauai flatwing males and 30 normal-wing males from Kauai lab stock, wild Hilo, and wild Oahu populations. The standard case/control threshold for association significance was generated using Fisher’s exact test implemented in PLINK^79,80^. *P-*values were Bonferroni-adjusted in *R* to account for multiple testing. To ensure results were not affected by different numbers of individuals entering GWAS analyses for different islands, we performed another independent GWAS for the Oahu population using the same strategy as for Kauai (10 wild Oahu flatwing vs. all normal-wing). It generated qualitatively identical results (Supplementary Table 10). This analysis was unavailable for Hilo, because in that population the case group (flatwing) was lab-raised. However, phylogenetic tree analyses (see below, and Fig. 3c, d and Supplementary Figs. 8–25) tested all possibilities, including with trees built from a merged set of all GWAS-significant SNPs and their flanking regions, and none were consistent with *flatwing* introgression.

### Structural variant and copy number variation region detection, and comparison between male phenotypes

We detected structural variants (SVs) across the X chromosome. The alignment results of reads mapped to the X chromosome were first extracted from BAM files using customised Perl script and SAMTOOLS^75^. These extracted BAM files were then passed to bam2cfg to generate a per-invocation config file^93^. Based on this config file and the X-chromosomal BAM files, Breakdancer (1.3.6) was used to detect deletion, insertion, inversion, and translocation^93^. The SVs (i) smaller than 100 bp, or (ii) with a quantity score < 30, or (iii) supported by fewer than 2 reads were filtered^94^. For each island, SVs of flatwing vs. normal-wing morphs were compared. Flatwing-differentiated SVs were defined according to the association significance calculated using Fisher’s exact test. We used the read-depth method implemented in CNVnator (v0.3.3), which is a software combined mean-shift approach, multiple-bandwidth partitioning and GC correction, to detect copy number variants (CNVs)^95^. To maximise the accuracy and sensitivity, a bin size of 100 was used. We also filtered the detected CNVs with *p* > 0.05 or q0 > 50% to avoid potential bias caused by inequality of sequencing depth and the reads mapped to multiple genomic regions^95^. Considering the fact that all individuals used in this research were males, which have only one X chromosome, the copy number was normalised to one. Copy number variation regions (CNVRs) were defined as the genomic regions that showed copy number variations in multiple individuals (>3). The overlapped CNVs of different individuals were merged into CNVRs using the method, which defined the minimum and maximum boundaries of the CNVs as the boundaries of the CNVR^96,97^. CNVRs supported by fewer than three individuals were filtered to further reduce the false discovery rate^97^. To detect flatwing-differentiated CNVRs, both *V*_ST_ and student’s *t*-tests were used^97^. Here, *V*_ST_ = (*V*_T_ − *V*_S_)/*V*_T_. *V*_T_ is the total variance in copy number between the two groups. *V*_S_ is the average of the variance within each group, weighted for its sample size^98,99^. A CNVR in the top 5% of all *V*_ST_ values plus a student’s *t*-test *P*-value < 0.05 was considered to be flatwing-differentiated CNVR^97^.

### Parallel evolution analysis

Intersections of flatwing-associated regions of different populations were calculated and visualised using a Venn diagram tool (Bioinformatics & Evolutionary Genomics, Ghent University). The SNPs of flatwing-associated regions were extracted and converted to MEGA-format using Perl scripts. We constructed maximum-likelihood (ML) phylogenetic trees for flatwing-associated SNPs and their flanking regions (Fig. 3c, d and Supplementary Figs. 8–25) using the Tamura–Nei model in MEGA (v10.0.5)^82^. Absolute divergence (*d*_xy_) and net nucleotide divergence (*d*) were used to estimate the amount of DNA divergence (nucleotide substitutions). For the latter, *d* = *d*_xy_ − (*d*_x_ + *d*_y_)/2, where *d*_x_ and *d*_y_ are per-site pairwise nucleotide differences within populations X and Y, respectively, and *d*_xy_ represents pairwise nucleotide differences between populations X and Y^100,101^. Estimates of *d* and *d*_xy_ were calculated using PopGenome (v2.6.1)^102^. The *d* and *d*_xy_ values of different genomic regions in different populations were compared using sliding-window approaches, and statistical differences were tested using 1000 random simulations implemented with custom Perl and R scripts.

### Detection of candidate selective-sweep regions

We used samtools to calculate nucleotide diversities (*π*) and Tajima’s *D* for different populations^75^. Fixation indices (*F*_ST_) between different populations were also calculated. Specifically, VCF files of corresponding populations were analysed using a sliding-window approach with 10 kb windows and a 2.5 kb step size. Windows overstepping boundaries of scaffolds were filtered. Selection pressure reduces genetic diversity of the targeted region and enhances the degree of differentiation between populations under selection versus those not under selection^33,45^. Here, we compared nucleotide diversities of different populations and calculated their *π* log-ratios in Perl and tested the empirical significance of these measures in R using empirical percentiles. Genomic regions within the top 5% of *F*_ST_ values and top 5% of *π* log-ratios were identified as candidate selective-sweep regions^33,45^, and any adjacent (overlapped) candidate regions were merged in to a single candidate region.

### Gene content

The gene content of candidate selective-sweep regions, significant GWAS loci, flatwing-differentiated SVs, and flatwing-differentiated CNVRs was obtained by comparing their coordinates with the official gene set for *T. oceanicus*^21^. Genes overlapping the corresponding candidate region (loci) were defined as candidate genes. Functional annotations of these genes were assigned based on Swissport, TEMBL and the NR database^103^.

### Sampling developing cricket wingbuds for gene expression experiment

We collected and sequenced 24 RNA samples from the wingbuds of *normal-wing* and *flatwing* genotypes of both sexes, from Kauai and Oahu lab populations. There were 3 replicate pure-breeding lines per group. Populations used in the experiment were derived from eggs laid by wild-caught females from populations of Kauai and Oahu in 2014^52^. Laboratory populations were reared in a growth chamber at 25 °C, under a 12-h L:D cycle. Kauai lines had been recently outcrossed by mixing all lines, and pure-breeding lines were reconstituted prior to the current experiment by performing genetic crosses across two generations to select only those families showing patterns of offspring morph ratios indicating they were derived from homozygous *flatwing* or *normal-wing* dams; males and females from these lines were, therefore, pure-breeding for the respective genotype, with males carrying one copy and females two. Kauai and Oahu lines were reared and maintained under identical conditions, in 20 L boxes with cardboard shelter, and food and water available *ad libitum*. Individuals were removed from stock boxes at their penultimate nymphal instar (Fig. 5a), when developing wings are first externalised, ca. 1.5 months after hatching. Wing differences between *normal-wing* and *flatwing* genotype males arise early during their development and become pronounced at this stage^52^. Individuals were briefly anaesthetised using CO_2_, and the dorsal-right forewing bud was removed using micro-dissection scissors and stored in RNAlater. Wingbuds from five individuals were pooled per sample, and samples were frozen at −20 °C after 24 h at 4 °C.

### RNA extraction and sequencing for gene expression experiment

RNA extractions were performed using a Trizol protocol. RNA purity was assessed using a NanoDrop ND-1000 spectrophotometer, and quality assessed using an Agilent 2100 Bioanalyzer. Following library preparation with RiboZero, samples were sequenced on an Illumina HiSeq 4000, generating 2 × 150 bp paired-end reads. Due to logistical constraints, library preparation and sequencing were performed separately for samples from the two populations, but using identical protocols; to minimise the effect this could have on our results, we restricted differential expression analyses to within-population comparisons. CASAVA v1.8.2 (Illumina) was used for base calling and demultiplexing indexed reads. Adaptor sequences were trimmed from fastq files using Cutadapt v1.2.1^104^ and low-quality bases were removed using Sickle v1.200 with a minimum window quality score of 20. Sequences with high similarity to Eukaryotic ribosomal RNAs were removed from the dataset using sortmeRNA^105^.

### Differential expression analysis

Genome alignment, transcriptome assembly and quantification of expression were performed following Pertea et al.^106^. Reads were aligned to the *T. oceanicus* genome using HiSat2 v2.1.0. A genome-guided transcriptome was then assembled from output files using StringTie v1.3.4, and gene expression values quantified for each of the samples. Differential expression analyses were performed at the ‘gene’ level. To retain in our transcriptome only transcripts with strong empirical support and which appear to be protein-coding, we filtered any without open reading frames of >100 amino acids, and those which were not expressed at >1 count per million in at least three samples from each population as well as a previously sequenced Kauai wingbud RNA-seq dataset (see below). The expression filter was relaxed to >0.1 count per million when investigating patterns of *prospero* expression (Supplementary Fig. 33). The resulting merged and filtered transcriptome contained 30,299 unigenes. Gene counts produced by StringTie were prepared for input into edgeR v3.20.9^107^ using the prepDE.py script made available by the authors^106^. In edgeR, counts were normalised by trimmed means of *M*-values (TMM), after which a single negative binomial GLM was fit incorporating all data, using per-transcript normalised expression values as the response variable. Differential expression (DE) analyses were performed using likelihood ratio tests for pairwise comparisons between morph genotypes and sexes. *P*-values were FDR-adjusted using the Benjamini–Hochberg procedure, and transcripts were considered DE between groups if FDR values were < 0.05. Statistical analyses were performed using R v3.4.1. DE transcripts associated with morph genotype in each population were highly correlated between sexes (Supplementary Fig. 31), so males and females with the same genotype were pooled to maximise statistical power: 205 transcripts were identified as DE between genotypes in Kauai, and 464 in Oahu.

### Validation of gene expression experiment

As partial validation of our results, we compared the identity of transcripts DE between morph genotypes from Kauai with those found to be DE between male morphs in previously sequenced samples from male Kauai wingbuds^52^. The latter data were collected using methods considerably different to our own: individuals came from different biological lines, were not anaesthetised prior to sampling, and there were fewer individuals per pooled sample and more samples per group (*N* = 3 individuals per pool; *N* = 6 samples per male morph). Samples were also sequenced on a different platform (Illumina HiSeq 2000). Thus, overlap between datasets in transcripts identified as DE would be indicative of biologically meaningful and robust results. We aligned these samples to the transcriptome and performed DE analysis following the same procedure as above. The results showed that of the 30 transcripts identified as DE between Kauai male morphs in the current dataset, 10 (including both those located on scaffold 18404, one of which is *dsx*) were also significantly DE in the previously collected dataset, all in the same direction, with well-correlated log_2_-fold changes (Spearman’s rho = 0.648, *P* = 0.049), supporting the validity of our results.

### Reporting summary

Further information on research design is available in the Nature Research Reporting Summary linked to this article.

## Supplementary information

Supplementary information

Peer Review

Reporting summary

Description of Additional Supplementary Files

Supplementary Data 1-4

Supplementary Data 5

Supplementary Data 6

Supplementary Data 7-10

## Data Availability

Raw re-sequencing reads that support the findings of this study have been deposited in the European Nucleotide Archive with the accession number PRJEB39125. Raw RNA-seq reads have been deposited in the same archive with the accession number PRJEB40088. Raw wing morphology data are publicly available at ChirpBase.org [http://download.chirpbase.org/]. Previously published RAD-seq^21^ and RNA-seq data^52^ have been deposited in the European Nucleotide Archive under accession numbers PRJEB29921 and PRJNA283744, respectively. Source data are provided with this paper.

## Source data

Source Data

## Code availability

Custom scripts used in this project are archived and publicly available at ChirpBase.org [http://download.chirpbase.org/]

## References

[1] Losos JB (2011). Convergence, adaptation, and constraint. Evolution.

[2] Conte GL, Arnegard ME, Peichel CL, Schluter D (2012). The probability of genetic parallelism and convergence in natural populations. Proc. Biol. Sci..

[3] Stern DL (2013). The genetic causes of convergent evolution. Nat. Rev. Genet..

[4] Lee KM, Coop G (2019). Population genomics perspectives on convergent adaptation. Philos. Trans. R. Soc. Lond. B Biol. Sci..

[5] Ralph PL, Coop G (2015). Convergent evolution during local adaptation to patchy landscapes. PLoS Genet..

[6] Ralph P, Coop G (2010). Parallel adaptation: one or many waves of advance of an advantageous allele?. Genetics.

[7] Feder AF, Pennings PS, Hermisson J, Petrov DA (2019). Evolutionary dynamics in structured populations under strong population genetic forces. G3.

[8] Patwa Z, Wahl LM (2008). The fixation probability of beneficial mutations. J. R. Soc. Interface.

[9] Aeschbacher S, Burger R (2014). The effect of linkage on establishment and survival of locally beneficial mutations. Genetics.

[10] Bradburd GS, Ralph PL (2019). Spatial population genetics: it’s about time. Annu. Rev. Ecol. Evol Syst..

[11] Alves JM (2019). Parallel adaptation of rabbit populations to myxoma virus. Science.

[12] Xie KT (2019). DNA fragility in the parallel evolution of pelvic reduction in stickleback fish. Science.

[13] Chan YF (2010). Adaptive evolution of pelvic reduction in sticklebacks by recurrent deletion of a Pitx1 enhancer. Science.

[14] Olson-Manning CF, Wagner MR, Mitchell-Olds T (2012). Adaptive evolution: evaluating empirical support for theoretical predictions. Nat. Rev. Genet..

[15] Mallet J (2005). Hybridization as an invasion of the genome. Trends Ecol. Evol..

[16] Consortium Heliconius Genome (2012). Butterfly genome reveals promiscuous exchange of mimicry adaptations among species. Nature.

[17] Skov, L. et al. The nature of Neanderthal introgression revealed by 27,566 Icelandic genomes. *Nature* 582, 78–83 (2020). 10.1038/s41586-020-2225-9 32494067

[18] Whiting MF, Bradler S, Maxwell T (2003). Loss and recovery of wings in stick insects. Nature.

[19] Whiting JR, Fraser BA (2020). Contingent convergence: the ability to detect convergent genomic evolution is dependent on population size and migration. G3.

[20] Zuk M, Rotenberry JT, Tinghitella RM (2006). Silent night: adaptive disappearance of a sexual signal in a parasitized population of field crickets. Biol. Lett..

[21] Pascoal, S. et al. Field cricket genome reveals the footprint of recent, abrupt adaptation in the wild. *Evol. Lett*. **4**, 19–33 (2019). 10.1002/evl3.148 PMC7006468 32055408

[22] Zuk M, Bailey NW, Gray B, Rotenberry JT (2018). Sexual signal loss: The link between behaviour and rapid evolutionary dynamics in a field cricket. J. Anim. Ecol..

[23] Pascoal S (2014). Rapid convergent evolution in wild crickets. Curr. Biol..

[24] Tinghitella RM, Broder ED, Gurule-Small GA, Hallagan CJ, Wilson JD (2018). Purring Crickets: the evolution of a novel sexual signal. Am. Nat..

[25] Rayner JG, Aldridge S, Montealegre ZF, Bailey NW (2019). A silent orchestra: convergent song loss in Hawaiian crickets is repeated, morphologically varied, and widespread. Ecology.

[26] Schneider, W. T., Rutz, C., Hedwig, B. & Bailey, N. W. Vestigial singing behaviour persists after the evolutionary loss of song in crickets. *Biol. Lett*. **14**, 20170654 (2018). 10.1098/rsbl.2017.0654 PMC5830660 29445043

[27] Bailey NW, Pascoal S, Montealegre ZF (2019). Testing the role of trait reversal in evolutionary diversification using song loss in wild crickets. Proc. Natl Acad. Sci. USA.

[28] Rayner JG, Pascoal S, Bailey NW (2019). Release from intralocus sexual conflict? Evolved loss of a male sexual trait demasculinizes female gene expression. Proc. Biol. Sci..

[29] Nouhaud P, Blanckaert A, Bank C, Kulmuni J (2020). Understanding admixture: haplodiploidy to the rescue. Trends Ecol. Evol..

[30] Patterson N (2012). Ancient admixture in human history. Genetics.

[31] Anderson CJ, Tay WT, McGaughran A, Gordon K, Walsh TK (2016). Population structure and gene flow in the global pest, *Helicoverpa armigera*. Mol. Ecol..

[32] Li J (2020). Allopatric divergence and hybridization within *Cupressus chengiana* (Cupressaceae), a threatened conifer in the northern Hengduan Mountains of western China. Mol. Ecol..

[33] Qiu Q (2015). Yak whole-genome resequencing reveals domestication signatures and prehistoric population expansions. Nat. Commun..

[34] Soraggi S, Wiuf C, Albrechtsen A (2018). Powerful inference with the D-statistic on low-coverage whole-genome data. G3.

[35] Excoffier L, Dupanloup I, Huerta-Sanchez E, Sousa VC, Foll M (2013). Robust demographic inference from genomic and SNP data. PLoS Genet..

[36] Li H, Durbin R (2011). Inference of human population history from individual whole-genome sequences. Nature.

[37] Timmermann A, Justino F, Jin FF, Krebs U, Goosse H (2004). Surface temperature control in the North and tropical Pacific during the last glacial maximum. Clim. Dynam..

[38] Tinghitella RM, Zuk M, Beveridge M, Simmons LW (2011). Island hopping introduces Polynesian field crickets to novel environments, genetic bottlenecks and rapid evolution. J. Evol. Biol..

[39] Pascoal S (2016). Sexual selection and population divergence I: The influence of socially flexible cuticular hydrocarbon expression in male field crickets (*Teleogryllus oceanicus*). Evolution.

[40] Norris LC (2015). Adaptive introgression in an African malaria mosquito coincident with the increased usage of insecticide-treated bed nets. Proc. Natl Acad. Sci. USA.

[41] Clarkson CS (2014). Adaptive introgression between Anopheles sibling species eliminates a major genomic island but not reproductive isolation. Nat. Commun..

[42] Ai H (2015). Adaptation and possible ancient interspecies introgression in pigs identified by whole-genome sequencing. Nat. Genet..

[43] Zhou J, Lemos B, Dopman EB, Hartl DL (2011). Copy-number variation: the balance between gene dosage and expression in *Drosophila melanogaster*. Genome Biol. Evol..

[44] Massouras A (2012). Genomic variation and its impact on gene expression in *Drosophila melanogaster*. PLoS Genet..

[45] Liu, Z. et al. Genomic mechanisms of physiological and morphological adaptations of limestone Langurs to Karst Habitats. *Mol. Biol. Evol.* 37, 952–968 (2019). 10.1093/molbev/msz301 31846031

[46] Guenin L, Raharijaona M, Houlgatte R, Baba-Aissa F (2010). Expression profiling of prospero in the Drosophila larval chemosensory organ: Between growth and outgrowth. BMC Genomics.

[47] Kim S, Chung S, Yoon J, Choi KW, Yim J (2006). Ectopic expression of Tollo/Toll-8 antagonizes Dpp signaling and induces cell sorting in the Drosophila wing. Genesis.

[48] Rideout EJ, Dornan AJ, Neville MC, Eadie S, Goodwin SF (2010). Control of sexual differentiation and behavior by the doublesex gene in *Drosophila melanogaster*. Nat. Neurosci..

[49] Kijimoto T, Moczek AP, Andrews J (2012). Diversification of doublesex function underlies morph-, sex-, and species-specific development of beetle horns. Proc. Natl Acad. Sci. USA.

[50] Kunte K (2014). doublesex is a mimicry supergene. Nature.

[51] Snodgrass, R. E. *Principles of Insect Morphology* (Cornell University Press, 1993).

[52] Pascoal S (2016). Rapid evolution and gene expression: a rapidly evolving Mendelian trait that silences field crickets has widespread effects on mRNA and protein expression. J. Evol. Biol..

[53] Price DC, Egizi A, Fonseca DM (2015). The ubiquity and ancestry of insect doublesex. Sci. Rep..

[54] Charlesworth, D., Barton, N. H. & Charlesworth, B. The sources of adaptive variation. *Proc. Biol. Sci*. **284,** 20162864 (2017). 10.1098/rspb.2016.2864 PMC5454256 28566483

[55] Corl A (2018). The genetic basis of adaptation following plastic changes in coloration in a novel environment. Curr. Biol..

[56] Sprengelmeyer QD (2020). Recurrent collection of *Drosophila melanogaster* from wild African environments and genomic insights into species history. Mol. Biol. Evol..

[57] Lujan NK, Weir JT, Noonan BP, Lovejoy NR, Mandrak NE (2020). Is Niagara Falls a barrier to gene flow in riverine fishes? A test using genome-wide SNP data from seven native species. Mol. Ecol..

[58] Song Y (2011). Adaptive introgression of anticoagulant rodent poison resistance by hybridization between old world mice. Curr. Biol..

[59] Edelman NB (2019). Genomic architecture and introgression shape a butterfly radiation. Science.

[60] Pearce RJ (2009). Multiple origins and regional dispersal of resistant dhps in African *Plasmodium falciparum* malaria. PLoS Med..

[61] Kupper C (2016). A supergene determines highly divergent male reproductive morphs in the ruff. Nat. Genet..

[62] Alexander RD (1962). Evolutionary change in Cricket acoustical communication. Evolution.

[63] Ragge, D. R. *The Wing-Venation of the Orthoptera Saltatoria: With Notes on Dictyopteran Wing-venation* (British Museum, Natural History, London, 1955).

[64] Gwynne, D. T. Phylogeny of the Ensifera (Orthoptera): a hypothesis supporting multiple origins of acoustical signalling, complex spermatophores and maternal care in Crickets, Katydids, and Weta. *J. Orthop. Res.* **4**, 203–218 (1995).

[65] Desutter-Grandcolas L, Robillard T (2003). Phylogeny and the evolution of calling songs in Gryllus (Insecta, Orthoptera, Gryllidae). Zool. Scr..

[66] Wiens JJ (2001). Widespread loss of sexually selected traits: how the peacock lost its spots. Trends Ecol. Evol..

[67] Ledon-Rettig CC, Zattara EE, Moczek AP (2017). Asymmetric interactions between doublesex and tissue- and sex-specific target genes mediate sexual dimorphism in beetles. Nat. Commun..

[68] Ito Y (2013). The role of doublesex in the evolution of exaggerated horns in the Japanese rhinoceros beetle. EMBO Rep..

[69] Palmer DH, Kronforst MR (2020). A shared genetic basis of mimicry across swallowtail butterflies points to ancestral co-option of doublesex. Nat. Commun..

[70] Komata S, Lin CP, Iijima T, Fujiwara H, Sota T (2016). Identification of doublesex alleles associated with the female-limited Batesian mimicry polymorphism in *Papilio memnon*. Sci. Rep..

[71] Prakash, A. & Monteiro, A. Doublesex mediates the development of sex-specific pheromone organs in Bicyclus butterflies via multiple mechanisms. *Mol. Biol. Evol*. **37**, 1694–1707(2020). 10.1093/molbev/msaa039 PMC7253200 32077943

[72] Hoffmann AA, Sgro CM (2011). Climate change and evolutionary adaptation. Nature.

[73] Rohlf FJ (2015). The tps series of software. Hystrix.

[74] Klingenberg CP (2011). MorphoJ: an integrated software package for geometric morphometrics. Mol. Ecol. Resour..

[75] Li H (2009). The sequence alignment/map format and SAMtools. Bioinformatics.

[76] Li H, Durbin R (2009). Fast and accurate short read alignment with Burrows-Wheeler transform. Bioinformatics.

[77] McKenna A (2010). The Genome Analysis Toolkit: a MapReduce framework for analyzing next-generation DNA sequencing data. Genome Res..

[78] Van der Auwera GA (2013). From FastQ data to high confidence variant calls: the Genome Analysis Toolkit best practices pipeline. Curr. Protoc. Bioinformatics.

[79] Kim KW (2019). Genetics and evidence for balancing selection of a sex-linked colour polymorphism in a songbird. Nat. Commun..

[80] Purcell S (2007). PLINK: a tool set for whole-genome association and population-based linkage analyses. Am. J. Hum. Genet..

[81] Retief JD (2000). Phylogenetic analysis using PHYLIP. Methods Mol. Biol..

[82] Kumar S, Stecher G, Li M, Knyaz C, Tamura K (2018). MEGA X: molecular evolutionary genetics analysis across computing platforms. Mol. Biol. Evol..

[83] Patterson N, Price AL, Reich D (2006). Population structure and eigenanalysis. PLoS Genet..

[84] Wickham, H. *ggplot2: Elegant Graphics for Data Analysis*. *Use R*, 1-212 (Springer, 2009).

[85] Alexander DH, Novembre J, Lange K (2009). Fast model-based estimation of ancestry in unrelated individuals. Genome Res..

[86] Danecek P (2011). The variant call format and VCFtools. Bioinformatics.

[87] Barrett JC, Fry B, Maller J, Daly MJ (2005). Haploview: analysis and visualization of LD and haplotype maps. Bioinformatics.

[88] Korneliussen TS, Albrechtsen A, Nielsen R (2014). ANGSD: analysis of next generation sequencing data. BMC Bioinformatics..

[89] Lischer HE, Excoffier L (2012). PGDSpider: an automated data conversion tool for connecting population genetics and genomics programs. Bioinformatics.

[90] Excoffier L, Laval G, Schneider S (2007). Arlequin (version 3.0): an integrated software package for population genetics data analysis. Evol. Bioinformatics Online.

[91] Keightley PD (2009). Analysis of the genome sequences of three Drosophila melanogaster spontaneous mutation accumulation lines. Genome Res..

[92] Mousseau TA, Roff DA (1995). Genetic and environmental contributions to geographic-variation in the ovipositor length of a cricket. Ecology.

[93] Chen K (2009). BreakDancer: an algorithm for high-resolution mapping of genomic structural variation. Nat. Methods.

[94] Wang K (2014). Genome-wide variation within and between wild and domestic yak. Mol. Ecol. Resour..

[95] Abyzov, A., Urban, A. E., Snyder, M. & Gerstein, M. CNVnator: an approach to discover, genotype, and characterize typical and atypical CNVs from family and population genome sequencing. *Genome Res.* **21**, 974–984 (2011). 10.1101/gr.114876.110 PMC3106330 21324876

[96] Lou, H. et al. Copy number variations and genetic admixtures in three Xinjiang ethnic minority groups. *Eur. J. Hum. Genet*. **23**, 536–542 (2015). 10.1038/ejhg.2014.134 PMC4666576 25026903

[97] Zhang X (2016). Genome-wide patterns of copy number variation in the Chinese yak genome. BMC Genomics.

[98] Redon, R. et al. Global variation in copy number in the human genome. *Nature* **444**, 444–454 (2006). 10.1038/nature05329 PMC2669898 17122850

[99] Pezer, Z., Harr, B., Teschke, M., Babiker, H. & Tautz, D. Divergence patterns of genic copy number variation in natural populations of the house mouse (*Mus musculus domesticus*) reveal three conserved genes with major population-specific expansions. *Genome Res.* **25**, 1114–1124 (2015). 10.1101/gr.187187.114 PMC4509996 26149421

[100] Nei M, Roychoudhury AK (1972). Gene differences between Caucasian, Negro, and Japanese populations. Science.

[101] Wakeley J (1996). The variance of pairwise nucleotide differences in two populations with migration. Theor. Popul. Biol..

[102] Pfeifer B, Wittelsburger U, Ramos-Onsins SE, Lercher MJ (2014). PopGenome: an efficient Swiss army knife for population genomic analyses in R. Mol. Biol. Evol..

[103] Bairoch A, Apweiler R (2000). The SWISS-PROT protein sequence database and its supplement TrEMBL in 2000. Nucleic Acids Res..

[104] Martin M (2011). Cutadapt removes adapter sequences from high-throughput sequencing reads. EMBnet J..

[105] Kopylova E, Noe L, Touzet H (2012). SortMeRNA: fast and accurate filtering of ribosomal RNAs in metatranscriptomic data. Bioinformatics.

[106] Pertea M, Kim D, Pertea GM, Leek JT, Salzberg SL (2016). Transcript-level expression analysis of RNA-seq experiments with HISAT, StringTie and Ballgown. Nat. Protoc..

[107] Robinson MD, McCarthy DJ, Smyth G (2010). K. edgeR: a Bioconductor package for differential expression analysis of digital gene expression data. Bioinformatics.

