# Supplementary material for: Rapid parallel adaptation despite gene flow in silent crickets
Source: Nat Commun. 2021 Jan 4;12:50. doi: 10.1038/s41467-020-20263-4 (PMC7782688; doi:10.1038/s41467-020-20263-4)
Supplement: Supplementary file 1 — Supplementary information [file 41467_2020_20263_MOESM1_ESM.pdf]

Supplementary information for:

Rapid parallel adaptation despite gene flow in silent crickets

Xiao Zhang[1†*], Jack G. Rayner[1†], Mark Blaxter[2], Nathan W. Bailey[1*]

[1] School of Biology, University of St Andrews, St Andrews, Fife KY16 9TH, UK. [2] Tree of Life, Wellcome Sanger Institute, Cambridge, CB10 1SA, UK. [†] These authors contributed equally: Xiao Zhang, Jack G. Rayner

*email: xz42@st-andrews.ac.uk, nwb3@st-andrews.ac.uks

Table of contents

Supplementary Figures 1-33
Supplementary Tables 1-10

Supplementary Figures

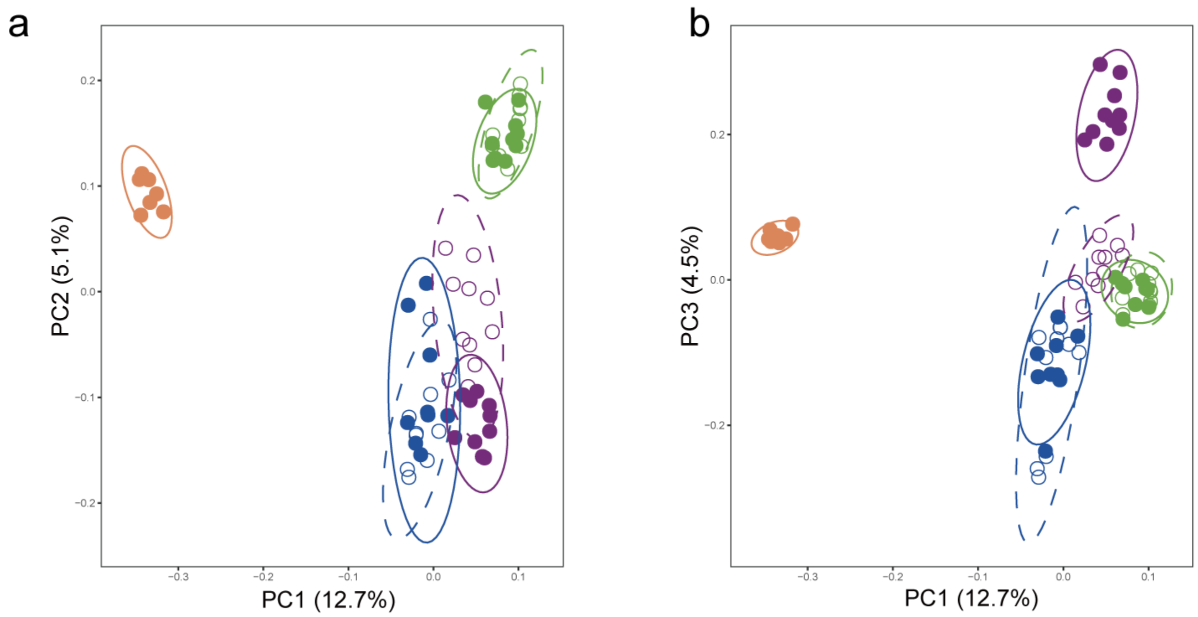

Supplementary Fig. 1 | Principal component (PC) plots showing genomic distance of resequenced *Teleogryllus oceanicus* samples, for autosomal markers only. a, Principal components 1 vs. 2, **b,** PCs 1 vs. 3. Genomic variance explained for PC1: 12.7%, PC2: 5.1%, PC3: 4.5%. Colour and symbol scheme follow that in the Main Text and population codes follow Supplementary Table 3: purple = Kauai males, blue = Oahu males, green = Hilo males, orange = *T. oceanicus* males from Australia. Solid circles represent normal-wing males and open circles represent flatwing males. Source data are provided as a Source Data file.

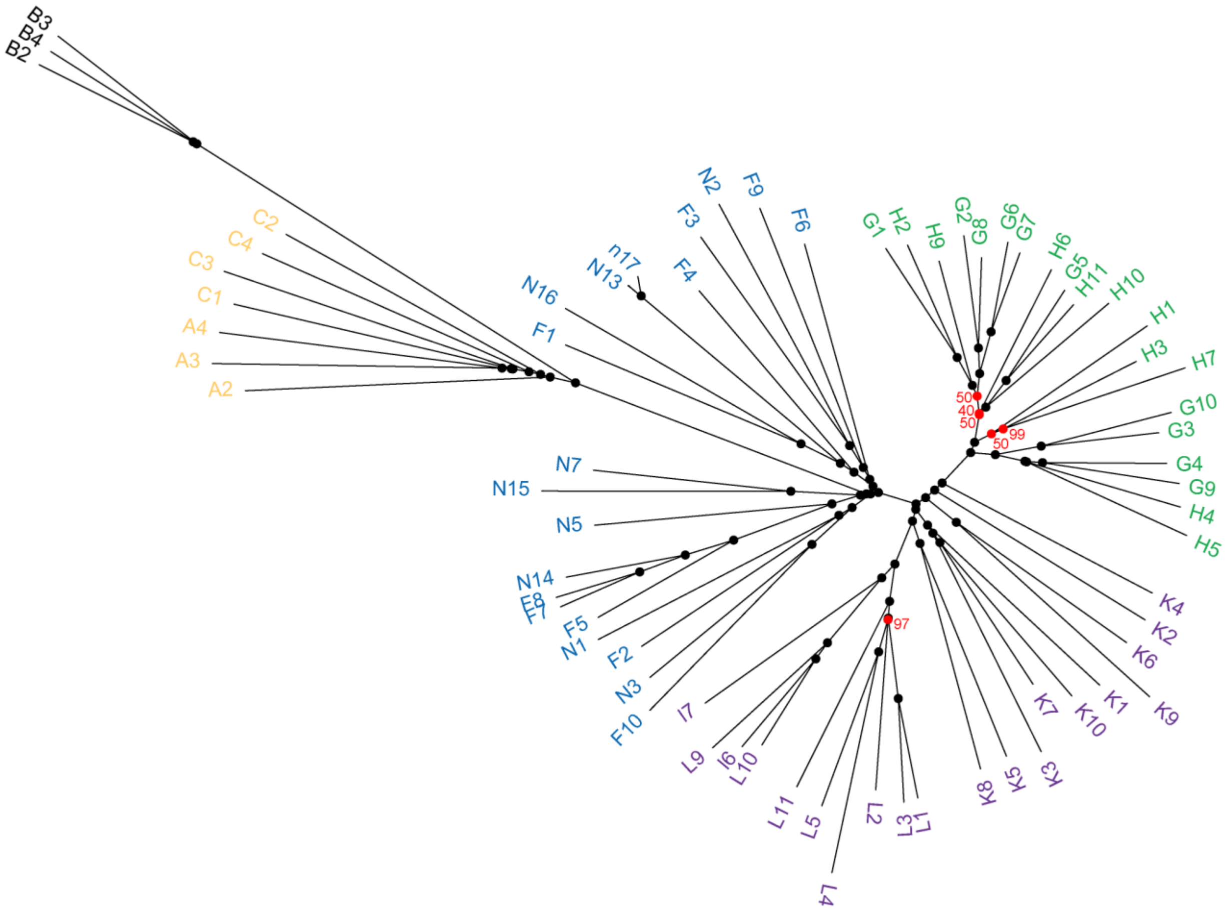

Supplementary Fig. 2 | Neighbour-joining tree with bootstrap values based on 1000 replicates constructed using only autosomal loci. Nodes with bootstrap values equal to 100% are shown by black dots. Those less than 100% are shown by red dots with bootstrap values. Colour scheme follows that in the Main Text and population codes follow Supplementary Table 3: purple = Kauai males, blue = Oahu males, green = Hilo males, orange = *T. oceanicus* males from Australia, black = *T. commodus* males from Australia.

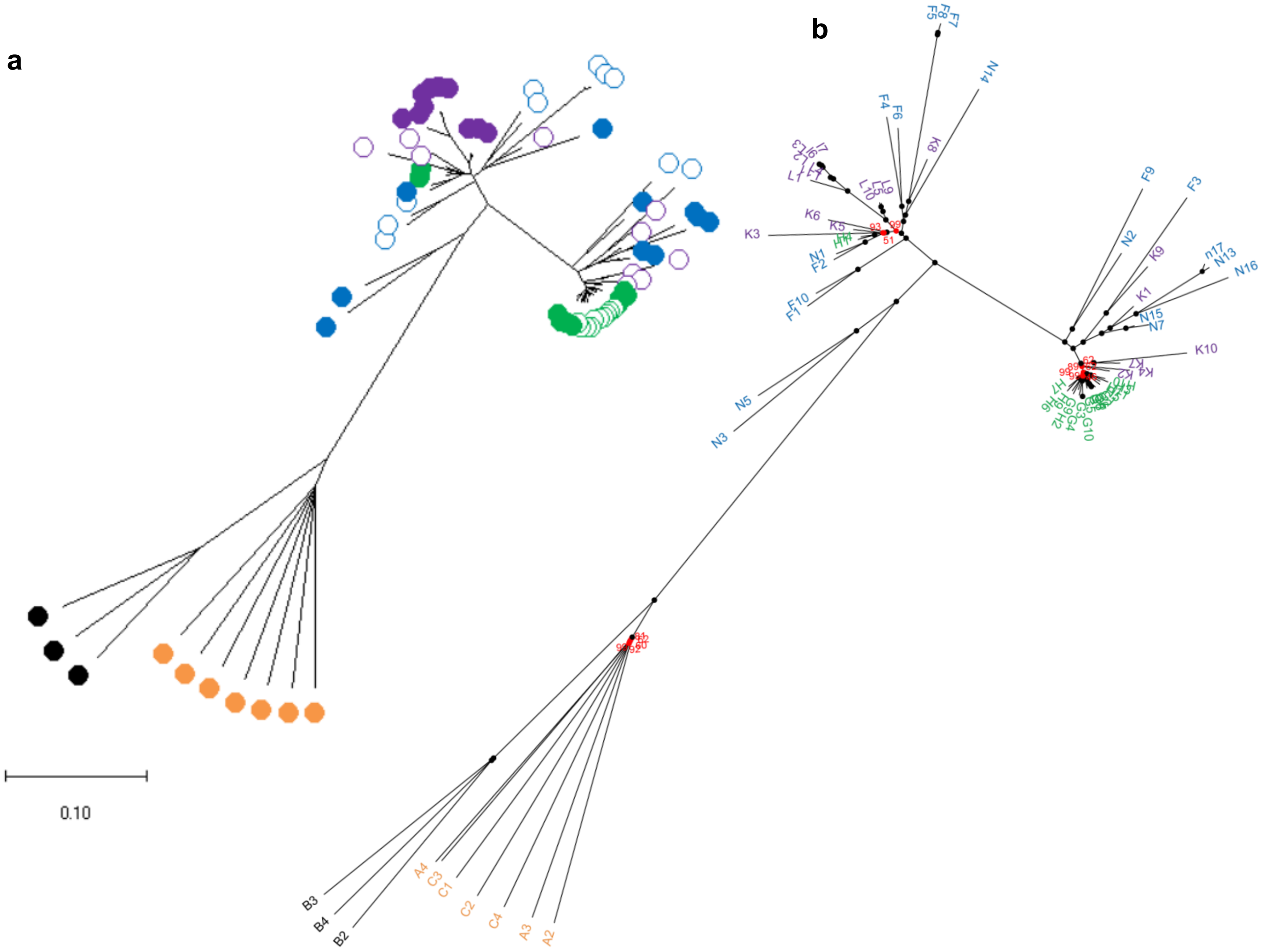

Supplementary Fig. 3 | Neighbor-joining phylogenetic trees. a, Tree constructed using X chromosome SNPs. Colour and symbol scheme follow that in the Main Text and population codes follow Supplementary Table 3: purple = Kauai males, blue = Oahu males, green = Hilo males, orange = *T. oceanicus* males from Australia, black = *T. commodus* males from Australia. Solid circles represent normal-wing males and open circles represent flatwing males. **b,** Tree redrawn with bootstrap values based on 1000 replicates. Nodes with bootstrap values equal to 100% are shown by black dots. Those less than 100% are shown by red dots with bootstrap values).

Supplementary Fig. 4 | Distribution among 18 linkage groups of Patterson's *D* statistic.

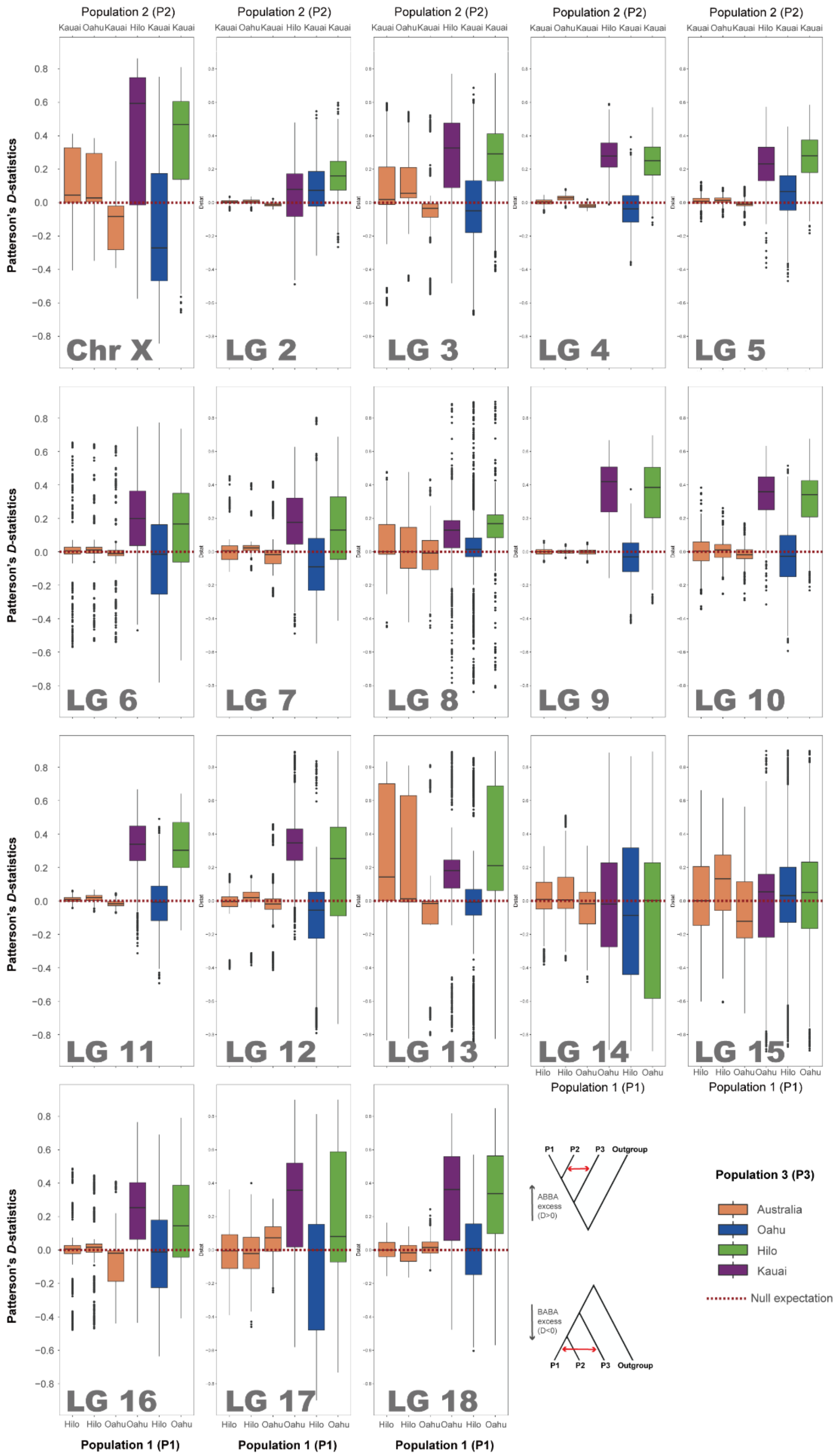

Sites used in thes test are derived in *T. oceanicus* compared to the outgroup (*T. commodus*), but differ among P1 and P2 *T. oceanicus* individuals. Tests with Australian *T. oceanicus* populations are included as controlled comparisons (left three bars) and the plot shows all possible topological permutations of the three Hawaiian island populations relevant for the ABBA-BABA test (right three bars). The strongest signals of gene flow were found on the X chromosome (LG1) controlling flatwing phenotype. Most autosomal LGs showed patterns consistent with the X chromosome. n = 700 independent *D* values for each Australian control; n = 1,000 each for Kauai and Hilo; n = 2,000 for Oahu. Statistical details are provided in Supplementary Data 10. Box plots show upper quartile, median, and lower quartile values, 1.5x interquartile ranges, and outliers.
Source data are provided as a Source Data file.

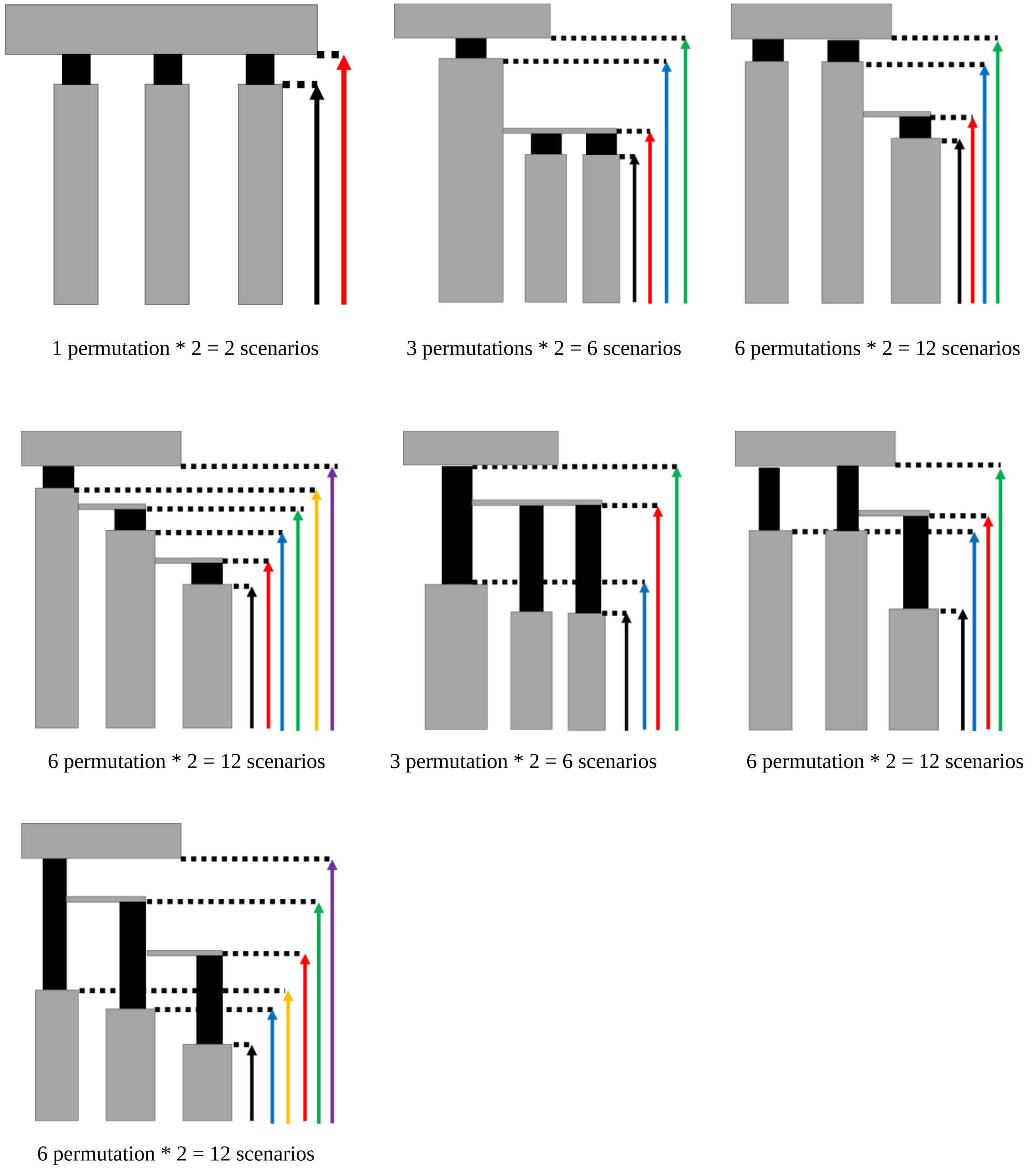

Supplementary Fig. 5 | Schematic diagrams of 62 models of demographic history tested for resequenced Hawaiian crickets. Multiple scenarios are represented for each diagram reflecting the different permutations of branching patterns possible among the three island populations, all either with or without symmetric gene flow. In each figure, time is ordered from ancient to recent from top to bottom, the top rectangle represents a putative ancestral population, solid black columns indicate population bottlenecks, and the dashed lines with different coloured arrows illustrate different time points (in generations) inferred by the model.

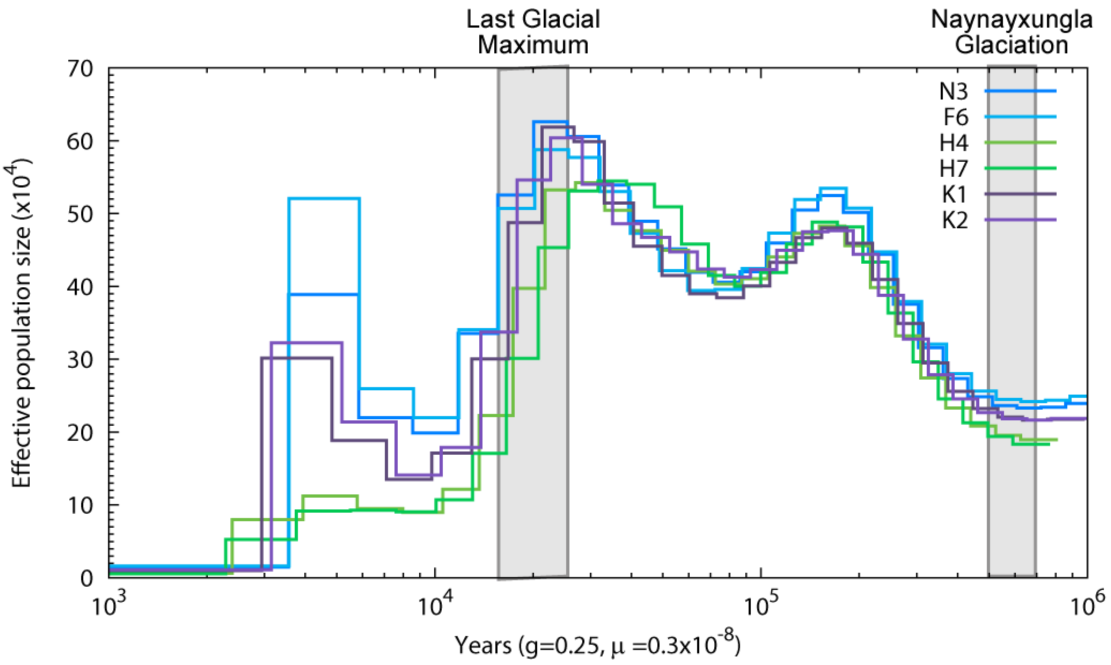

Supplementary Fig. 6 | Historical effective population size of Hawaii *T. oceanicus* populations inferred using PSMC analyses. Light gray shading indicates the last glacial maximum and the Naynayxungla Glaciation. Two wild-sampled individuals from each island population were used for the analysis. Each coloured line represents one individual, with different shades of purple = Kauai males, blue = Oahu males, and green = Hilo males.

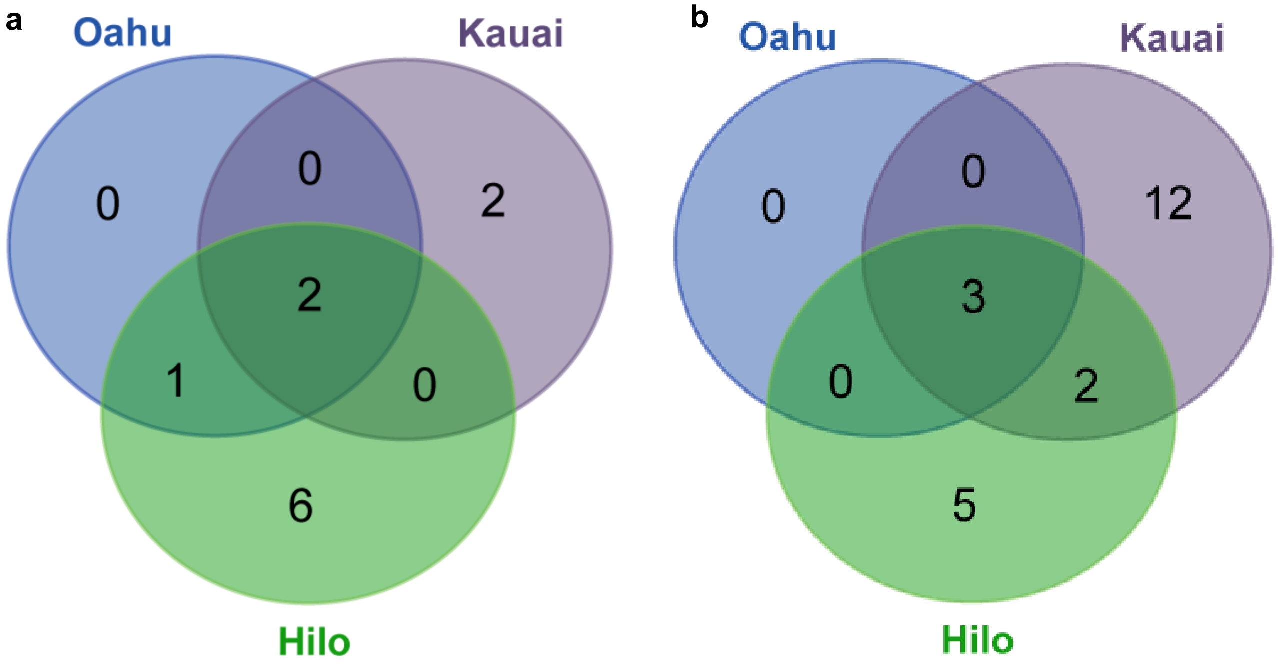

Supplementary Fig. 7 | Venn diagrams showing the distribution of candidate flatwing scaffolds that were shared vs. unshared among Hawaiian *T. oceanicus* populations. a, Candidate flatwing scaffolds detected using the strictest FDR criteria. **b**, Candidate flatwing scaffolds detected using more permissive FDR criteria. See Main Text for details of different criteria.

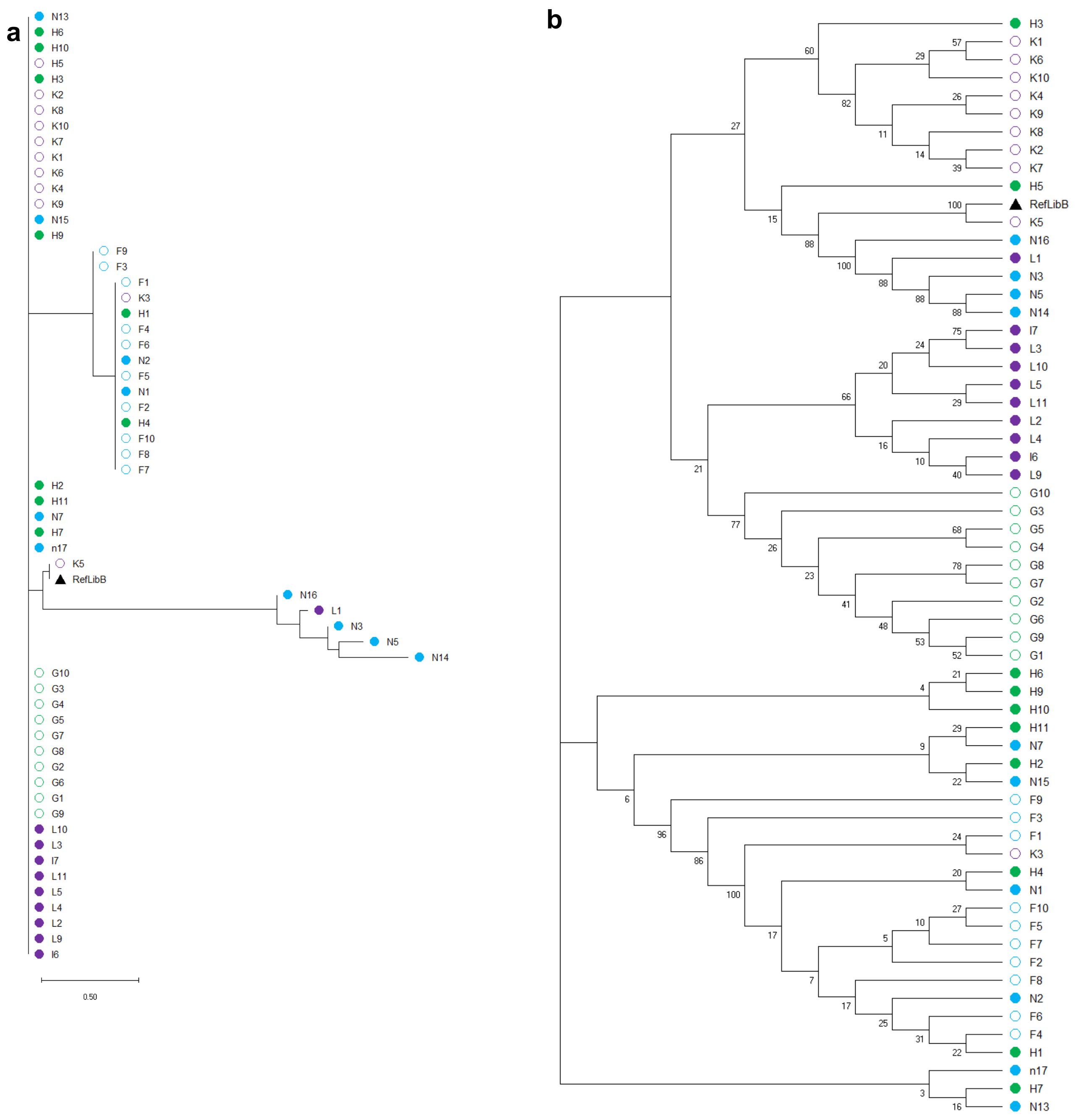

Supplementary Fig. 8 | Maximum-likelihood trees constructed using two candidate *flatwing* scaffolds shared by three Hawaiian populations of *T. oceanicus.* a, Tree drawn to scale, with bar representing the number of nucleotide substitutions per SNP site. (LNL=-112587.280) **b**, Consensus tree showing bootstrap values obtained from 1000 replicates. The two candidate flatwing scaffolds are scaffold 18404 and scaffold 6636. They span 2.61 Mb and contain 15,618 SNPs. Colour and symbol scheme follow that in the Main Text and population codes follow Supplementary Table 3: purple = Kauai males, blue = Oahu males, green = Hilo males. Solid circles represent normal-wing males, open circles represent flatwing males, and the triangle shows the individual used as the reference genome in the analysis.

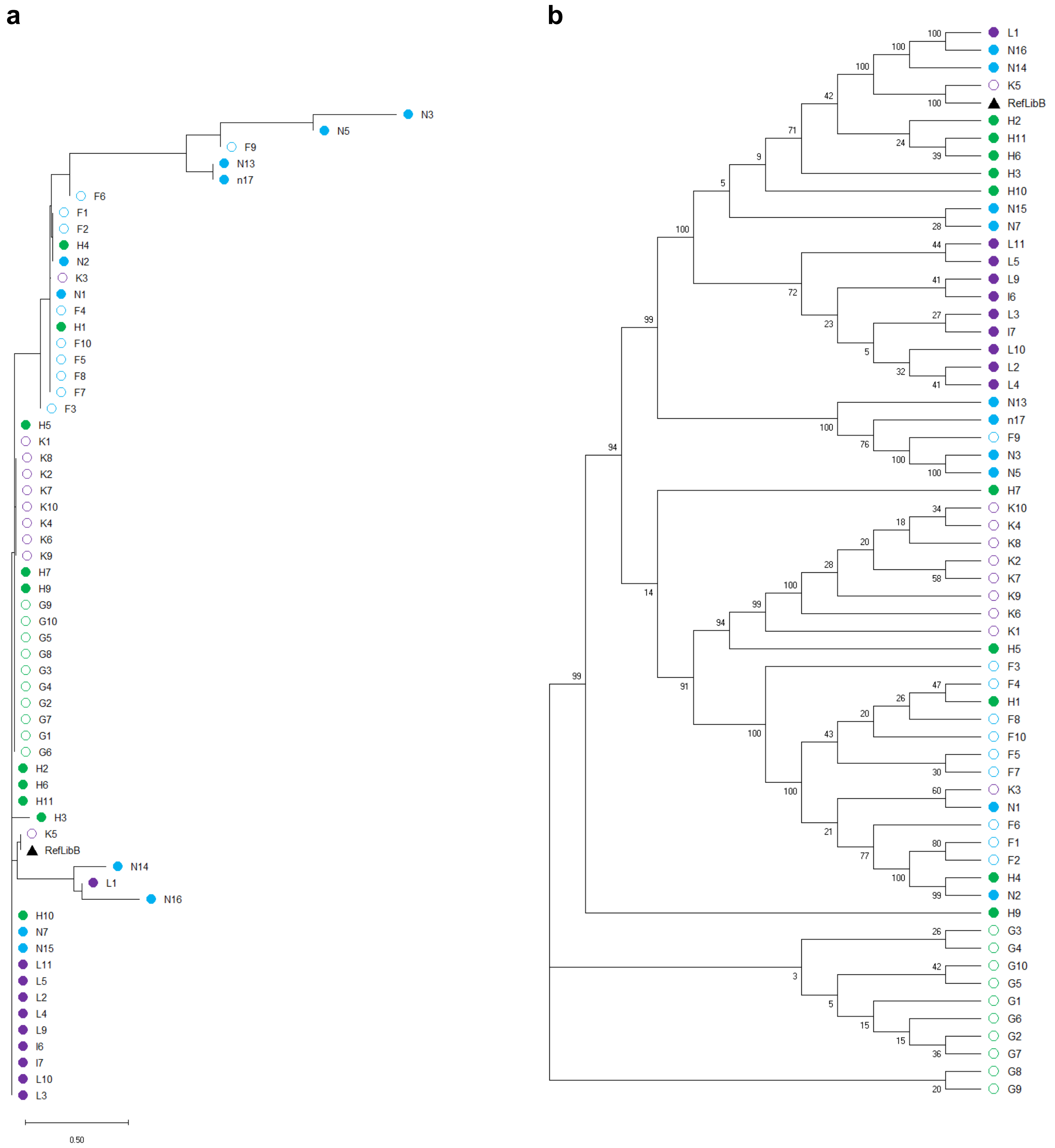

Supplementary Fig. 9 | Maximum-likelihood trees constructed using three candidate *flatwing* scaffolds (detected using more permissive FDR criteria). a, Tree drawn to scale, with bar representing the number of nucleotide substitutions per SNP site. (LnL=-343996.444) **b**, Consensus tree showing bootstrap values obtained from 1000 replicates. The three candidate flatwing scaffolds are: scaffold 18404, Contig2899_pilon, and scaffold 6636. They span 5.25 Mb and contain 36,254 SNPs. Colour and symbol scheme follow that in the Main Text and population codes follow Supplementary Table 3: purple = Kauai males, blue = Oahu males, green = Hilo males. Solid circles represent normal-wing males, open circles represent flatwing males, and the triangle shows the individual used as the reference genome in the analysis.

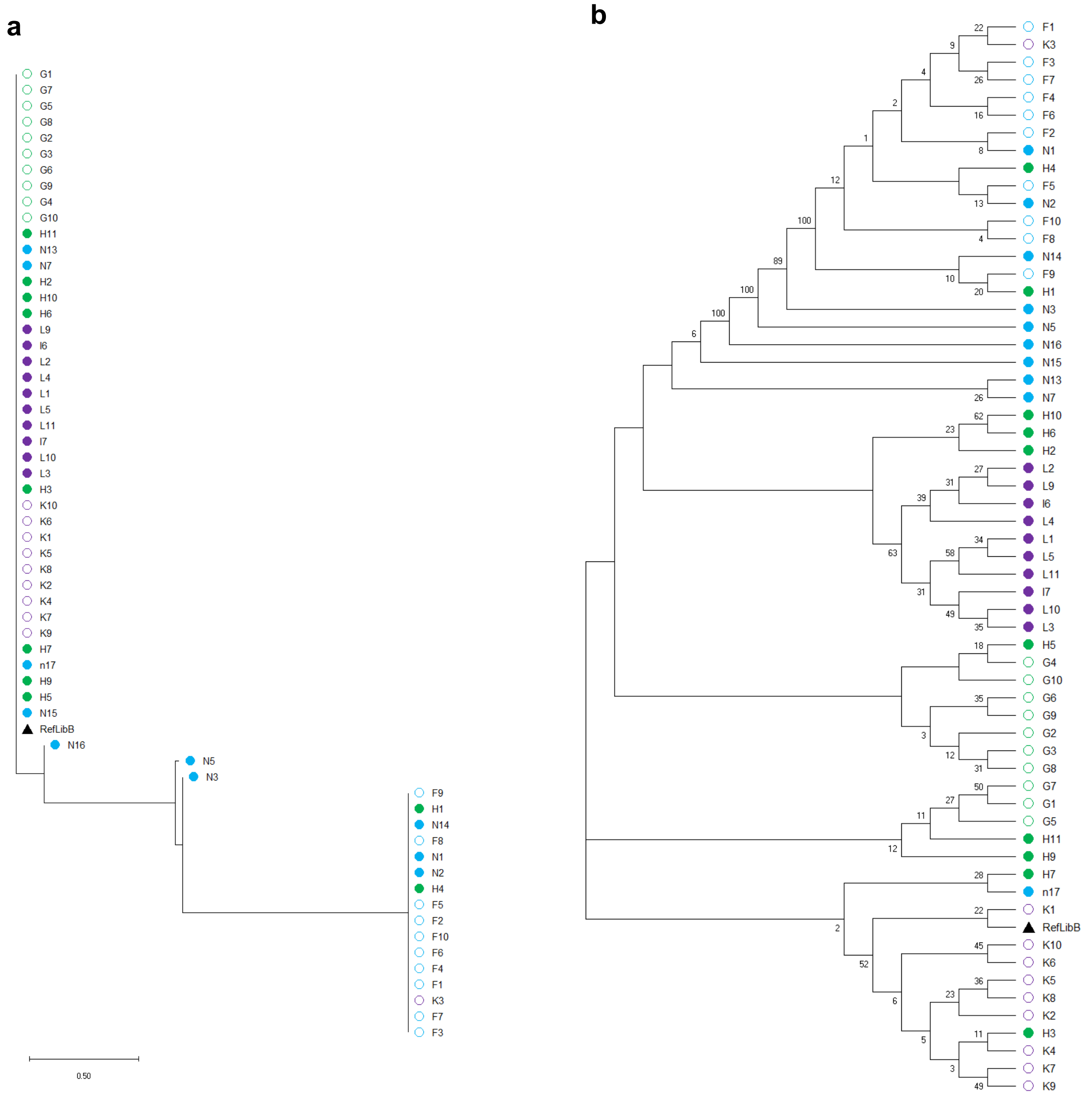

Supplementary Fig. 10 | Maximum-likelihood trees for the putative hotspot of genomic adaptation, flatwing-associated scaffold 18404 containing *dsx*. a, Tree drawn to scale, with bar representing the number of nucleotide substitutions per SNP site. (LNL=-32772.683) **b**, Consensus tree showing bootstrap values obtained from 1000 replicates. The scaffold spanned 1.3 Mb and contained 4,593 SNPs. Colour and symbol scheme follow that in the Main Text and population codes follow Supplementary Table 3: purple = Kauai males, blue = Oahu males, green = Hilo males. Solid circles represent normal-wing males, open circles represent flatwing males, and the triangle shows the individual used as the reference genome in the analysis.

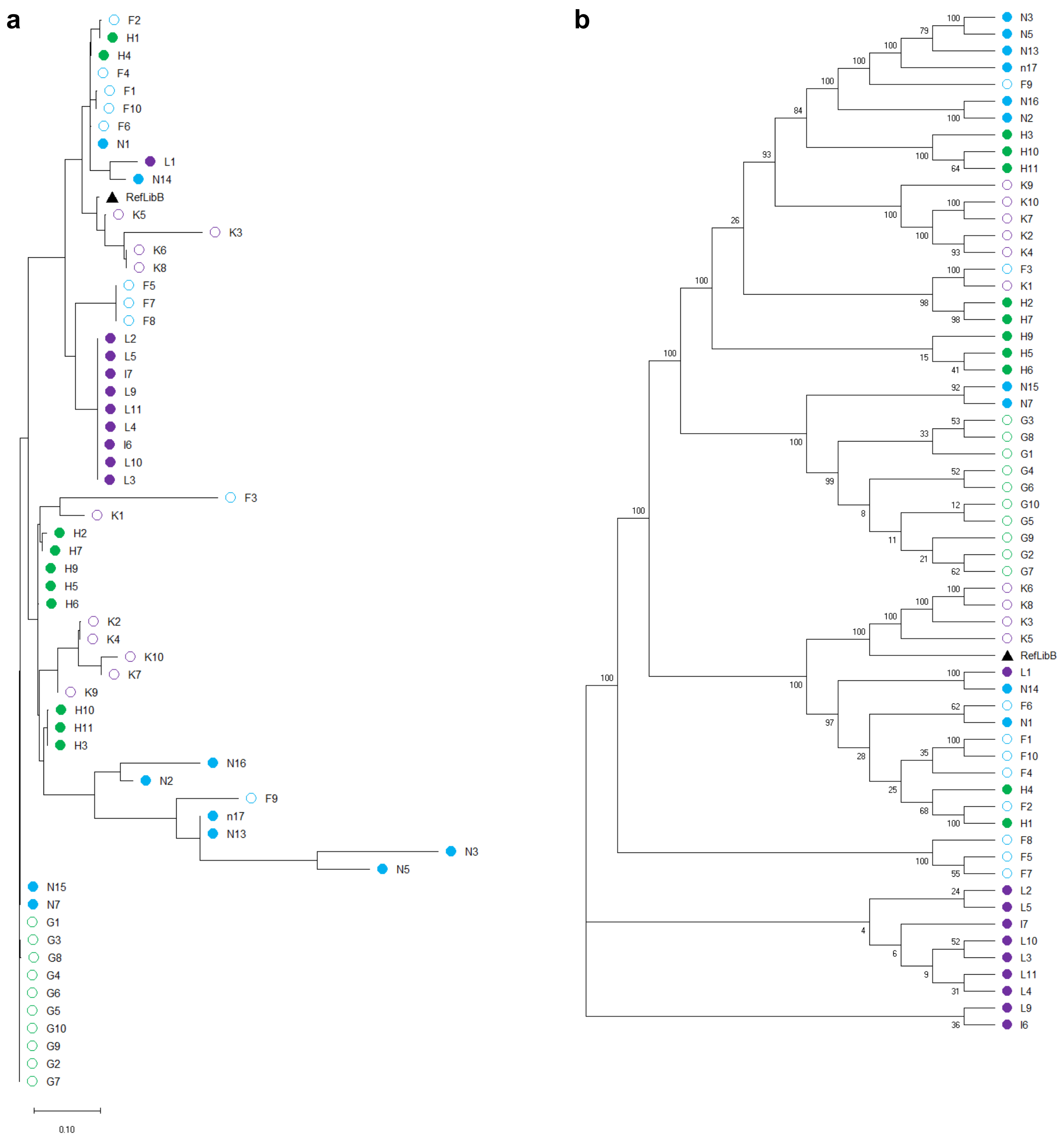

Supplementary Fig. 11 | Maximum-likelihood trees for the flanking regions (± 5 kb) of all flatwing-associated SNPs. a, Tree drawn to scale, with bar representing the number of nucleotide substitutions per SNP site. (LNL=-350428.357) **b**, Consensus tree showing bootstrap values obtained from 1000 replicates. Colour and symbol scheme follow that in the Main Text and population codes follow Supplementary Table 3: purple = Kauai males, blue = Oahu males, green = Hilo males. Solid circles represent normal-wing males, open circles represent flatwing males, and the triangle shows the individual used as the reference genome in the analysis.

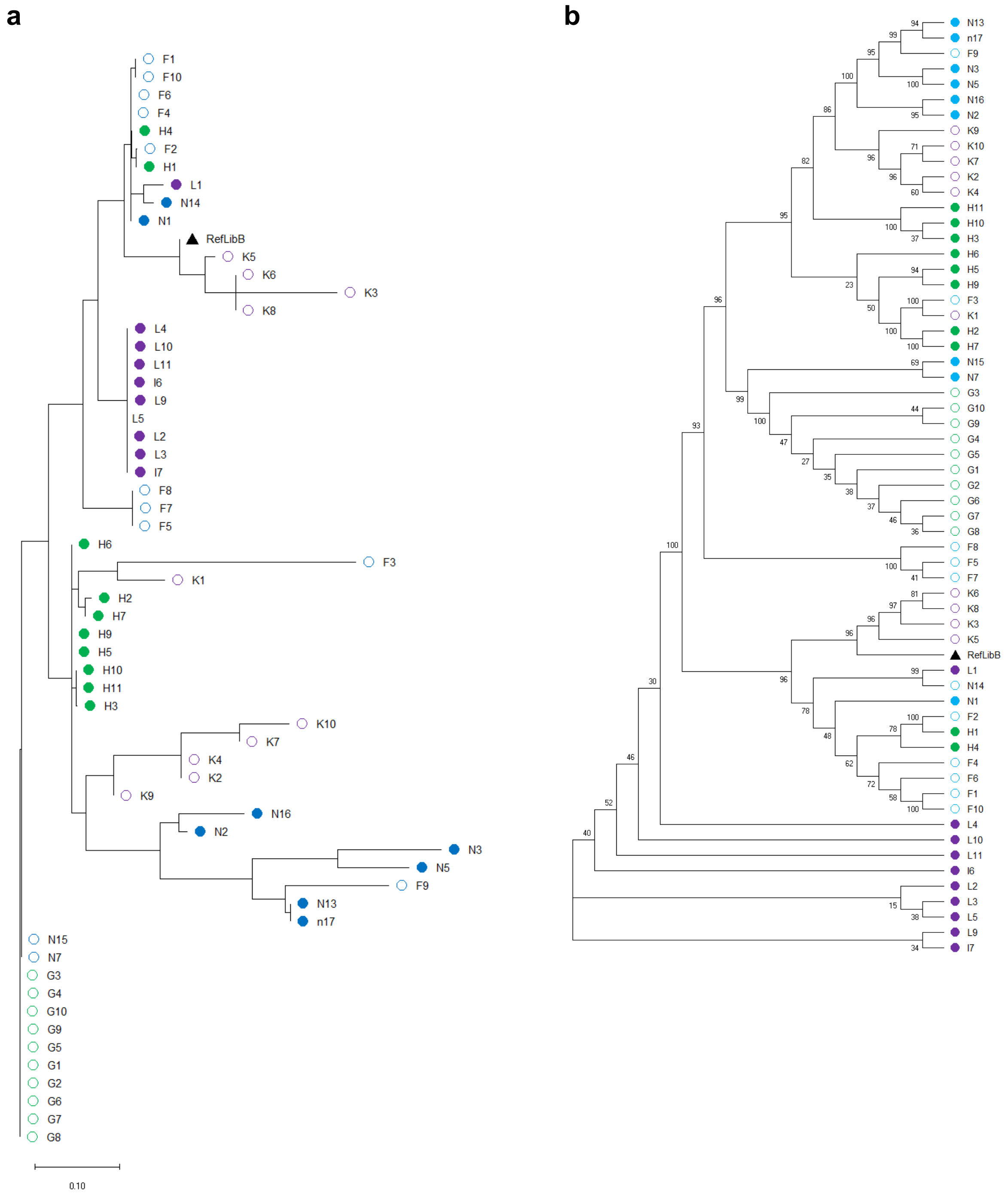

Supplementary Fig. 12 | Maximum-likelihood trees for the flanking regions (± 1 kb) of all flatwing-associated SNPs. a, Tree drawn to scale, with bar representing the number of nucleotide substitutions per SNP site. (LNL=-133620.059) **b**, Consensus tree showing bootstrap values obtained from 1000 replicates. Colour and symbol scheme follow that in the Main Text and population codes follow Supplementary Table 3: purple = Kauai males, blue = Oahu males, green = Hilo males. Solid circles represent normal-wing males, open circles represent flatwing males, and the triangle shows the individual used as the reference genome in the analysis.

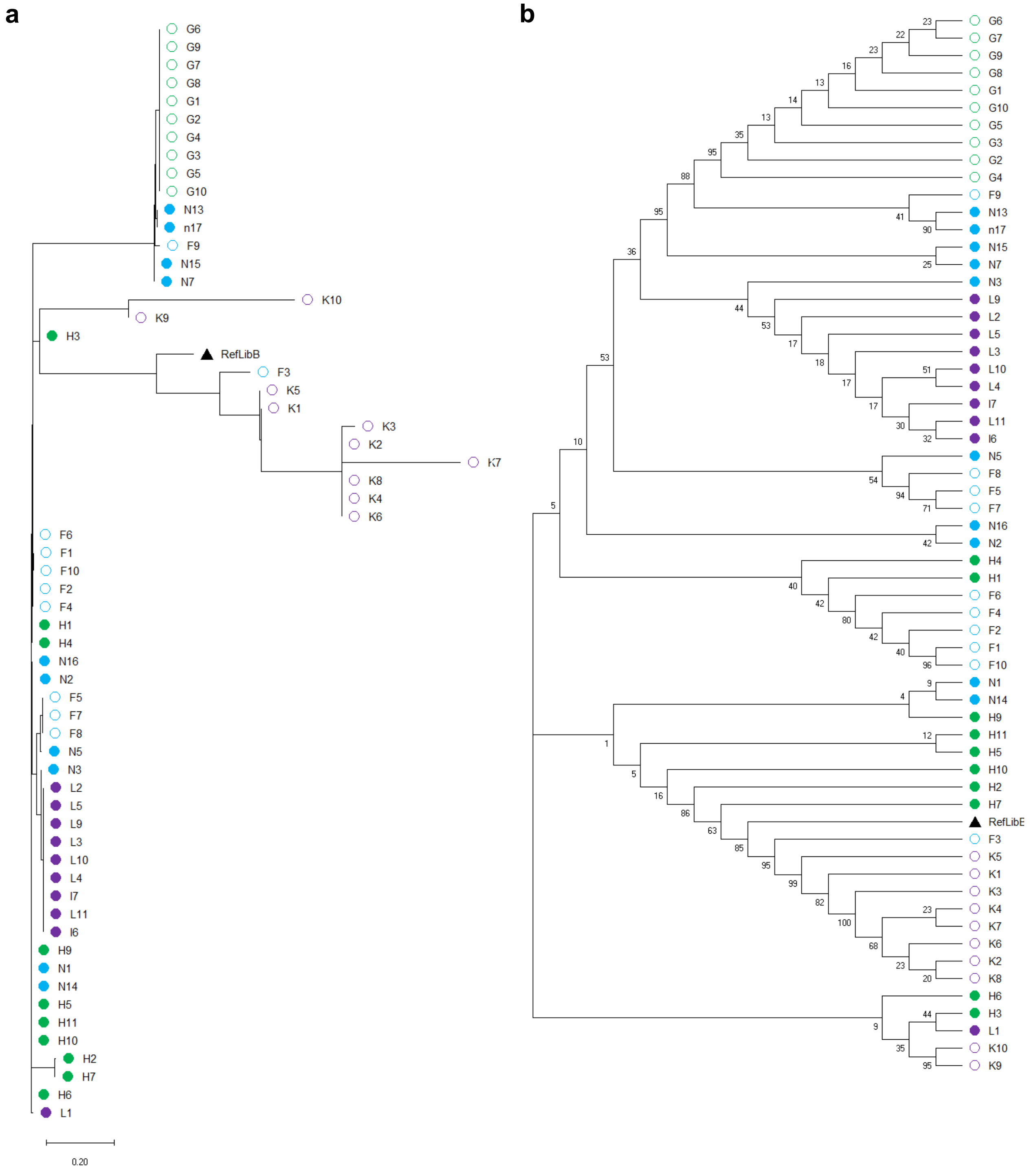

Supplementary Fig. 13 | Maximum-likelihood trees for all flatwing-associated SNPs. a, Tree drawn to scale, with bar representing the number of nucleotide substitutions per SNP site. (LnL=-11540.962) **b**, Consensus tree showing bootstrap values obtained from 1000 replicates. Colour and symbol scheme follow that in the Main Text and population codes follow Supplementary Table 3: purple = Kauai males, blue = Oahu males, green = Hilo males. Solid circles represent normal-wing males, open circles represent flatwing males, and the triangle shows the individual used as the reference genome in the analysis.

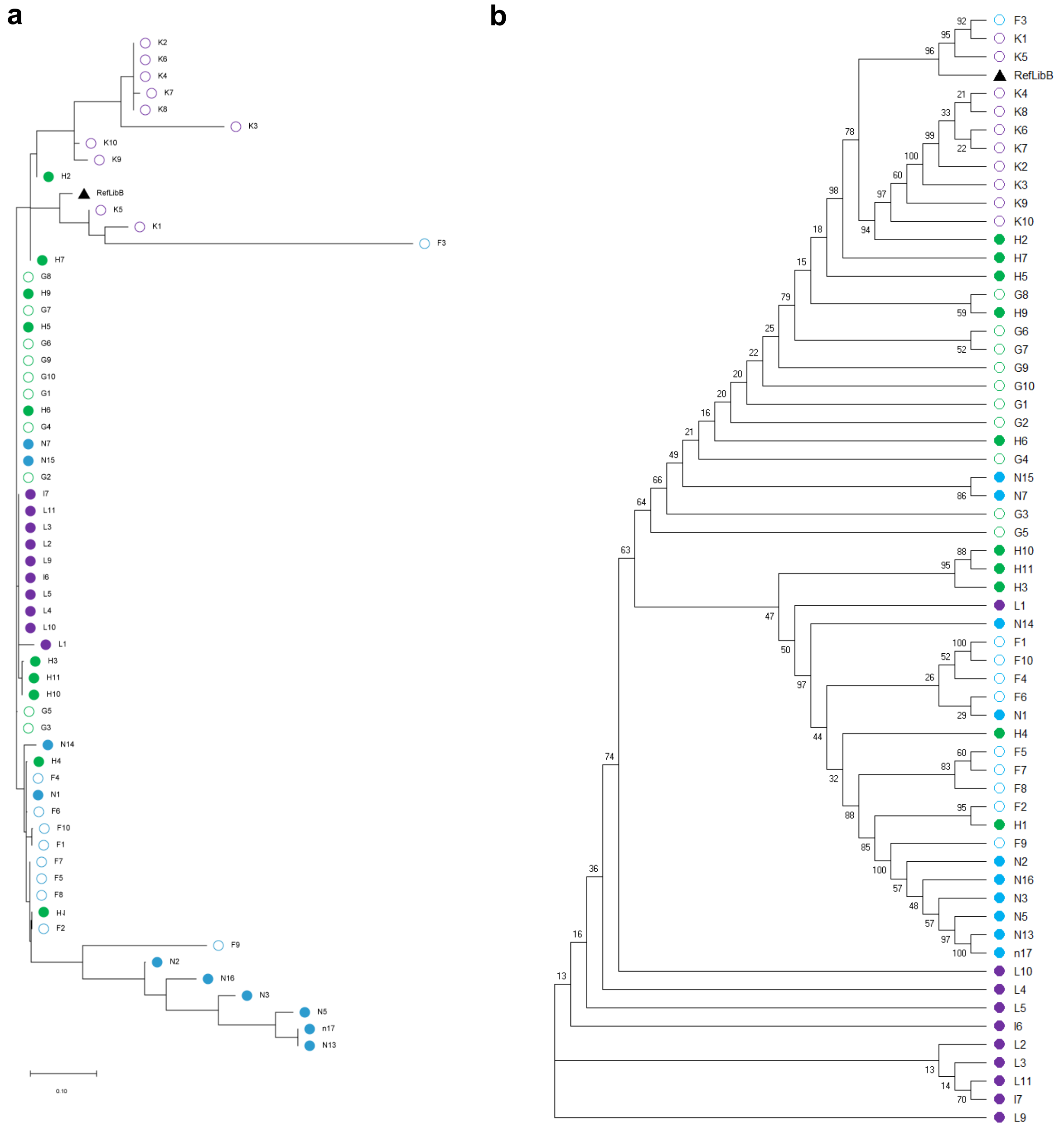

Supplementary Fig. 14 | Maximum-likelihood trees for the flanking regions (± 1 kb) of Kauai-specific flatwing-associated SNPs. a, Tree drawn to scale, with bar representing the number of nucleotide substitutions per SNP site. (LnL=-77480.985) **b**, Consensus tree showing bootstrap values obtained from 1000 replicates. Colour and symbol scheme follow that in the Main Text and population codes follow Supplementary Table 3: purple = Kauai males, blue = Oahu males, green = Hilo males. Solid circles represent normal-wing males, open circles represent flatwing males, and the triangle shows the individual used as the reference genome in the analysis.

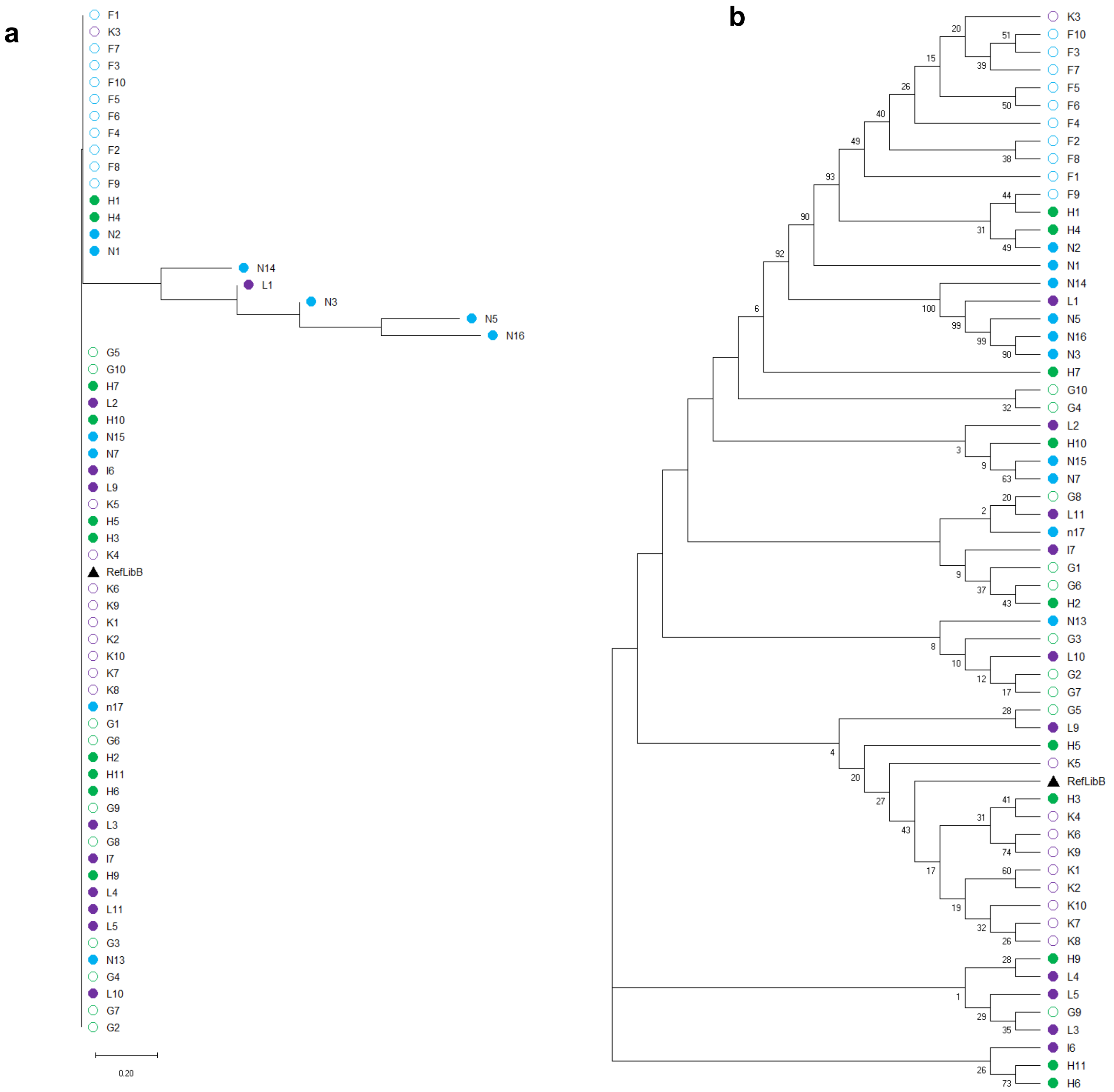

Supplementary Fig. 15 | Maximum-likelihood trees for the flanking regions (± 50 kb) of Oahu-specific flatwing-associated SNPs. a, Tree drawn to scale, with bar representing the number of nucleotide substitutions per SNP site. (LnL=-87150.000) **b**, Consensus tree showing bootstrap values obtained from 1000 replicates. Colour and symbol scheme follow that in the Main Text and population codes follow Supplementary Table 3: purple = Kauai males, blue = Oahu males, green = Hilo males. Solid circles represent normal-wing males, open circles represent flatwing males, and the triangle shows the individual used as the reference genome in the analysis.

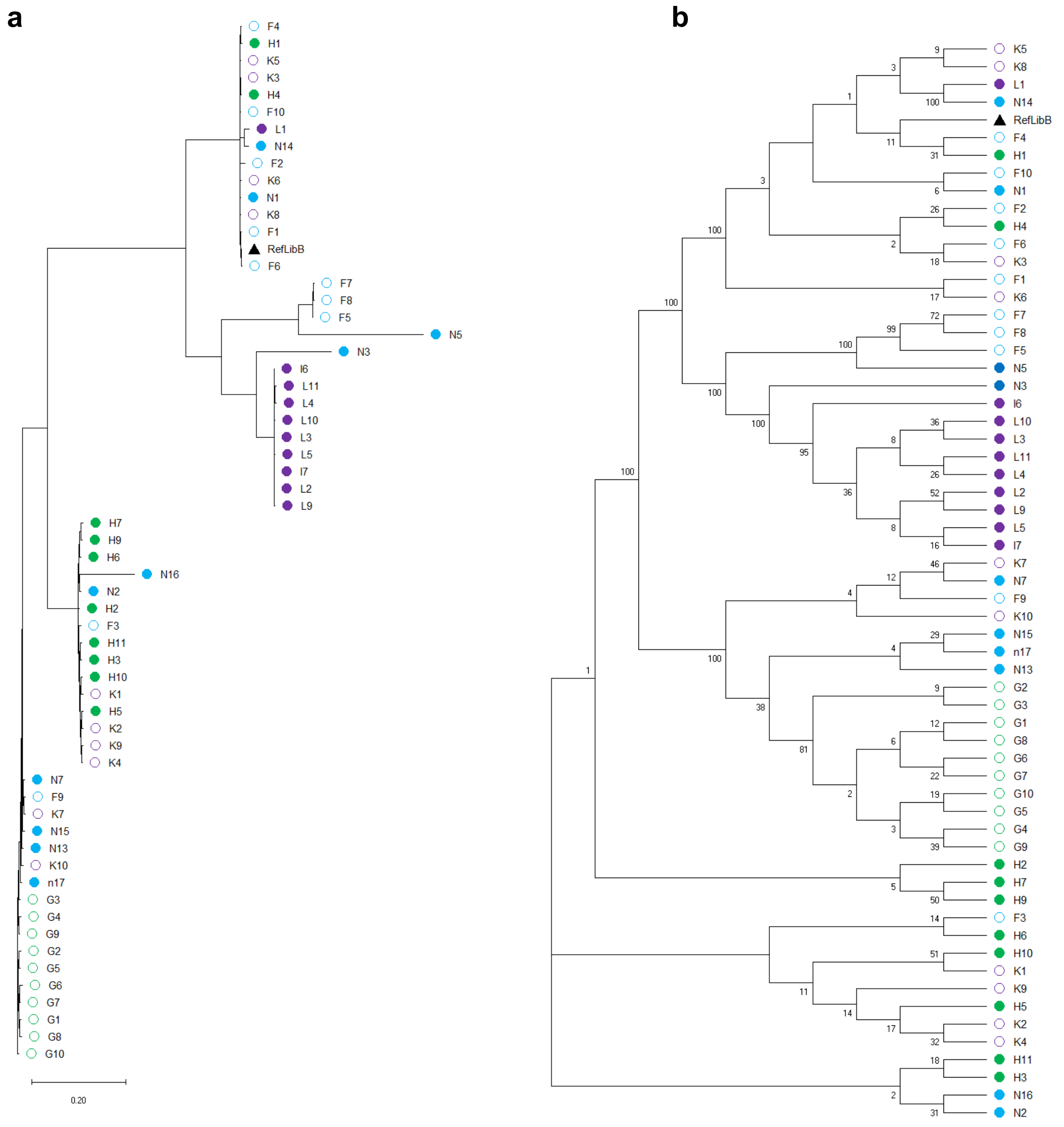

Supplementary Fig. 16 | Maximum-likelihood trees for the flanking regions (± 5 kb) of Hilo-specific flatwing-associated SNPs. **a**, Tree drawn to scale, with bar representing the number of nucleotide substitutions per SNP site. (LnL=-68262.878) **b**, Consensus tree showing bootstrap values obtained from 100 replicates. Colour and symbol scheme follow that in the Main Text and population codes follow Supplementary Table 3: purple = Kauai males, blue = Oahu males, green = Hilo males. Solid circles represent normal-wing males, open circles represent flatwing males, and the triangle shows the individual used as the reference genome in the analysis

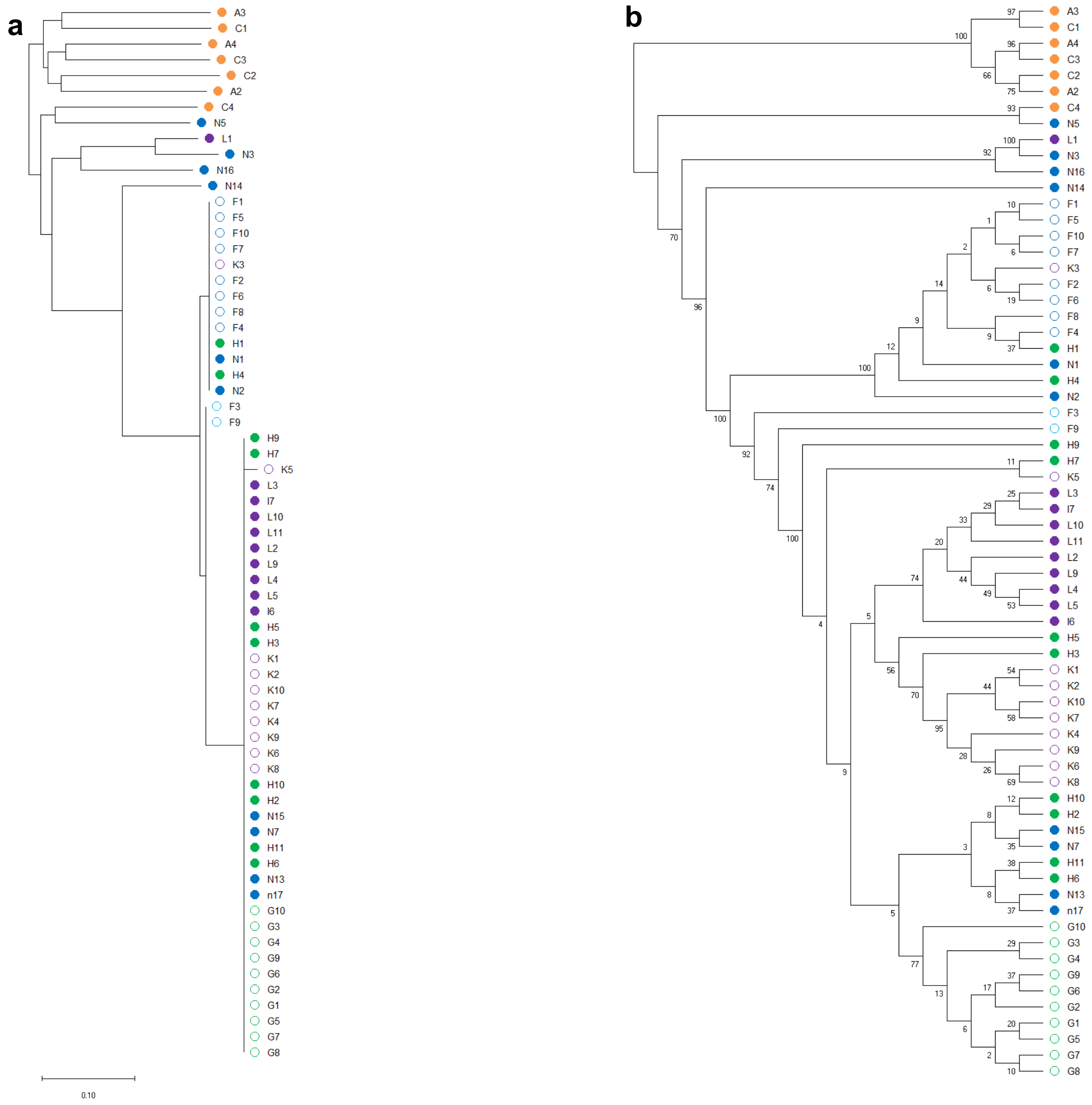

Supplementary Fig. 17 | Maximum-likelihood trees constructed using two candidate *flatwing* scaffolds shared by three Hawaiian populations of *T. oceanicus.* **a**, Tree drawn to scale, with bar representing the number of nucleotide substitutions per SNP site. (LnL=-905063.173) **b**, Consensus tree showing bootstrap values obtained from 1000 replicates. The two candidate flatwing scaffolds are scaffold 18404 and scaffold 6636. They span 2.61 Mb and contain 100,029 SNPs. Colour and symbol scheme follow that in the Main Text and population codes follow Supplementary Table 3: orange = *T. oceanicus* males from Australia, purple = Kauai males, blue = Oahu males, green = Hilo males. Solid circles represent normal-wing males, open circles represent flatwing males.

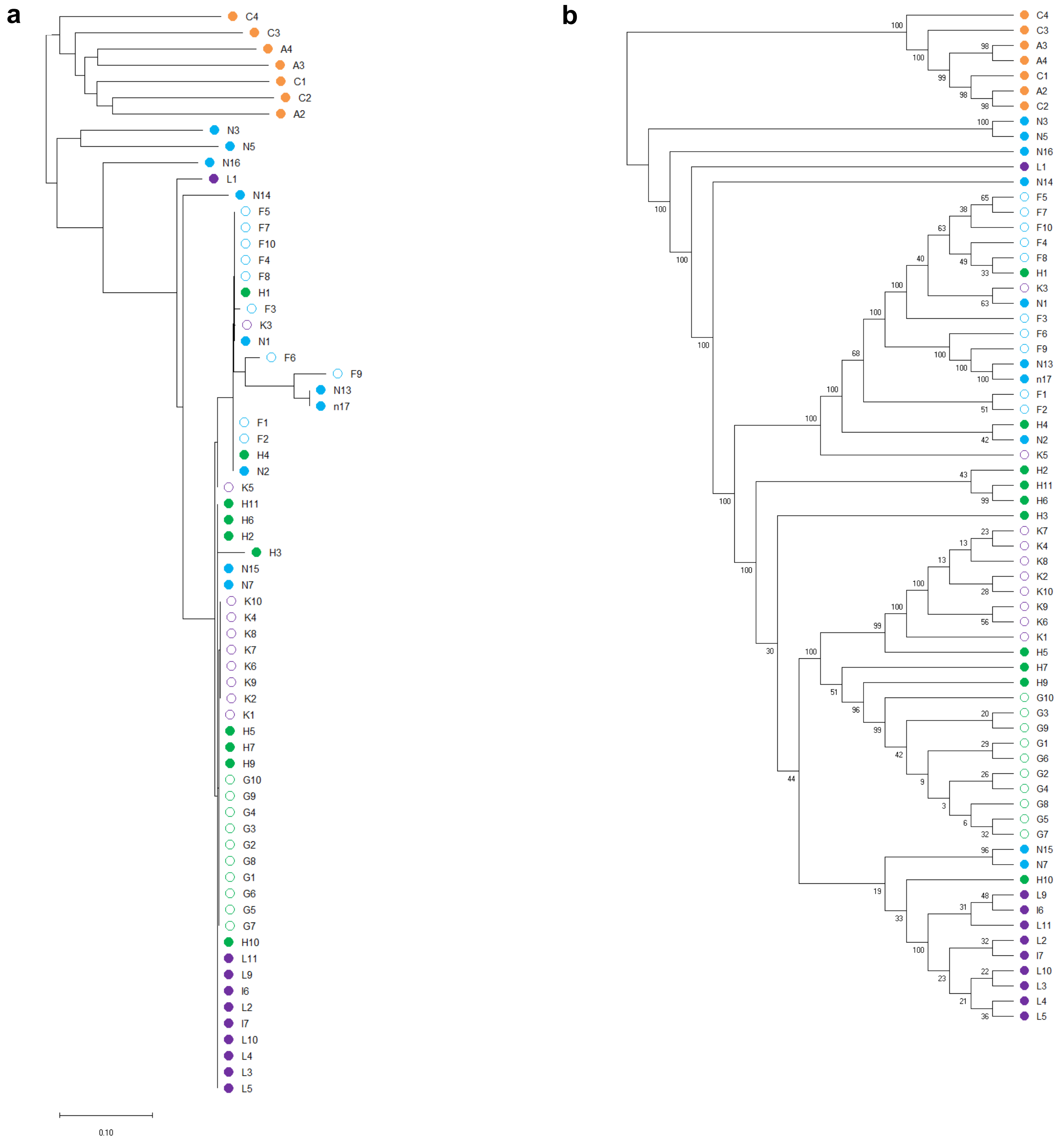

Supplementary Fig. 18 | Maximum-likelihood trees constructed using three candidate *flatwing* scaffolds (detected using more permissive FDR criteria) shared by three Hawaiian island populations of *T. oceanicus*. a, Tree drawn to scale, with bar representing the number of nucleotide substitutions per SNP site. (LnL=-2365950.419) **b**, Consensus tree showing bootstrap values obtained from 1000 replicates. The three candidate flatwing scaffolds are: scaffold 18404, Contig2899_pilon, and scaffold 6636. They span 5.25 Mb and contain 246,106 SNPs. Colour and symbol scheme follow that in the Main Text and population codes follow Supplementary Table 3: orange = *T. oceanicus* males from Australia, purple = Kauai males, blue = Oahu males, green = Hilo males. Solid circles represent normal-wing males, open circles represent flatwing males.

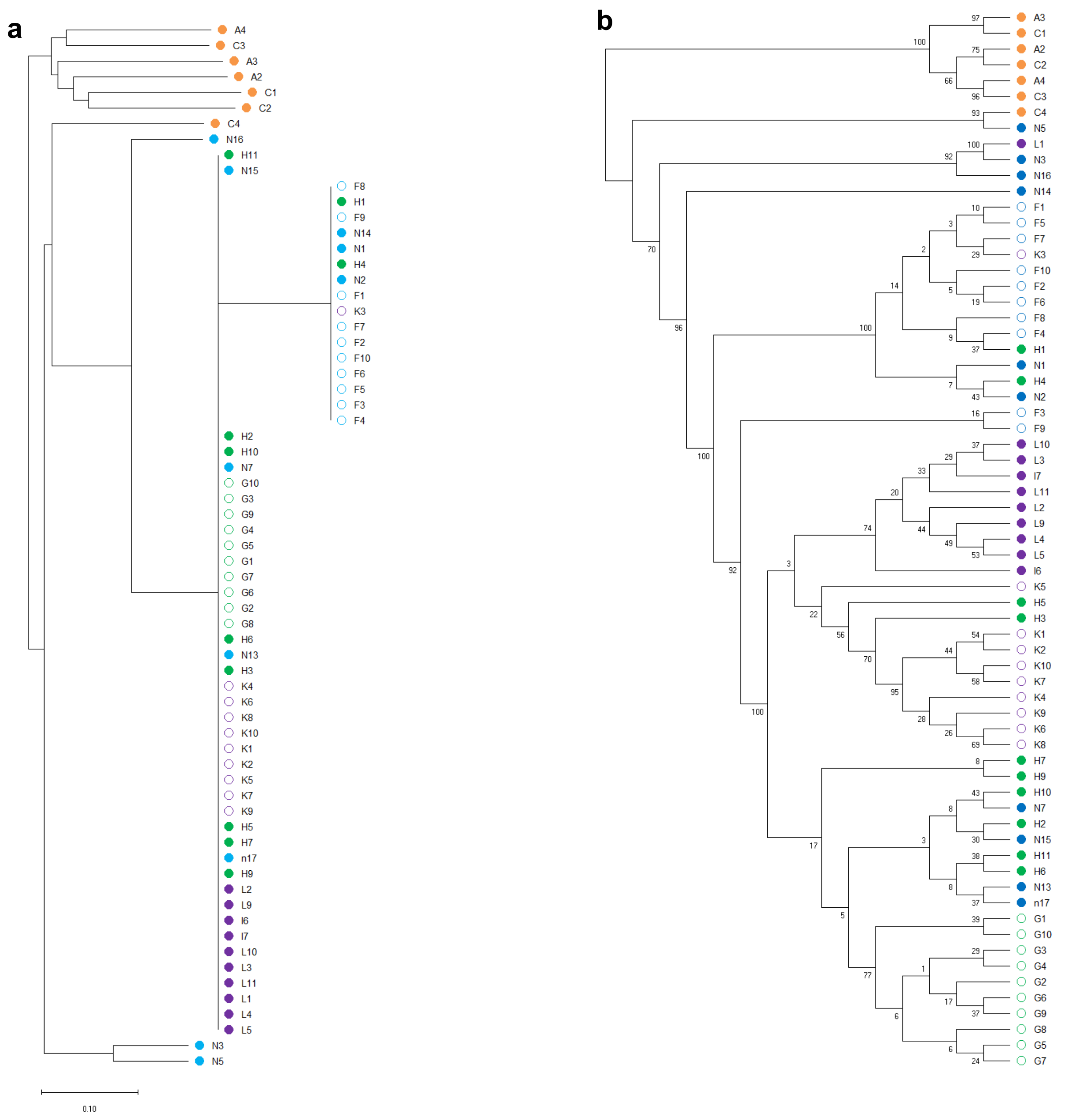

Supplementary Fig. 19 | Maximum-likelihood trees for the putative hotspot of genomic adaptation, flatwing-associated scaffold 18404 containing *dsx.* **a**, Tree drawn to scale, with bar representing the number of nucleotide substitutions per SNP site. (LnL=-301644.320) **b**, Consensus tree showing bootstrap values obtained from 1000 replicates. The scaffold spanned 1.3 Mb and contained 37,324 SNPs. Colour and symbol scheme follow that in the Main Text and population codes follow Supplementary Table 3: orange = *T. oceanicus* males from Australia, purple = Kauai males, blue = Oahu males, green = Hilo males. Solid circles represent normal-wing males, open circles represent flatwing males.

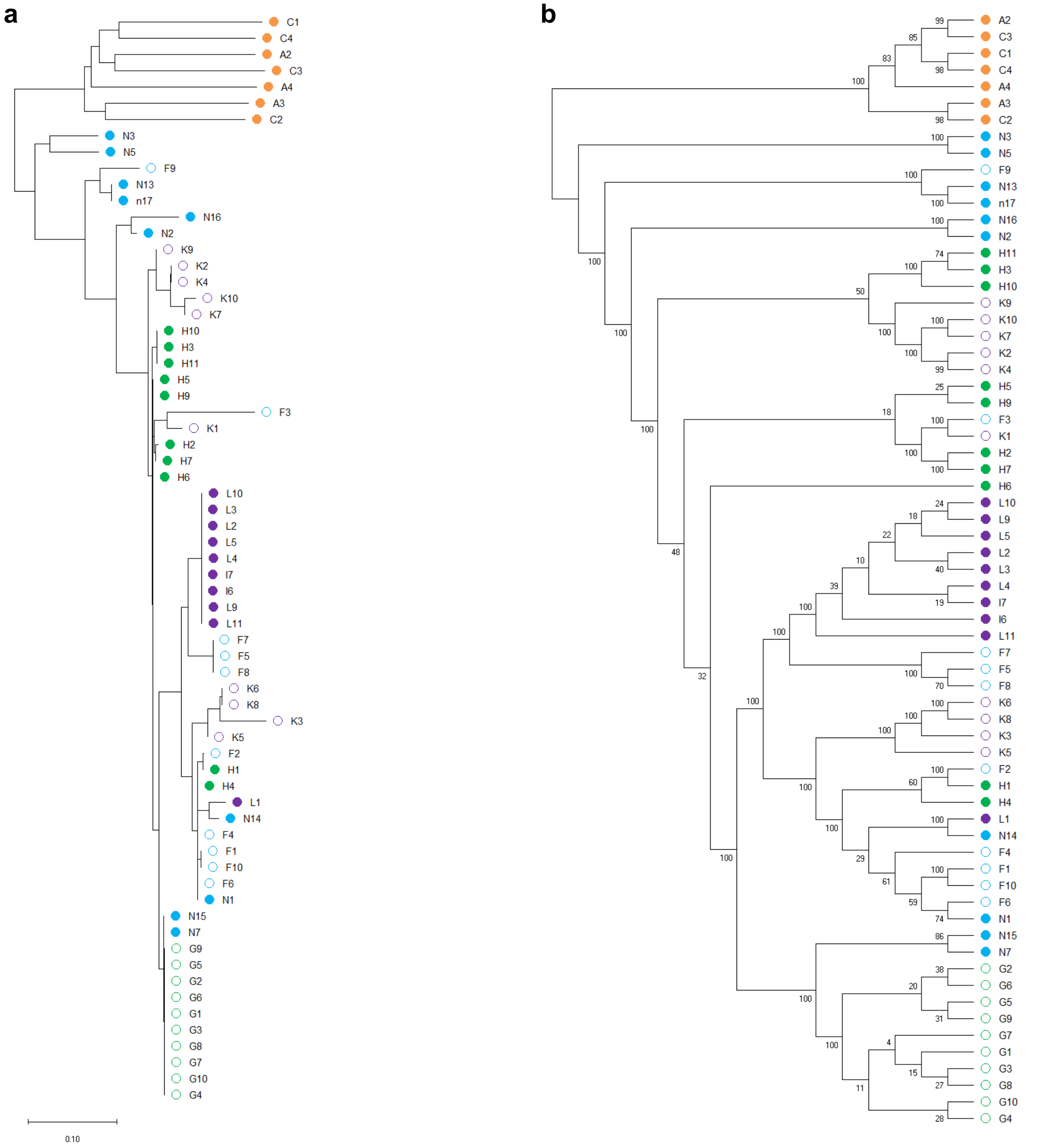

Supplementary Fig. 20 | Maximum-likelihood trees for the flanking regions (± 5 kb) of all flatwing-associated SNPs. a, Tree drawn to scale, with bar representing the number of nucleotide substitutions per SNP site. (LnL=-778920.574) **b**, Consensus tree showing bootstrap values obtained from 1000 replicates. Colour and symbol scheme follow that in the Main Text and population codes follow Supplementary Table 3: orange = *T. oceanicus* males from Australia, purple = Kauai males, blue = Oahu males, green = Hilo males. Solid circles represent normal-wing males, open circles represent flatwing males.

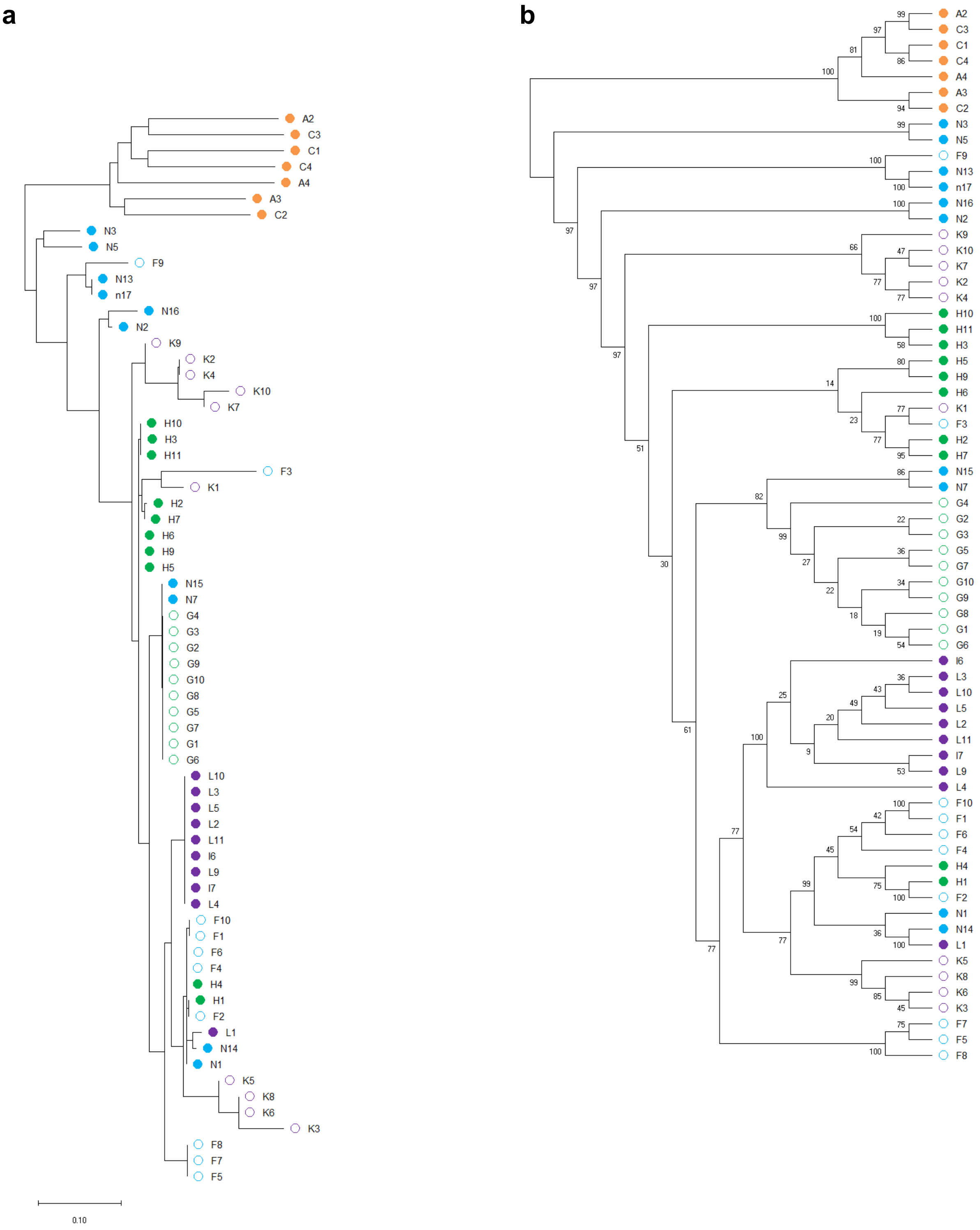

Supplementary Fig. 21 | Maximum-likelihood trees for the flanking regions (± 1 kb) of all flatwing-associated SNPs. a, Tree drawn to scale, with bar representing the number of nucleotide substitutions per SNP site. (LnL=-294180.850) **b**, Consensus tree showing bootstrap values obtained from 1000 replicates. Colour and symbol scheme follow that in the Main Text and population codes follow Supplementary Table 3: orange = *T. oceanicus* males from Australia, purple = Kauai males, blue = Oahu males, green = Hilo males. Solid circles represent normal-wing males, open circles represent flatwing males.

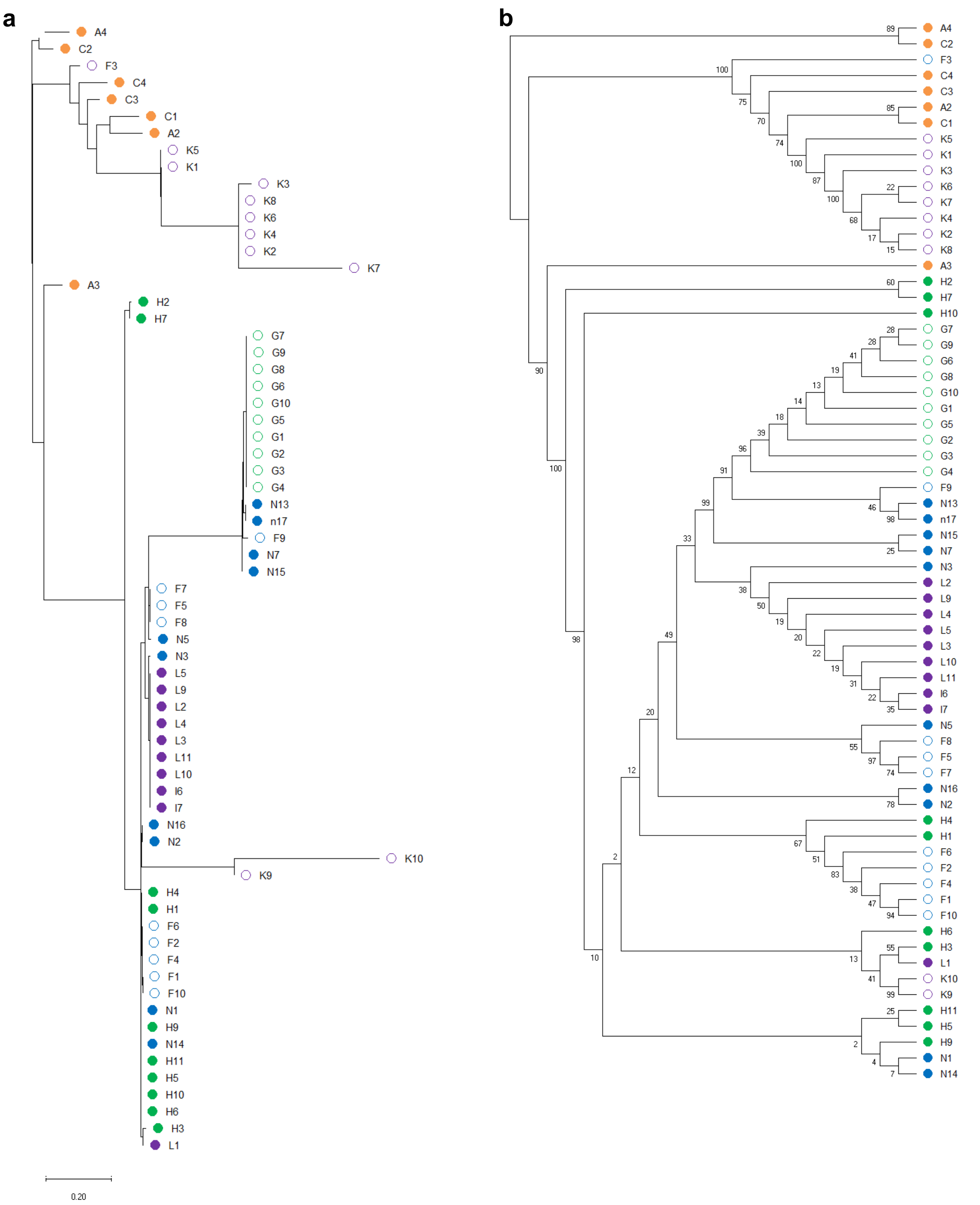

Supplementary Fig. 22 | Maximum-likelihood trees for all flatwing-associated SNPs. a, Tree drawn to scale, with bar representing the number of nucleotide substitutions per SNP site (LnL=-15309.796). **b**, Consensus tree showing bootstrap values obtained from 1000 replicates. Colour and symbol scheme follow that in the Main Text and population codes follow Supplementary Table 3: orange = *T. oceanicus* males from Australia, purple = Kauai males, blue = Oahu males, green = Hilo males. Solid circles represent normal-wing males, open circles represent flatwing males.

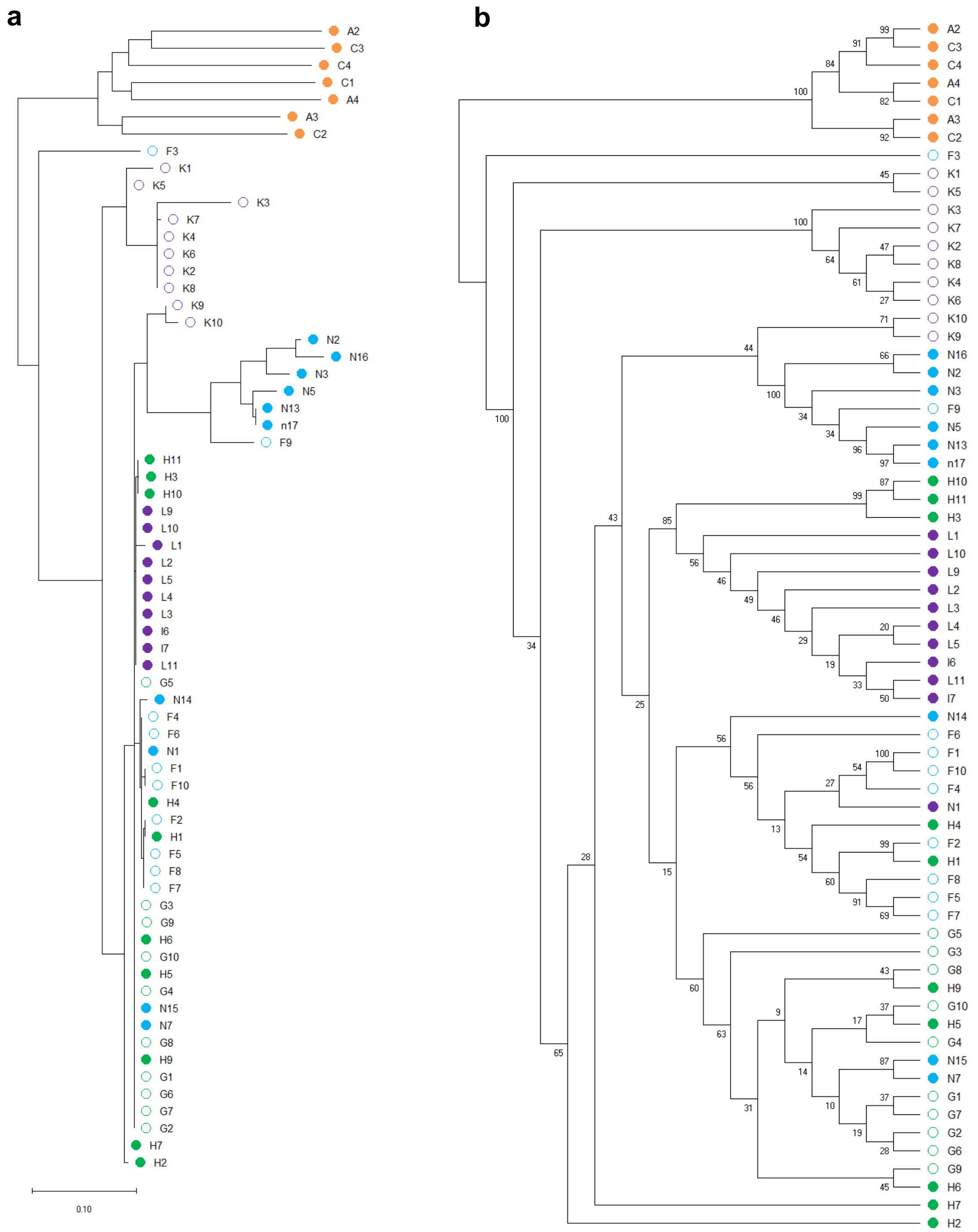

Supplementary Fig. 23 | Maximum-likelihood trees for the flanking regions (± 1 kb) of Kauai-specific flatwing-associated SNPs. a, Tree drawn to scale, with bar representing the number of nucleotide substitutions per SNP site. (LnL=-203432.575) **b**, Consensus tree showing bootstrap values obtained from 1000 replicates. Colour and symbol scheme follow that in the Main Text and population codes follow Supplementary Table 3: orange = *T. oceanicus* males from Australia, purple = Kauai males, blue = Oahu males, green = Hilo males. Solid circles represent normal-wing males, open circles represent flatwing males.

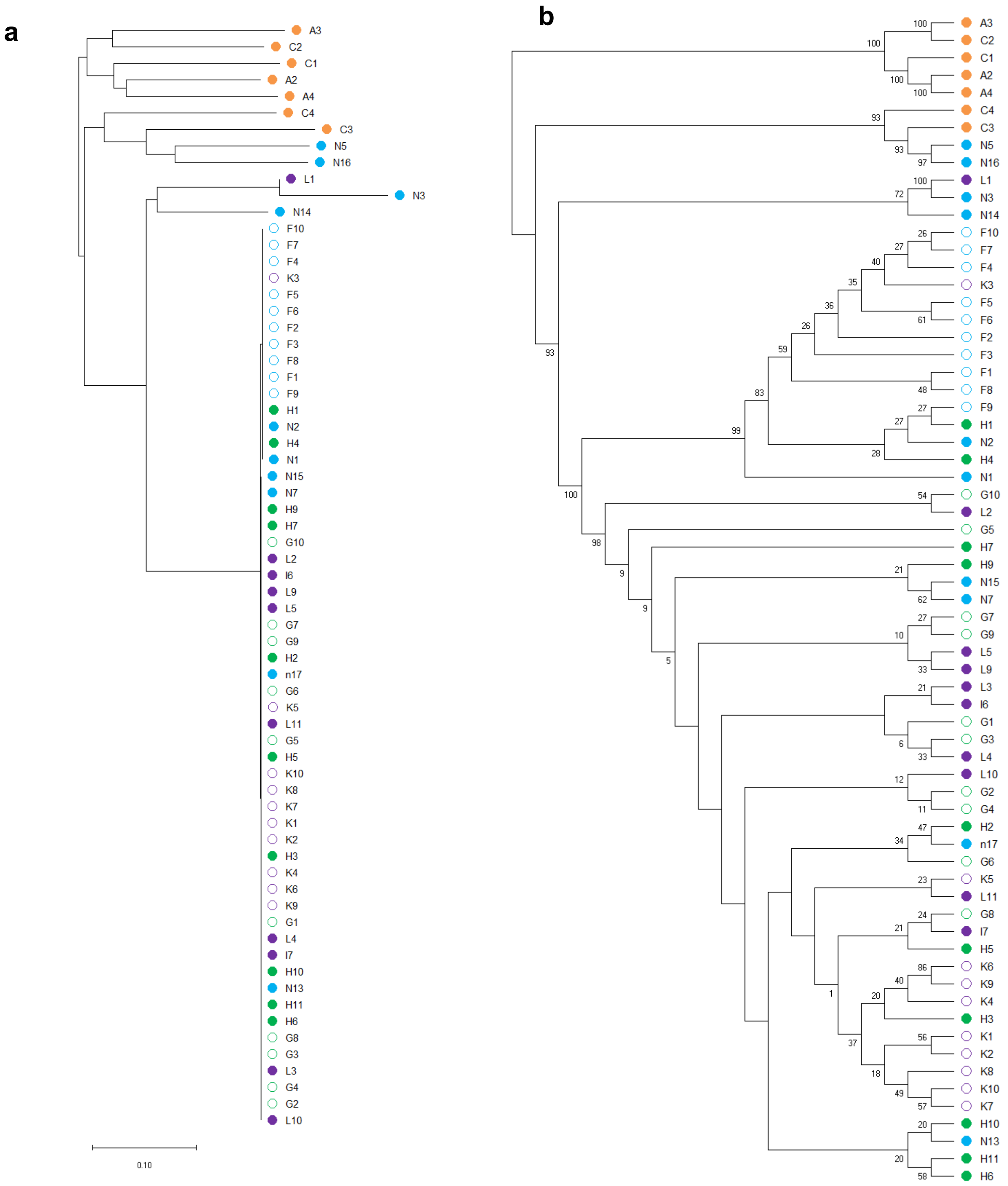

Supplementary Fig. 24 | Maximum-likelihood trees for the flanking regions (± 50 kb) of Oahu-specific flatwing-associated SNPs. a, Tree drawn to scale, with bar representing the number of nucleotide substitutions per SNP site. (LnL=-219265.002) **b**, Consensus tree showing bootstrap values obtained from 1000 replicates. Colour and symbol scheme follow that in the Main Text and population codes follow Supplementary Table 3: orange = *T. oceanicus* males from Australia, purple = Kauai males, blue = Oahu males, green = Hilo males. Solid circles represent normal-wing males, open circles represent flatwing males.

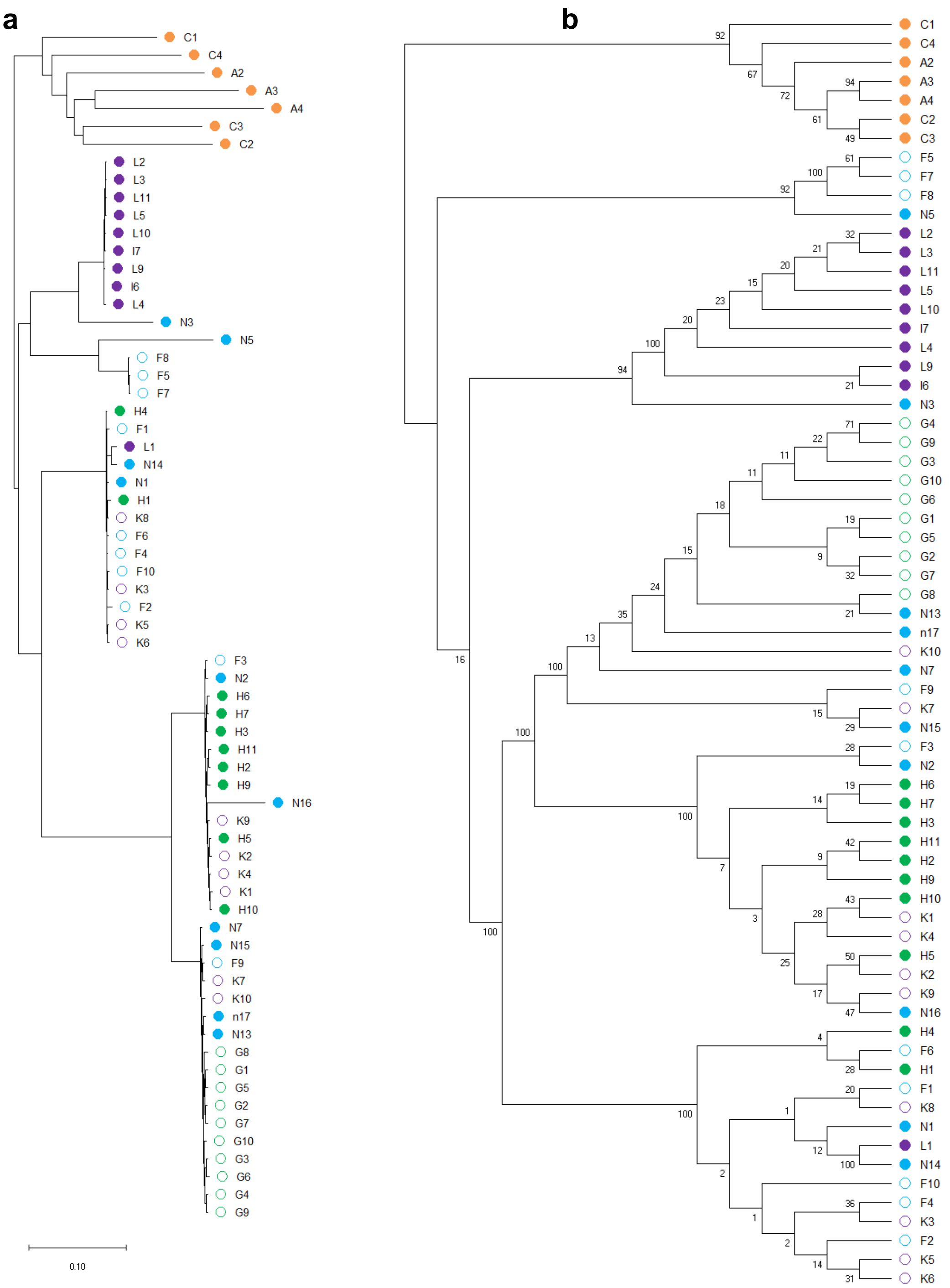

Supplementary Fig. 25 | Maximum-likelihood trees for the flanking regions (± 5 kb) of Hilo-specific flatwing-associated SNPs. a, Tree drawn to scale, with bar representing the number of nucleotide substitutions per SNP site. (LnL=-140680.988) **b**, Consensus tree showing bootstrap values obtained from 1000 replicates. Colour and symbol scheme follow that in the Main Text and population codes follow Supplementary Table 3: orange = *T. oceanicus* males from Australia, purple = Kauai males, blue = Oahu males, green = Hilo males. Solid circles represent normal-wing males, open circles represent flatwing males.

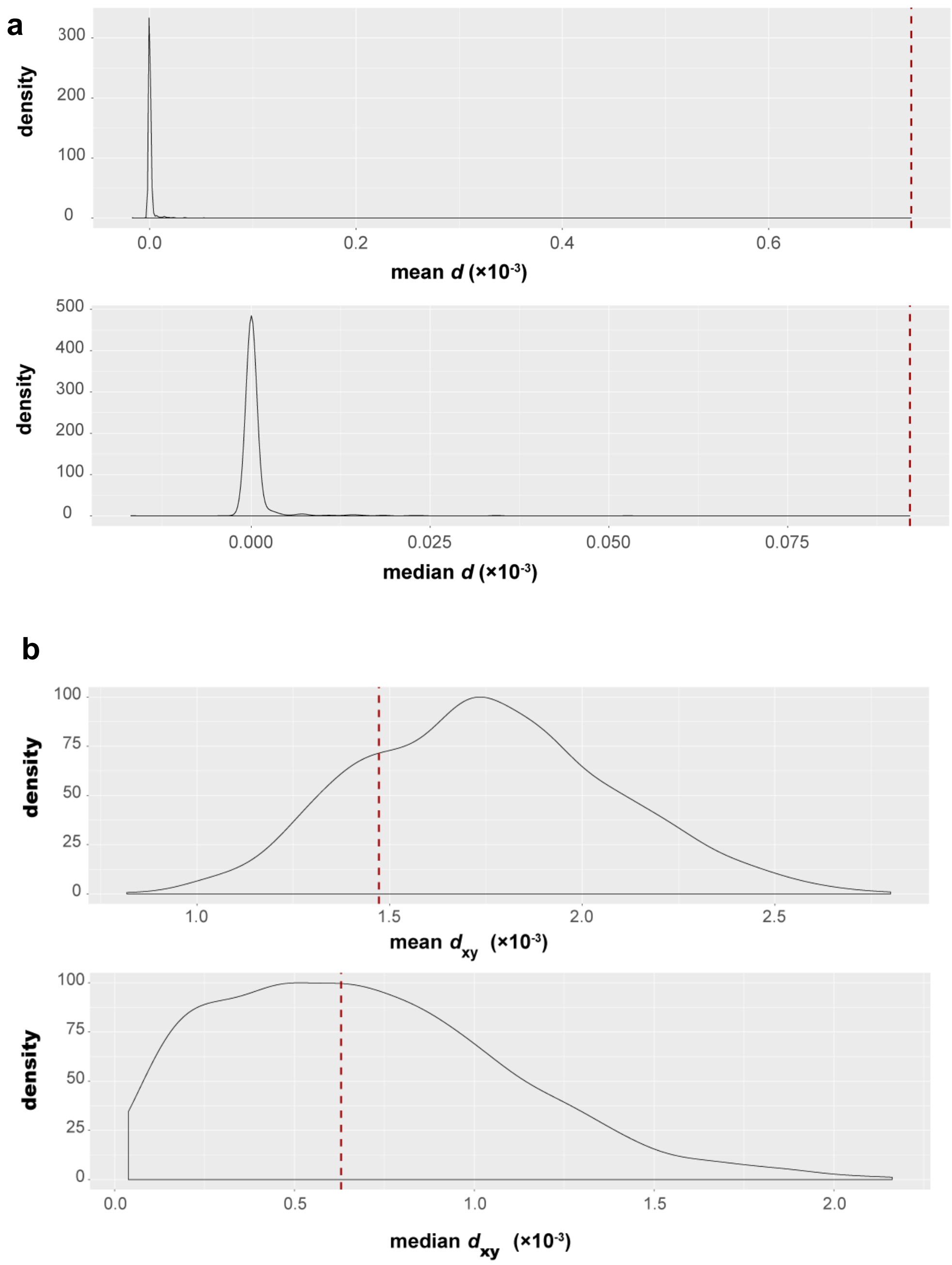

Supplementary Fig. 26 | Distributions of 1000 random simulations of net genetic distance *d* and absolute divergence d_{xy}. a, The mean (top) and median (bottom) genetic distance *d* across all X-chromosome markers. Observed values for flatwing-associated regions are shown as vertical dashed lines. Both a *t* test and simulations supported the observation that flatwing-associated regions showed significantly larger net nucleotide divergence (*d*) than the other X chromosomal regions (two-sided *t* test, $t = -8.1707$, df = 436.61, $P = 3.343e-15$, n = 437 sliding windows within flatwing region, n = 66,211 sliding windows outside flatwing region on the chromosome X). Here, $d = d_{xy} - (d_x+d_y)/2$. **b,** Flatwing-associated differences in absolute divergence (d_{xy}) were not driven by differences in genetic diversity. Panels show the mean (top) and median (bottom) d_{xy} across all X-chromosome markers. Observed values for flatwing-associated regions are shown as vertical dashed lines.

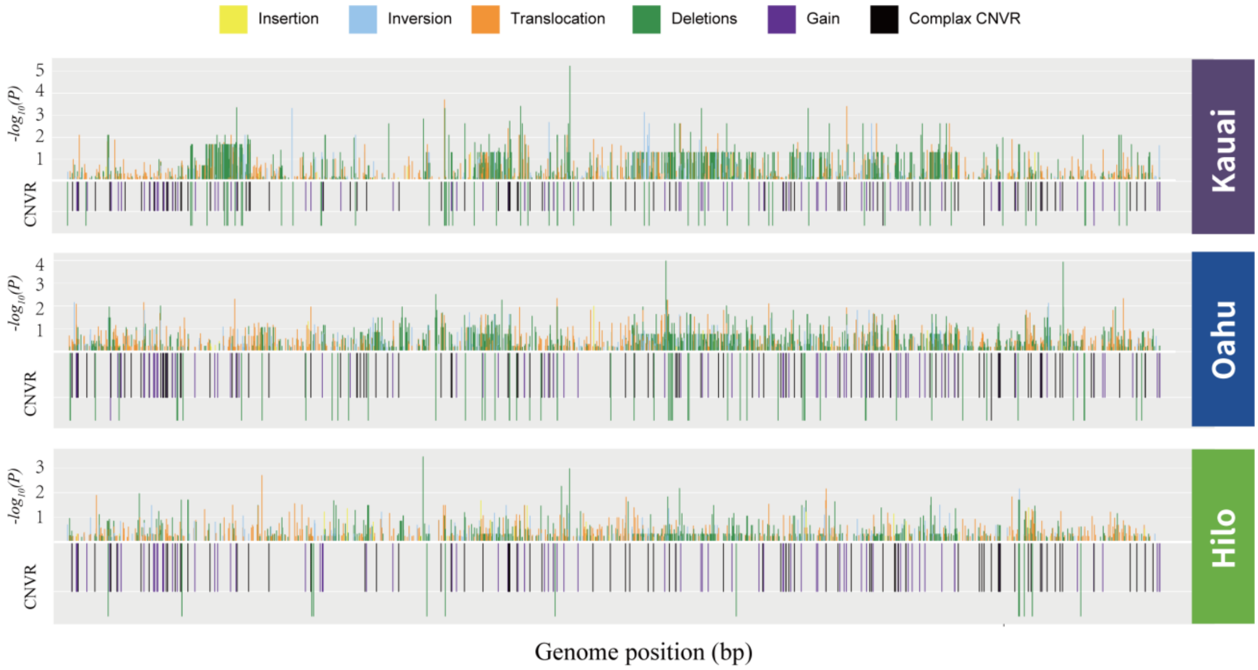

Supplementary Fig. 27 | X-chromosome landscape of structural variants and their association with flatwing phenotypes in different populations. Structural variants detected using breakdancer are illustrated above copy number variation regions (CNVRs). Different colours represent different types of structural variant (SV) and CNVR. Bar height represents association significance. For CNVRs, only two different heights are presented, with phenotype-differentiated CNVRs represented by longer bars. Insignificant deletion-type CNVRs are not shown.

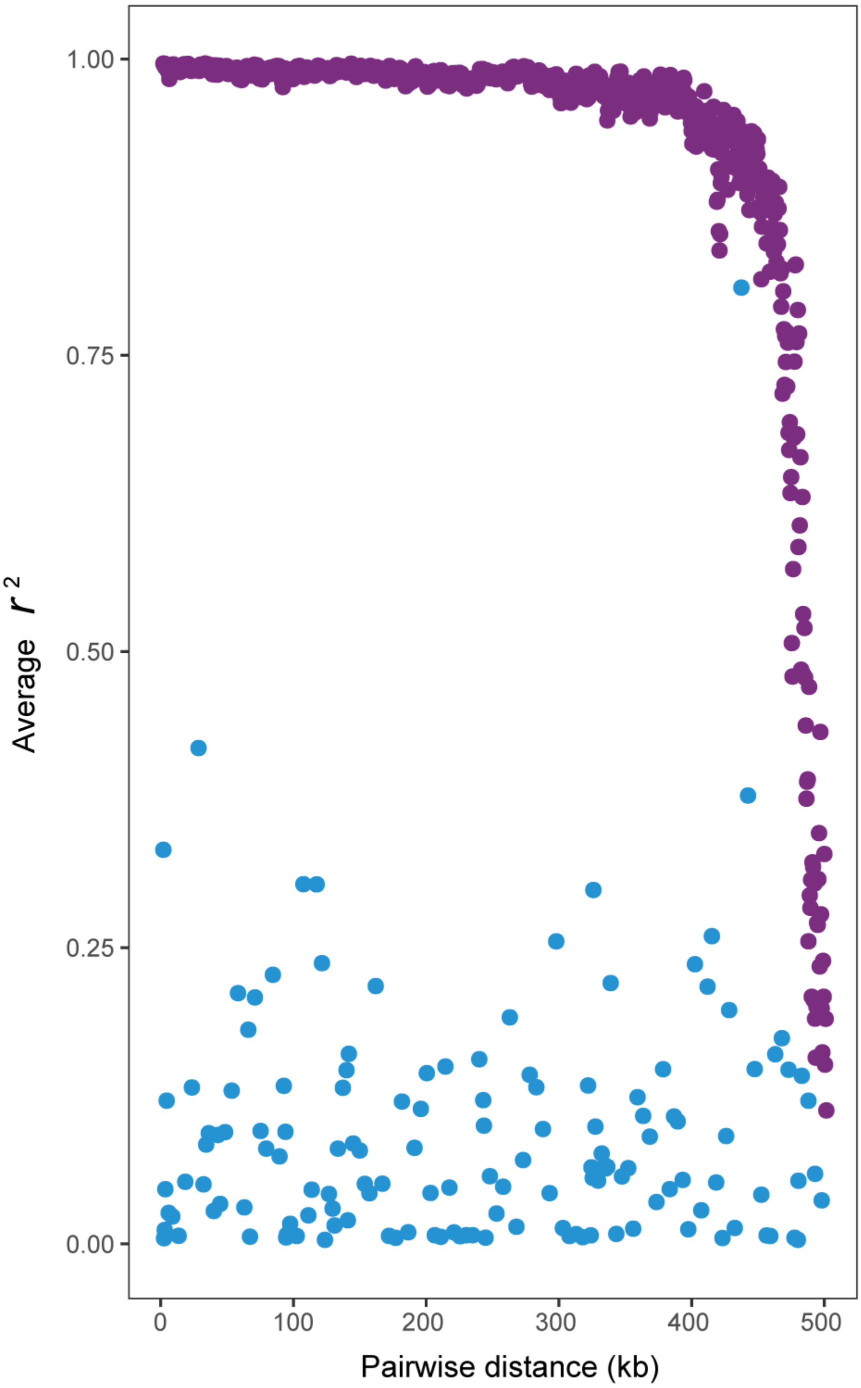

Supplementary Fig. 28 | Scaffold 18404-wide patterns of decay of linkage disequilibrium for Kauai (purple) and Oahu (blue) flatwing individuals. Dots represent the mean pairwise r^2 values between pairs of SNPs.

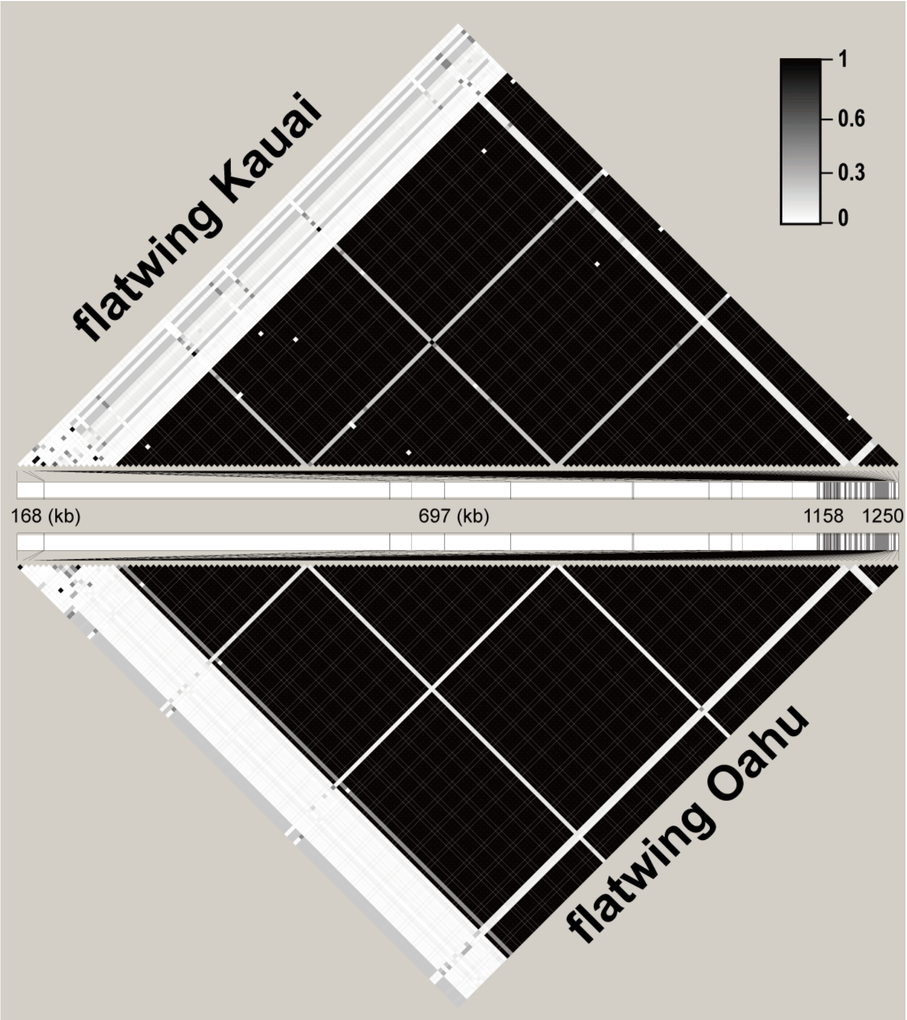

Supplementary Fig. 29 | Different extents of linkage distribution (LD) along the flatwing-associated scaffold "6636". Top and bottom heatmaps show pairwise LD (r^2) between SNPs from Kauai and Oahu, respectively. The colour scale refers to the value of r^2.

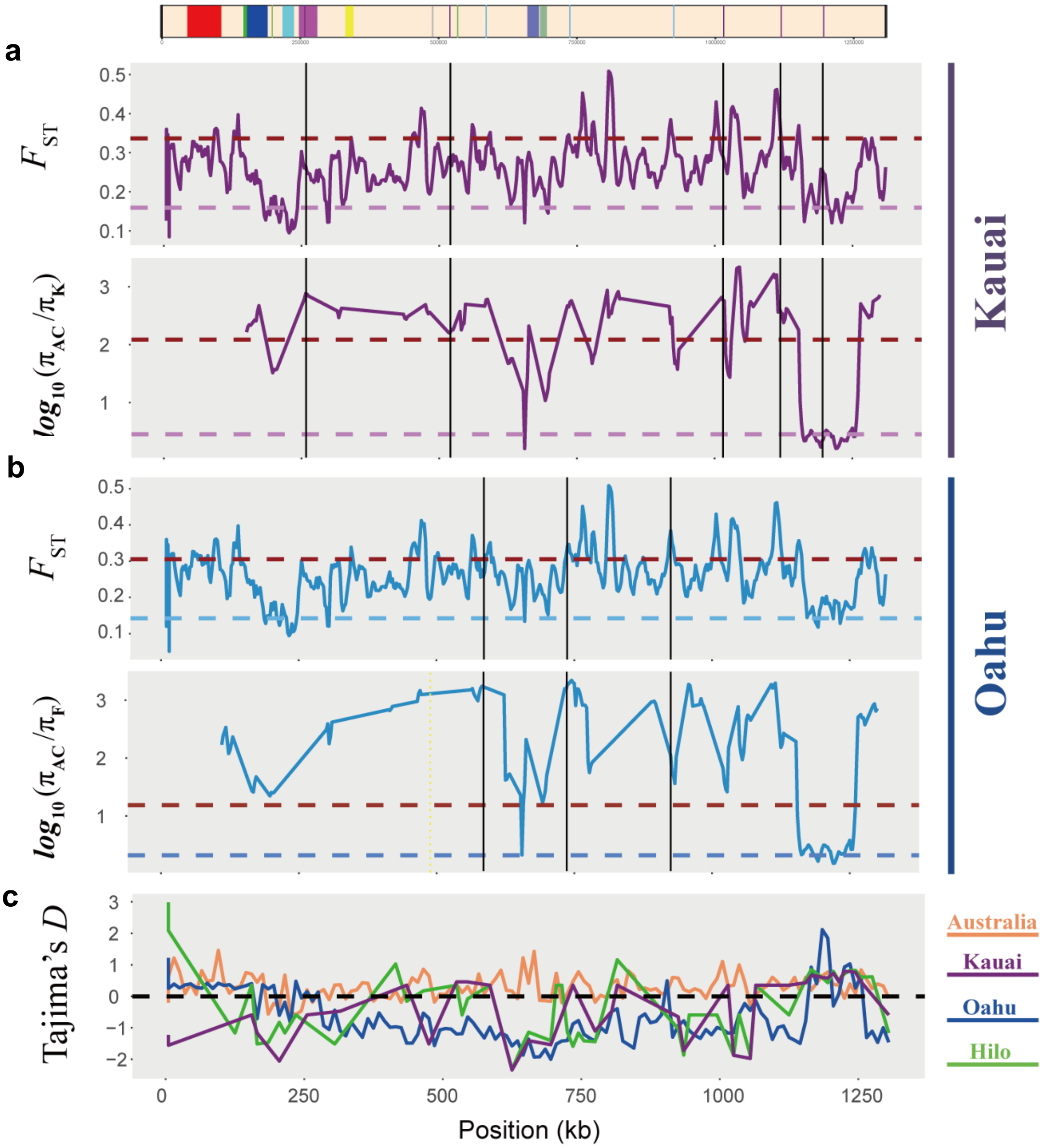

Supplementary Fig. 30 | Selection on flatwing-associated "scaffold 6636" in Hawaiian *T. oceanicus*. Genes and flatwing-associated SNPs are shown in the top panel using coloured blocks and bars respectively. F_{ST} and nucleotide diversity (π) were calculated using flatwing males from the two populations in which wild-caught samples were available (**a,** Kauai and **b,** Oahu). $\pi_{ratio} = \pi_{selected\_flatwing}/\pi_{unselected\_normal\text{-}wing}$. Horizontal dashed lines represent mean whole-genome value for the values shown in each panel, and dark red horizontal dashed lines in a and b represent top 5% threshold values. **c,** Tajima's D was calculated for wild samples using a sliding-window analysis with 10 kb windows only for groups where wild-caught samples were available. The dashed horizontal line indicates the Tajima's D null hypothesis for neutrality.

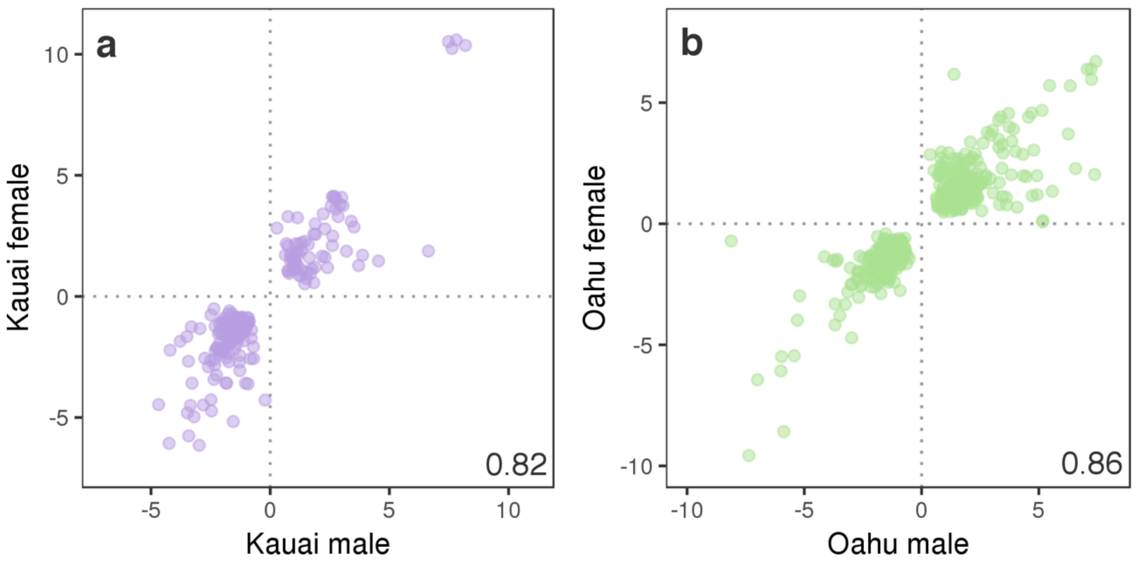

Supplementary Fig. 31 | Correlated changes in *flatwing*-associated gene expression between sexes in each population. a, Kauai (purple)**,** and **b,** Oahu (green). Values in the bottom-right of each plot are Spearman's rank correlation coefficients, and log_2-fold changes >0 indicate normal-wing biased expression.

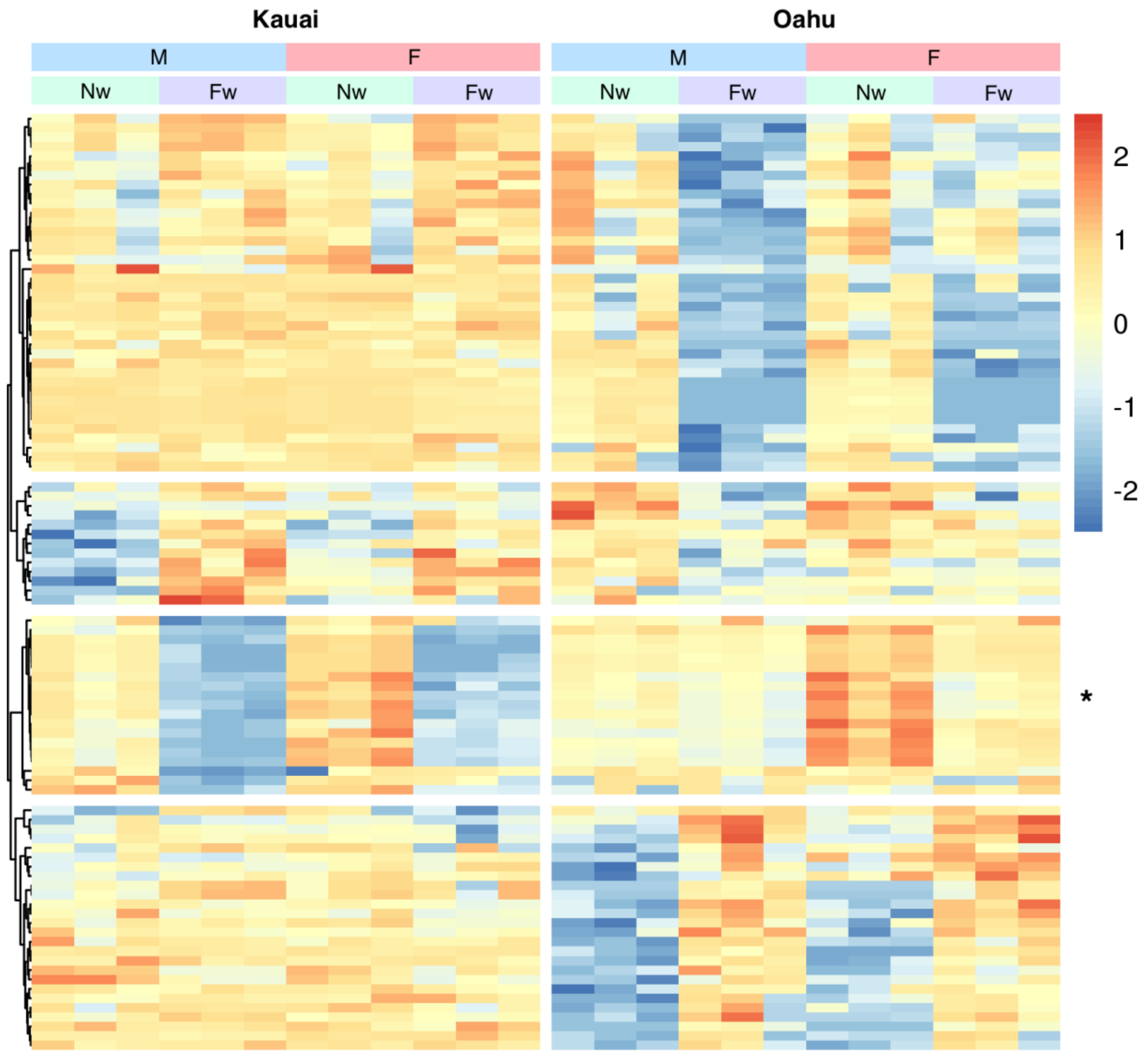

Supplementary Fig. 32 | Correlated changes in *flatwing*-associated gene expression between sexes in each population. Relative expression values, scaled by row (i.e. transcript), for all transcripts reported as DE between genotypes in either population. The colour scale refers to relative expression values. Transcripts are clustered by co-expression patterns, with four levels specified following visual inspection. The asterisk indicates the cluster of transcripts showing correlated patterns of differential expression between islands, and containing both DE transcripts from scaffold 18404, including *doublesex*.

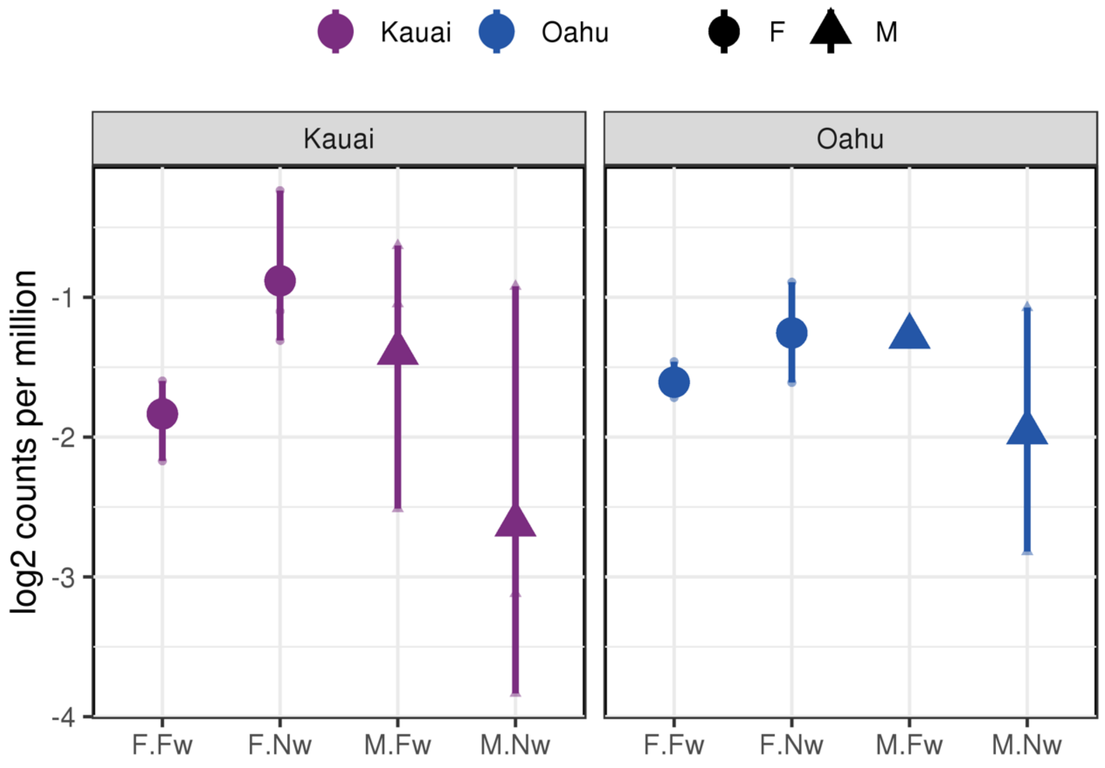

Supplementary Fig. 33. Expression of *prospero* across sexes and genotypes of each island. Large points show means, small points show individual observations for each replicate, and lines indicate minimum and maximum values. No genotype comparison within sexes yielded any differences approaching significance (all FDR > 0.79).

Supplementary Tables

Supplementary Table 1 | Sampled sites, years, and sample sizes for geometric morphometric analysis of flatwing male forewings.

Population (code)	Sample size			
	2016	2017	2018	Total
Kauai (K)	16	19	17	52
Oahu (F)	16	19	17	52
Hilo (G)	4	4	2	10

Supplementary Table 2 | Statistical analysis of flatwing male forewing venation using multivariate analysis of variance (MANOVA) on principal component scores from landmark analysis.

**Model[1,2]**	**df (island)**	**df (residuals)**	**Pillai**	**approx F**	**num df**	**den df**	***P***
(PC1,PC2)~island	2	111	0.182	5.57	4	222	2.728e-04
(PC1,PC2,PC3)~island	2	111	0.273	5.80	6	220	1.251e-05
(all 28 PCs)~island	2	111	1.358	6.42	56	170	2.2e-16

[1] Three models were run, to examine significance of PCs 1 & 2, PCs 1 - 3, and all 28 PCs.

[2] PCs 1 – 3 accounted for 46.0%, 14.9%, and 11.5% of variance in the principal component analysis, respectively.

Supplementary Table 3 | Population codes, sample locations, male morph, sample sizes, sampling date, and colour-coding scheme for whole genome resequenced *Teleogryllus* males.

Population ID[1]	Sampling location	Morph	Number of individuals	Sampling date
K	Wailua, Kauai	Flatwing	10	2017
L	Wailua, Kauai[3]	Normal-wing	10	2017
F	Manoa, Oahu	Flatwing	10	2017
N	Manoa, Oahu	Normal-wing	10	2017
G	Church property, Hilo[3]	Flatwing	10	2017
H	Church property, Hilo	Normal-wing	10	2017
A	Cairns, Australia	Normal-wing	3	2011
C	Mission Beach, Australia	Normal-wing	4	2011
B[2]	Bluey's Beach, Australia	Normal-wing	3	2011
	Total		70	

[1] All populations except "B" are *T.oceanicus.*

[2] This outgroup population was the closely-related sister species, *T. commodus*.

[3] Laboratory stocks established from wild-caught ancestors.

Supplementary Table 4 | Summary of statistical metrics for Patterson's *D* comparisons between all Hawaiian scenarios and Australian scenarios using LG1 (the X chromosome), corresponding to Fig. 2a.

Type (P3) [1,2]	Mean |*D*|		Statistic metrics [3]		
	Group 1	Group 2 (control)	*P* value	t	df
Kauai vs Australian control 1	0.534	0.173	1.74E-217	36.767	1675.4
Kauai vs Australian control 2		0.151	2.33E-243	39.807	1647.6
Kauai vs Australian control 3		0.167	2.16E-241	40.085	1543.4
Oahu vs Australian control 1	0.400	0.173	2.07E-159	30.070	1686.2
Oahu vs Australian control 2		0.151	1.09E-197	34.158	1805.4
Oahu vs Australian control 3		0.167	2.84E-212	34.966	2140.0
Hilo vs Australian control 1	0.436	0.173	8.13E-151	29.033	1697.9
Hilo vs Australian control 2		0.151	6.85E-178	32.205	1692.5
Hilo vs Australian control 3		0.167	8.62E-177	32.250	1624.7

[1] Source data are available in Supplementary Data 3. Source data are also provided as a Source Data file.

[2] Sample sizes n = 700 independent *D* values for each Australian control; n = 1,000 each for Kauai and Hilo; n = 2,000 for Oahu.

[3] Two-sided *t* test

Supplementary Table 5 | Summary of D statistics for all population comparisons in ABBA-BABA2 tests.

D	Z	*P* value[3]	nABBA	nBABA	nBlocks	Pop. 1	Pop. 2	Pop. 3	Pop. 4
0.097945	22.54925	<0.001	62089.21	51011.52	1082	Hilo	Kauai	AC[1]	OUT[2]
0.158079	23.90454	<0.001	90452.44	65758.68	1082	Hilo	Oahu	AC	OUT
-0.08104	-17.0279	<0.001	77176.31	90787.41	1082	Oahu	Kauai	AC	OUT
0.41356	50.91003	<0.001	193521.4	80285.81	1082	Oahu	Hilo	Kauai	OUT
-0.13184	-15.5623	<0.001	80285.81	104669.6	1082	Hilo	Kauai	Oahu	OUT
0.29797	27.70577	<0.001	193521.4	104669.6	1082	Oahu	Kauai	Hilo	OUT

[1] Data from all Australian *T. oceanicus* samples combined across populations

[2] Outgroup was Australian *T. commodus* samples

[3] Significance was assessed using the block jackknife method implemented in ANGSD. *D* value was considered to be significantly different from 0 when *P* value < 0.001, corresponding to the two-tailed significance threshold of |z| > 3.

Supplementary Table 6 | Summary of candidate flatwing-associated SNPs for each Hawaiian population.

Threshold	Population	Chromosome X		Unplaced		Total	
		Candidate SNPs	Candidate scaffolds	Candidate SNPs	Candidate scaffolds	Candidate SNPs	Candidate scaffolds
Oahu criterion[1]	Kauai	7	4	9	4	16	8
	Oahu	5	3	0	0	5	3
	Hilo	177	9	102	24	279	33
Adjusted criterion[2]	Kauai	615	17	373	27	988	44
	Oahu	5	3	0	0	5	3
	Hilo	201	10	163	39	364	49

[1] Top 0.0034% of genome-wide SNPs showing association with flatwing morph.

[2] Bonferroni-adjusted *P* values <0.001 for both Hilo and Kauai populations, FDR-adjusted *P* values < 0.05 for the Oahu population. (Exact *P* values are reported in the Supplementary Data 5)

Supplementary Table 7 | Scaffold contents of Venn diagram in Main Text Fig. 3b.

Group	Number of scaffolds	Scaffold names
Hilo-Kauai-Oahu	2	Contig6636_pilon Contig18404_pilon
Hilo-Oahu	1	Contig2899_pilon
Hilo	6	Contig3077_pilon Contig28042_pilon Contig7882_pilon Contig31669_pilon Contig30320_pilon Contig31374_pilon
Kauai	2	Contig4959_pilon Contig26934_pilon

Supplementary Table 8 | Summary of structural variants (SVs) and copy number variation regions (CNVRs) detected in Hawaiian *T. oceanicus* populations.

		Kauai	Oahu	Hilo
CNVR	deletion	5638	5855	5561
	duplication	99	103	95
	combination	100	120	99
SV	deletion	2309	2571	2091
	translocation	1272	1293	1191
	inversion	1144	1133	1108
	insertion	71	72	68

Supplementary Table 9 | Summary of putative genomic regions under selection in Hawaiian populations of *T. oceanicus*.

Population	Threshold		% of all windows	Number of selective-sweep regions	Total region size (Mb)	% of genome	Overlapped GWAS candidate SNP
	log (π ratio)	F_{ST}					
**Kauai**	2.08	0.34	0.61	1230	17.05	0.83	12
**Oahu**	1.19	0.31	1.17	1691	28.03	1.37	3
**Hilo**	2.40	0.38	0.31	697	9.04	0.44	N/A[1]

[1] All wild Hilo crickets that were resequenced were normal-wing individuals. Thus, it was not possible to evaluate overlap between selective sweep regions and flatwing- associated SNPs.

Supplementary Table 10 | GWAS for the Oahu flawing population performed using the same strategy as for Kauai (10 wild Oahu flatwing vs. all normal-wing) is qualitatively identical to the original results.

Coordinates		Minor allele	Frequency in cases	Frequency in controls	Major allele	*P* value [1]	Adjusted *P* [2]	-log *P* [3]
Contig18404_pilon	930669	A	0.9	0	G	5.35E-16	2.49E-08	15.27
Contig2899_pilon	2218526	C	0.9	0	A	5.35E-16	2.49E-08	15.27
Contig6636_pilon	736911	C	1	0.06897	T	5.37E-15	2.50E-07	14.27
Contig6636_pilon	585940	A	1	0.1	G	6.51E-14	3.03E-06	13.19
Contig6636_pilon	924960	G	1	0.1	A	6.51E-14	3.03E-06	13.19

[1] *P* values of two-sided Fisher's Exact tests implemented in PLINK

[2] Adjusted *P* values for multiple comparisons

[3] $-\log_{10}$ (*P* value)
