# Supplementary material for: Rapid parallel adaptation despite gene flow in silent crickets
Source: Nat Commun. 2021 Jan 4;12:50. doi: 10.1038/s41467-020-20263-4 (PMC7782688; doi:10.1038/s41467-020-20263-4)
Supplement: Supplementary file 3 — Reporting summary [file 41467_2020_20263_MOESM3_ESM.pdf]

nature research

Corresponding author(s): Nathan W. Bailey, Xiao Zhang

Last updated by author(s): Nov 19, 2020

Reporting Summary

Nature Research wishes to improve the reproducibility of the work that we publish. This form provides structure for consistency and transparency in reporting. For further information on Nature Research policies, see our Editorial Policies and the Editorial Policy Checklist.

Statistics

For all statistical analyses, confirm that the following items are present in the figure legend, table legend, main text, or Methods section.

n/a	Confirmed	
☐	☒	The exact sample size (*n*) for each experimental group/condition, given as a discrete number and unit of measurement
☐	☒	A statement on whether measurements were taken from distinct samples or whether the same sample was measured repeatedly
☐	☒	The statistical test(s) used AND whether they are one- or two-sided<br>*Only common tests should be described solely by name; describe more complex techniques in the Methods section.*
☒	☐	A description of all covariates tested
☐	☒	A description of any assumptions or corrections, such as tests of normality and adjustment for multiple comparisons
☐	☒	A full description of the statistical parameters including central tendency (e.g. means) or other basic estimates (e.g. regression coefficient) AND variation (e.g. standard deviation) or associated estimates of uncertainty (e.g. confidence intervals)
☐	☒	For null hypothesis testing, the test statistic (e.g. F, t, r) with confidence intervals, effect sizes, degrees of freedom and P value noted<br>*Give P values as exact values whenever suitable.*
☒	☐	For Bayesian analysis, information on the choice of priors and Markov chain Monte Carlo settings
☐	☒	For hierarchical and complex designs, identification of the appropriate level for tests and full reporting of outcomes
☐	☒	Estimates of effect sizes (e.g. Cohen's *d*, Pearson's *r*), indicating how they were calculated

Our web collection on statistics for biologists contains articles on many of the points above.

Software and code

Policy information about availability of computer code

Data collection	Morphometric landmark data were generated by manually placing 16 landmarks on wing photographs using tpsDig2 (v2.31). Whole genome re-sequencing data were generated using the Illumina HiSeq X platform at Edinburgh Genomics. RNA-seq data were generated using the Illumina HiSeq 4000 platform at the Centre for Genomic Research (University of Liverpool).
Data analysis	We used QGIS (v2.18.24), tpsDig2 (v2.31), MorphoJ (v1.07a), bcl2fastq (v2.20), BWA-MEM (v0.7.12), samtools (v0.1.19, v1.3.1), picard (v2.14.1), GATK (v3.7.0), PLINK (v1.90b6.8), PHYLIP (v3.696), MEGA (v10.0.5), FigTree (v1.4.4), EIGENSOFT (v7.2.1), ADMIXTURE (v1.3.0), grl (v0.100.30), ggplot2 (v3.1.0), R (v3), ADMIXTURE (v1.3.0), VCFtools (v0.1.16), Haploview (v4.2), ANGSD (v0.930), PGDSpider (v2.1.1.5), Fastsimcoal (v2.6.0.3), Arlequin (v3.5), PSMC (v0.6.5-r67), Breakdancer (v1.3.6), CNVnator (v0.3.3), PopGenome (v2.6.1), CASAVA (v1.8.2), Cutadapt (v1.2.1), Sickle (v1.200), HiSat2 (v2.1.0), StringTie (v1.3.4), edgeR (v3.20.9) in this study (see Methods). Customized Perl and R scripts are available on ChirpBase (http://download.chirpbase.org/v2/scripts/Rapid_parallel_adaption_despite_gene_flow/).

For manuscripts utilizing custom algorithms or software that are central to the research but not yet described in published literature, software must be made available to editors and reviewers. We strongly encourage code deposition in a community repository (e.g. GitHub). See the Nature Research guidelines for submitting code & software for further information.

Data

Policy information about availability of data

All manuscripts must include a data availability statement. This statement should provide the following information, where applicable:

- Accession codes, unique identifiers, or web links for publicly available datasets
- A list of figures that have associated raw data
- A description of any restrictions on data availability

Raw resequencing reads have been deposited in the European Nucleotide Archive with the accession number PRJEB39125. Raw RNA-seq reads have been deposited

in the same archive (accession number PRJEB40088). Raw morphometric data are publicly available on ChirpBase (http://download.chirpbase.org/v2/morphology/).

Field-specific reporting

Please select the one below that is the best fit for your research. If you are not sure, read the appropriate sections before making your selection.

☐ Life sciences ☐ Behavioural & social sciences ☒ Ecological, evolutionary & environmental sciences

For a reference copy of the document with all sections, see nature.com/documents/nr-reporting-summary-flat.pdf

Ecological, evolutionary & environmental sciences study design

All studies must disclose on these points even when the disclosure is negative.

Study description	We performed geometric morphometric comparisons of wings of crickets (total n = 114 experimental observations from three different populations, as described in the Methods section. Principal components analysis (PCA) was performed on geometric morphometric landmark data, then multivariate analysis of variance was performed on PCA scores with 'population' as a fixed effect and independent responses of all 28 principal components (PCs), PCs 1 & 2, and PCs 1-3. Canonical variates analysis was performed on scores from all PCs with 'population' as a fixed effect). We performed whole genome re-sequencing (total n = 70 experimental units, with n = 10 per morph per population except for outgroups which had n = 3 for the sister species and n = 7 for the same species) and tested for gene flow among 3 populations and reconstructed population histories. We performed independent genome-wide association analyses on the same set of resequenced samples, and investigated candidate loci these revealed. We analysed signatures of genomic selection on the same set of resequenced samples, and performed gene expression analyses of developing cricket wings. Experimental replicates were not included in the study, with the exception of gene expression analyses which used three biological replicates per morph group.
Research sample	Population codes, sample locations, male morph, sample sizes, and sampling date for whole genome resequencing of individuals are described in Supplementary Table 3. All were adult (ca. 5-25 days post-eclosion). Sampling details for morphometric analyses are described in the Supplementary Table 1. The rationale for sample choice is that sampling was performed haphazardly within the respective populations or lines to ensure representative samples, and the sample size reflected trade-offs between sequencing cost and statistical power. We collected and sequenced 24 RNA samples of penultimate instar nymphs of normal-wing and flatwing genotypes of both sexes, from Kauai and Oahu lab populations (3 replicate lines per group). To help validate this, we compared the identity of transcripts DE between morphs from Kauai with those found to be DE between in previously published samples from Kauai male wingbuds. These previously published data are identified and cited (Pascoal, S. et al. 2016, ENA accession number PRJNA283744).
Sampling strategy	We collected right forewings of 114 wild flatwing male crickets sampled haphazardly to achieve a representative sample for each treatment group, by removing them and mounting them between slides, over multiple years from populations on the islands of Kauai, Oahu and Hilo. Sampling was similarly randomised, i.e. blind to any characteristics other than population/group identity, for resequenced whole genomes of 70 male crickets. We collected and sequenced 24 RNA samples from the wingbuds of normal-wing and flatwing genotypes of both sexes, from Kauai and Oahu lab populations. Sample sizes for resequencing and RNAseq reflected trade-offs between sequencing cost, read depth and statistical power.
Data collection	Samples for DNA extraction and morphometrics were collected haphazardly within each population to avoid biases arising from local relatedness structure. X.Z. and J.G.R. collected lab-reared samples randomly from existing lab populations originally established from the offspring of individuals in respective populations. Morphometric data were collected by X.Z. using a Leica dissecting microscope with a mounted digital camera, recording photographs to a desktop computer. Genome re-sequencing data and RNA-seq data were generated as described above. We included a comparison to previously published quantitative trait locus (QTL) data; this is identified in the text with the relevant citation (Pascoal, S. et al. 2019, ENA accession number PRJEB29921).
Timing and spatial scale	Timings and spatial scales of sampling for the study are described in Supplementary Tables 1 and 3 (spatial scale spanning ca 550 km in Hawaii and 8000 km across the Pacific, between 2011-2018).
Data exclusions	No data were excluded from the analyses.
Reproducibility	We performed mutiple independent analyses for each set of conclusions, e.g. analysing gene flow using two independent techniques (ABBA-BABA tests and Fastsimcoal), and e.g. inferring independent origins of adaptive mutations using GWAS and phylogenetic tree-based comparisons. We additionally made use of previously published datasets to independently validate the present findings. The combination of multiple lines of evidence supports our conclusion that the findings are reproducible. Experiments were not replicated.
Randomization	We did not apply experimental treatments in this study, so randomization was not implemented in this context. We made efforts to not bias sampling by relatedness structure within populations, and when laboratory samples were contrasted with in-situ samples from populations in nature, this was clearly identified and limitations discussed in the main text.
Blinding	All samples were numbered and subject to sequencing by staff members blind to our sampling strategy. Blinding was also used during analysis.

Did the study involve field work? ☐ Yes ☒ No

Reporting for specific materials, systems and methods

We require information from authors about some types of materials, experimental systems and methods used in many studies. Here, indicate whether each material, system or method listed is relevant to your study. If you are not sure if a list item applies to your research, read the appropriate section before selecting a response.

Materials & experimental systems

n/a	Involved in the study
☒	☐ Antibodies
☒	☐ Eukaryotic cell lines
☒	☐ Palaeontology and archaeology
☐	☒ Animals and other organisms
☒	☐ Human research participants
☒	☐ Clinical data
☒	☐ Dual use research of concern

Methods

n/a	Involved in the study
☒	☐ ChIP-seq
☒	☐ Flow cytometry
☒	☐ MRI-based neuroimaging

Animals and other organisms

Policy information about studies involving animals; ARRIVE guidelines recommended for reporting animal research

Laboratory animals	RNAseq and whole genome resequencing used lab stocks of Kauai-strain normal-wing males (L) and Hilo-strain flatwing males (G) of Teleogryllus oceanicus. The crickets for whole genome resequencing (L and G) were aged 5-30 days post adult eclosion, and all crickets for RNAseq were penultimate-instar nymphs.
Wild animals	Wings of flatwing male crickets (Teleogryllus oceanicus) were collected from three locations in Hawaii and mounted between two microscope slides (Supplementary Table 1). Single hind legs of male samples (T. oceanicus. and T. commodus) were collected from Australia and Hawaii and preserved in 100% ethanol (Supplementary Table 3) for gDNA extraction.
Field-collected samples	No field collected samples were used in the study.
Ethics oversight	No ethical approval was required.

Note that full information on the approval of the study protocol must also be provided in the manuscript.
