# Supplementary material for: Rapid parallel adaptation despite gene flow in silent crickets
Source: Nat Commun. 2021 Jan 4;12:50. doi: 10.1038/s41467-020-20263-4 (PMC7782688; doi:10.1038/s41467-020-20263-4)
Supplement: Supplementary file 4 — Description of Additional Supplementary Files [file 41467_2020_20263_MOESM4_ESM.pdf]

**Description of Additional Supplementary Files**

Supplementary Data 1. Summary of whole genome re-sequencing data for all 70 crickets.

Supplementary Data 2. Data summary for SNP datasets.

Supplementary Data 3. Summary of *D* statistics (ABBA-BABA test) using LG1 (the X chromosome), corresponding to Fig. 2a.

Supplementary Data 4. Parameters of models estimated in Fastsimcoal2.

Supplementary Data 5 List of all candidate flatwing-associated SNPs, with SNPs passing the Oahu criterion highlighted in yellow.

Supplementary Data 6 Candidate genes associated with flatwing phenotypes.

Supplementary Data 7. Results of tests for flatwing-associated structural variants (SVs) and genes affected by them.

Supplementary Data 8. Results of tests for flatwing-associated copy number variation regions (CNVRs) with annotated protein coding genes.

Supplementary Data 9. Coordinates of SNPs used in Fig. 4b and corresponding $r^2$ values.

Supplementary Data 10. Summary of statistical metrics for Patterson's *D* comparisons between all Hawaiian scenarios and Australian scenarios using LG1 (the X chromosome) and LG2-18 (autosomes), corresponding to Supplementary Fig. 4.
