# Supplementary material for: Rapid parallel adaptation despite gene flow in silent crickets
Source: Nat Commun. 2021 Jan 4;12:50. doi: 10.1038/s41467-020-20263-4 (PMC7782688; doi:10.1038/s41467-020-20263-4)
Supplement: Supplementary file 2 — Peer Review [file 41467_2020_20263_MOESM2_ESM.pdf]

Reviewer #1 (Remarks to the Author):

In this paper the authors use several data sources and analyses to identify the genetic targets of selection in recently evolved "flatwing" phenotypes in crickets in Hawaii. The most notable result is that even though the same genomic region is implicated in 3 populations on different islands, the genetic changes in this region appear to be independent and distinct in each population. This is a remarkable result, particularly given that the authors do also demonstrate ongoing gene flow between the islands.

Definitively proving that the mutations on each island are distinct is very difficult with short read sequence data, since it is extremely difficult to rule out the possibility of a shared causative variant that is missed in the analysis. Nevertheless, the authors do everything reasonably possible to rule this out and overall the combined weight of evidence they present is convincing, particularly, the fact that they never observe clustering of the flat-wing phenotype individuals in phylogenies generated from the associated genomic region.

Overall, I think this is a fascinating piece of work, using an extensive and robust set of data and analyses, that has important implications for the field of evolutionary biology.
I have just a few minor comments that the authors might wish to address before publication.

The authors calculate Patterson's D statistics for the X chromosome but not the autosomes. It might be interesting to compare the D statistics for the X and the autosomes to determine if there is more or less gene flow on the X compared to the autosomes. However, I realise that there would be problems with directly comparing the X and autosomes like this due to differences in effective population size.

The phylogenies for the candidate X regions and associated SNPs are unrooted. This isn't very clear from the way they are presented and rooting would also be interesting in terms of determining the relationships between the populations and phenotypes. It might also be worth highlight the fact that all individuals in these are males and so hemizygous for these markers, meaning that they essentially represent haplotypes.

Figure 4 b is a bit unclear. It is hard to see the lines that I think are meant to link the variants to their position on the scaffold. How big is this region? Is this just LD within the flatwing individuals in each population? I also don't really understand the rationale for using different sets of SNPs in each population. This could mean that the different results for the 2 populations are just due to the different sets of SNPs used.

Was splicing of dsx investigated in the RNAseq data? We would expect to see different male and female isoforms and it would be interesting to know if these differ in their abundance in the flatwing vs normal individuals.

Figure 1 legend, line 127: "lab-raised flatwing Hawaii males" Should this maybe say "Hilo males"?

Figure 2: One of the text boxes in the figure says "Expanse of agricultural land". Should this maybe be "Expansion of agricultural land"?
Figure 2 legend, line 196: "included as controlled comparisons" I think should be "included as control comparisons"

Line 276: I think net nucleotide divergence could be explained more clearly. It is not clear what d(x) and d(y) are.

Line 346: Extra "this"

Figure 5 legend line 398 "(highlighted with box)." Are these the points that are in fact circled in the

figure?
Line 400-401: “female forewings do not noticeably differ[ent] between genotypes”

Line 406: I’m not sure why “parallel” is italicized

Figure S16 was not displayed properly in the uploaded version, some of the panels are overlapping.
Figure S18: It is not clear what this is meant to show. Does the black indicate higher LD (r2) or are there meant to be colours that can’t be seen?

With best wishes,
Nicola Nadeau

Reviewer #2 (Remarks to the Author):

In the presented work, Zhang et al. study three Hawaiian populations of a cricket, Teleogrillus oceanicus, each of which has a “flatwing” morphological variant that is incapable of song. The authors present a fascinating analysis of parallel evolution that is both supported from a number of perspectives such as morphology, qtl mapping, GWAS, and gene expression, and will be of interest to a wider readership with regard to the repeated rapid evolution and the genomic perspective. I think it will be a text book case with these data in hand and strongly influence and inform the field about the genetics of adaptation and should be published. And conceptualization for the figures is brilliant!

I do think the authors should make a more convincing case for gene flow and perhaps reframe the importance of what they have found. I am not convinced that the patterns support gene flow (as opposed to ancestral polymorphism). First, the comparisons involving Australian Teleo with the Hawaii islands should show zero gene flow (D=0). This is supported by geographic distance and the phylogeny in Fig. 1f and presumably why they used Australia as a test case. However, D is not zero but shows a higher allele lineage sharing between Australia and Oahu (or Kauai). The authors don’t confront the possibility that this is due to ancestral polymorphism and for some reason (possibly due to the extent of retained genetic variation on Oahu) ancestral genetic sharing remains highest between Australia and Oahu. The ABBA/BABA test is meant to distinguish between no gene flow (i.e. variation all due to ancestral polymorphism) and asymmetrical gene flow (sharing in excess of ancestral polymorphism) but Australia compared to the Hawaiian populations is meant to be the test case and yet the results are not consistent with that idea (assuming no gene flow from Australia and all allele lineage sharing is due to ancestral polymorphism). So I have my doubts about the test or the demographic assumptions that go into the test.

Turning to the island populations and fueled by the ABBA/BABA results, another interpretation is that there is independent and accentuated divergence in the X region controlling flatwing, relative to ancestral polymorphism levels. And if there was a stepping stone relationship with Oahu giving rise to Kauai, followed by Kauai giving rise to Hilo, the shared ancestral variation might follow the patterns identified (as we saw with Australia and Oahu), but still would not indicate gene flow contemporary with parallel flatwing differentiation. Based on fig. 1f, it would appear that Oahu was the initial Hawaiian population, and then gave rise to Kauai and perhaps Kauai giving rise to Hilo. This migration pattern might have occurred before the independent origins of flatwing. The authors do not appear to have considered this possibility. I think the work is brilliant despite this point and some additional discussion on it is both possible, and would not undermine the fascinating story of parallel evolution and the evidence they provide.

One small point: regarding the estimation of phylogenetic trees, the methods section states that neighbor joining was used, whereas in the figures, it states a maximum likelihood tree is presented. ML details were not described. And in addition, 100 bootstrap replicates is probably too small. It would be better to set that the replicates at 1000 although I don’t know what computational constraints

there were. I would advise that the authors comb through all the methods used to ensure accuracy of the presentation.

Reviewer #3 (Remarks to the Author):

General comments:

Zhang et al., aim to compare candidate regions associated with phenotypic shifts in wing morphology across replicate populations of crickets. The authors ask if the same genomic regions contribute to the observed phenotype and whether these regions have a shared (due to gene flow) or independent (due to de novo mutation) origin. The study provides novel insight on the genetic and ecological mechanisms shaping patterns of repeated phenotypic evolution. I believe this study would be of interest to many researchers interested in the predictability of evolutionary trajectories. The authors have conducted a robust study integrating different datasets and combining multiple appropriate analytical methods. Correspondingly, my comments focus largely on the conceptual framework, which I found fairly difficult to follow a times.

Throughout the manuscript I find the use of the terms "convergent" and "parallel" to be distracting and confusing. The use of this jargon (which is also contentiously defined) unnecessarily complicates the conversation. It seems to me that the point of this paper is to 1) determine is there repeated use of the same genes and/or mutations during adaptive wing loss? And 2) ask if any observed 're-use' is due to independent mutations or adaptive gene flow? I would recommend simplifying the framework of the paper. As written, there were several paragraphs where I spent an unreasonable amount of time puzzling through the logic of the argument (some noted with line numbers below).

I would argue that some people would consider the repeated fixation of an adaptive allele introduced at low frequency by gene flow to be an example of "parallel" evolution, as this mechanism is hardly different from parallel evolution due to standing genetic variation. I don't think the argument that "parallelism is constrained by gene flow" is widely known. It would be worthwhile developing and bolstering this concept.

In the results section the interpretation of each result is often very obscured by unnecessary text or jargon. Say explicitly if an observed pattern supports the hypothesis of parallel evolution or convergent evolution (as defined by you). Alternatively, clearly state whether the region is 'shared' or not, or if the pattern is due to gene flow or not. Simplify your observations/conclusions so the reader doesn't have to dig. For example, I am still somewhat uncertain about the final result described in the section about structural variants. The results are finally more clearly stated in the Discussion.

Lines 427 -439 Are the observed migration rates for your crickets 'high' or 'low' relative to other estimates (From other species/systems)? Perhaps the effective gene flow isn't sufficient to affect selection on novel variants - this merits discussion.

Additional specifics:

Lines 17 – 20. I had to re-read this sentence multiple times to figure out what the message was. As written, it is confusing because the effect of gene flow is introduced after an adaptation is stated as already having occurred. I would flip the order of the introduced concepts. It would also be useful to clearly define your usage of "convergent evolution" – you don't mention mutation in this definition like you do for "parallel evolution". How do you define convergent at the genotype level? Also what level are you considering for parallelism – mutations in the same gene or the same mutation?

Lines 126-133 very difficult to follow. Could more clearly state that there is evidence of a shared molecular basis in Kauai and Hilo but that Oahu appears distinct.

Lines 230-233 difficult to interpret. Do you mean that you found associations in each population, but they were not shared across island or that there were no associations in any of the island populations? Are you only talking about associations with the structural variants? How are no shared associations "consistent with the hypothesis of parallel evolution"?

Line 419 What are the "multiple signals of parallel evolution"? These would be worth stating explicitly.

REVIEWER COMMENTS

Reviewer #1

In this paper the authors use several data sources and analyses to identify the genetic targets of selection in recently evolved "flatwing" phenotypes in crickets in Hawaii. The most notable result is that even though the same genomic region is implicated in 3 populations on different islands, the genetic changes in this region appear to be independent and distinct in each population. This is a remarkable result, particularly given that the authors do also demonstrate ongoing gene flow between the islands.

Definitively proving that the mutations on each island are distinct is very difficult with short read sequence data, since it is extremely difficult to rule out the possibility of a shared causative variant that is missed in the analysis. Nevertheless, the authors do everything reasonably possible to rule this out and overall the combined weight of evidence they present is convincing, particularly, the fact that they never observe clustering of the flat-wing phenotype individuals in phylogenies generated from the associated genomic region.

Overall, I think this is a fascinating piece of work, using an extensive and robust set of data and analyses, that has important implications for the field of evolutionary biology.

We are grateful for these positive comments.

I have just a few minor comments that the authors might wish to address before publication.

The authors calculate Patterson's D statistics for the X chromosome but not the autosomes. It might be interesting to compare the D statistics for the X and the autosomes to determine if there is more or less gene flow on the X compared to the autosomes. However, I realise that there would be problems with directly comparing the X and autosomes like this due to differences in effective population size.

In our revised manuscript, we calculated Patterson's D for the autosomes (other linkage groups) as suggested by the reviewer (Supplementary Fig. 4). Although we agree it would be problematic to directly compare the X and autosomes, we note that the X chromosome (LG1) showed the strongest signals of gene flow, and almost all autosomal LGs showed qualitative patterns consistent with our original result (lines 155-158):

"Although it would be problematic to directly compare D values of X chromosome and autosomes, we nevertheless note that the X (LG1) showed the strongest signals of gene flow, and the large majority of autosomal linkage groups (LGs) showed qualitative patterns consistent with the X (Supplementary Fig. 4)."

The phylogenies for the candidate X regions and associated SNPs are unrooted. This isn't very clear from the way they are presented and rooting would also be interesting in terms of

determining the relationships between the populations and phenotypes. It might also be worth highlight the fact that all individuals in these are males and so hemizygous for these markers, meaning that they essentially represent haplotypes.

We now include rooted and unrooted trees, all with 1000 bootstrap replicates (described at lines 221-224). Rooted trees yielded qualitatively identical results (new Fig. 3d, Supplementary Figs. 17-25). As suggested, we now mention that these X-derived trees suggest relationships of different haplotypes due to the hemizygous state of the X chromosome (lines 225-227).

Figure 4 b is a bit unclear. It is hard to see the lines that I think are meant to link the variants to their position on the scaffold. How big is this region? Is this just LD within the flatwing individuals in each population? I also don't really understand the rationale for using different sets of SNPs in each population. This could mean that the different results for the 2 populations are just due to the different sets of SNPs used.

We have re-performed these analyses and revised Figure 4b to address this point.

First we summarise why it is not possible to use the same sets of SNPs for each population (these points are clarified in the Methods section at lines 525-530): The panel represents the whole scaffold 18404, spanning approximately 1.3 Mb. To illustrate LD patterns in the flanking regions of flatwing-associated SNPs, only wild flatwing individuals in each population were used. It was impossible to use the exact same sets of SNPs in both groups, because 1) different sets of positions were polymorphic in flatwing Oahu and flatwing Kauai individuals, 2) LD cannot be calculated between polymorphic SNPs and non-polymorphic sites, 3) to show the general pattern of LD in this genomic region, a reasonable number of SNPs dispersed across the whole scaffold is necessary.

We agree with the reviewer that using substantially different sets of SNPs from each population could make this LD comparison on scaffold 18404 more difficult to interpret. We took several steps to overcome this issue. First, we now include a new supplementary figure (Supplementary Fig. 28 in the revised manuscript) to show scaffold 18404-wide patterns of decay of LD for Kauai and Oahu flatwing groups. This new analysis includes all SNPs in this scaffold, and quantitatively compares LD calculated using different SNP sets for each island, showing clearly the different patterns of decay over physical distance in Kauai (long-range LD) and Oahu (lower scaffold-wide LD).

Second, we developed a method to replot Figure 4b to minimize visual bias caused by using different SNP sets from either population: We used the flatwing Oahu group as a standard and then selected the corresponding SNP in the Kauai group; for sites at which the same SNP was unavailable in Kauai, the nearest one in its flanking regions was selected. The resulting plot (new Fig. 4b) provides a more directly comparable visual depiction of LD in both populations. We have added these details in the Methods section (lines 528-530) and provide a supplementary table to report the coordinates of the SNPs used in Fig. 4b and the corresponding r^2 values (Supplementary Table 18). All of these results support our original interpretation for evidence of long-range complete LD in Kauai and a much more modest elevation of LD in Oahu.

Was splicing of *dsx* investigated in the RNAseq data? We would expect to see different male and female isoforms and it would be interesting to know if these differ in their abundance in the flatwing vs normal individuals.

We did detect *dsx* splice variants, but found no evidence of sex- or morph- segregating isoforms in our RNAseq data. We are hesitant to speculate further, though we now note with interest in our manuscript that there appear to be more exceptions to sex-specific splicing in arthropods than may have been previously appreciated[1] (lines 315-318).

"We found no evidence of sex- or morph-segregating dsx *isoforms. We cannot exclude that these might be detected at a different developmental stage or in different tissues, but note with interest that there are now multiple exceptions to the expectation of sex-specific splicing in arthropods [1]. "*

Reference:

1 Price, D. C., Egizi, A. & Fonseca, D. M. The ubiquity and ancestry of insect *doublesex*. *Sci Rep* 5, 13068 (2015).

Figure 1 legend, line 127: "lab-raised flatwing Hawaii males" Should this maybe say "Hilo males"?

Thank you for catching this; corrected (line 930).

Figure 2: One of the text boxes in the figure says "Expanse of agricultural land". Should this maybe be "Expansion of agricultural land"?

Changed as suggested (Fig. 2b).

Figure 2 legend, line 196: "included as controlled comparisons" I think should be "included as control comparisons"

Changed as suggested (line 939).

Line 276: I think net nucleotide divergence could be explained more clearly. It is not clear what d(x) and d(y) are.

We now define terms in the methods section at lines 605-609, flag this in the main text (Line 236), and clarify that:

"Absolute divergence (d_{xy}) and net nucleotide divergence (d) were used to estimate the amount of DNA divergence (nucleotide substitutions). For the latter, $d = d_{xy}-(d_x+d_y)/2$, where d_x and d_y are per-site pairwise nucleotide differences within populations X and Y, respectively, and d_{xy} represents pairwise nucleotide differences between populations X and Y. Estimates of d and…"

Line 346: Extra "this"

Fixed (line 282).

Figure 5 legend line 398 "(highlighted with box)." Are these the points that are in fact circled in the figure?

Yes, thanks for catching this. Corrected (line 1003).

Line 400-401: "female forewings do not noticeably differ[ent] between genotypes"

Corrected (line 1006).

Line 406: I'm not sure why "parallel" is italicized

This was for emphasis, but we agree it is not needed. Fixed (line 333).

Figure S16 was not displayed properly in the uploaded version, some of the panels are overlapping.

Hopefully this is corrected in the resubmission (Supplementary Fig. 26).

Figure S18: It is not clear what this is meant to show. Does the black indicate higher LD (r^2) or are there meant to be colours that can't be seen?

Yes, darker shades represent higher LD. We have regenerated this figure and added a scale bar to make this clearer. (Supplementary Fig. 29)

Reviewer #2

In the presented work, Zhang *et al*. study three Hawaiian populations of a cricket, *Teleogrillus oceanicus*, each of which has a "flatwing" morphological variant that is incapable of song. The authors present a fascinating analysis of parallel evolution that is both supported from a number of perspectives such as morphology, qtl mapping, GWAS, and gene expression, and will be of interest to a wider readership with regard to the repeated rapid evolution and the genomic perspective. I think it will be a text book case with these data in hand and strongly influence and inform the field about the genetics of adaptation and should be published. And conceptualization for the figures is brilliant!

Thank you for your positive assessment and enthusiasm for our study.

I do think the authors should make a more convincing case for gene flow and perhaps reframe the importance of what they have found. I am not convinced that the patterns support gene flow (as opposed to ancestral polymorphism). First, the comparisons involving Australian Teleo with

the Hawaii islands should show zero gene flow (D=0). This is supported by geographic distance and the phylogeny in Fig. 1f and presumably why they used Australia as a test case. However, D is not zero but shows a higher allele lineage sharing between Australia and Oahu (or Kauai). The authors don't confront the possibility that this is due to ancestral polymorphism and for some reason (possibly due to the extent of retained genetic variation on Oahu) ancestral genetic sharing remains highest between Australia and Oahu. The ABBA/BABA test is meant to distinguish between no gene flow (i.e. variation all due to ancestral polymorphism) and asymmetrical gene flow (sharing in excess of ancestral polymorphism) but Australia compared to the Hawaiian populations is meant to be the test case and yet the results are not consistent with that idea (assuming no gene flow from Australia and all allele lineage sharing is due to ancestral polymorphism). So I have my doubts about the test or the demographic assumptions that go into the test.

We thank the reviewer for raising this issue. We think a potential source of confusion is that all of our D comparisons involve populations of the same species, and we now emphasise this point in our explanation at lines 145-151. It is correct that the ABBA/BABA test is meant to test whether P3 exchanged genes with P1 or P2 after these two populations diverged. However, our result that D values involving any of the three Australian scenarios (orange bars of Fig. 2a) deviate from the null hypothesis (D = 0) is not surprising, because each involves different populations of the same species (not multiple different species). Modest but significant gene flow shared among different populations within one species should be common, even though considerable geographic distances may exist and distinct phylogenetic relationships are detectable. For example, similar patterns of gene flow have been repeatedly reported in geographically isolated insects (e.g. *Helicoverpa armigera*)[1], plants (e.g. *Cupressus chengiana*)[2], and mammals (e.g. *Bos grunniens*)[3]; we have re-written lines 148-151 to explain this.

<u>**References:**</u>

1 Anderson, C. J., Tay, W. T., McGaughran, A., Gordon, K. & Walsh, T. K. Population structure and gene flow in the global pest, *Helicoverpa armigera*. *Mol Ecol* 25, 5296-5311 (2016).

2 Li, J. et al. Allopatric divergence and hybridization within *Cupressus chengiana* (Cupressaceae), a threatened conifer in the northern Hengduan Mountains of western China. *Mol Ecol* 29, 1250-1266 (2020).

3 Qiu, Q. et al. Yak whole-genome resequencing reveals domestication signatures and prehistoric population expansions. *Nat Commun* 6, 10283 (2015).

Our purpose for using three comparisons involving the Australian population is to show that gene flow shared among the three Hawaii island populations was considerably and significantly stronger than gene flow between Hawaii and Australia. As Reviewer 3 points out (see also our responses below), it can be difficult to give context to how much gene flow is "a lot", and we feel that this intraspecific comparison helps put the high levels of gene flow within Hawaii into a wider context. We explain this further at lines 151-154. In this analysis, our interpretation of high gene flow was supported by more extreme D values (significantly larger |D|) of the three Hawaii island scenarios (right three bars of Fig. 2a) compared to the control Australian scenarios (all $P < 0.01$). In addition

to the additional text mentioned above, we have also added a supplementary table (Supplementary Table 7) to reported the details of the statistic tests which were used to compare D values.

"We used two approaches to evaluate gene flow. First, we implemented Patterson's D statistics (ABBA-BABA test) using X chromosome data because we were specifically interested in understanding gene flow that could affect the movement of flatwing alleles among populations. This analysis revealed considerable gene flow among the three Hawaiian island populations (right three bars of Fig. 2a), which was considerably and significantly higher than that involving Australian populations (orange bars of Fig. 2a, Supplementary Tables 6,7). All comparisons yielded D values which deviated significantly from the null hypothesis (D = 0), which is consistent with common observations that modest but significant gene flow can be detected among geographically distinct distant populations within the same species[1-3]. The key observation is that all Hawaii island scenarios showed significantly more extreme D values compared to gene flow involving Australian populations (all P < 0.01, Supplementary Table 7), providing unambiguous evidence for strong gene flow among the three island populations …"

Turning to the island populations and fueled by the ABBA/BABA results, another interpretation is that there is independent and accentuated divergence in the X region controlling flatwing, relative to ancestral polymorphism levels. And if there was a stepping stone relationship with Oahu giving rise to Kauai, followed by Kauai giving rise to Hilo, the shared ancestral variation might follow the patterns identified (as we saw with Australia and Oahu), but still would not indicate gene flow contemporary with parallel flatwing differentiation. Based on fig. 1f, it would appear that Oahu was the initial Hawaiian population, and then gave rise to Kauai and perhaps Kauai giving rise to Hilo. This migration pattern might have occurred before the independent origins of flatwing. The authors do not appear to have considered this possibility.

An important point which we emphasise at lines 40-50 is that the flatwing morphotype first appeared in the Kauai population very recently – ca. 2003 [1,2]. In 2005, the silent morph appeared in a population on the island of Oahu, and then in Hilo in 2010 [1,2,3]. Flatwing crickets have never been detected outside of Hawaii (now noted at lines 50-51), which argues strongly against an ancestral origin of this phenotype outside of the Hawaiian islands. Flatwing is a recent adaptation in these populations (within 10~20 years) which all available evidence suggests occurred after the crickets colonized all three Hawaiian islands more than 400 years ago.

References

1 Zuk, M., Rotenberry, J. T. & Tinghitella, R. M. Silent night: adaptive disappearance of a sexual signal in a parasitized population of field crickets. *Biol Lett* 2, 521-524 (2006).

2 Zuk, M., Bailey, N. W., Gray, B. & Rotenberry, J. T. Sexual signal loss: The link between behaviour and rapid evolutionary dynamics in a field cricket. *J Anim Ecol* 87, 623-633 (2018).

3 Pascoal, S. *et al*. Rapid convergent evolution in wild crickets. *Curr Biol* 24, 1369-1374 (2014).

Nevertheless, we agree with the reviewer that the ABBA-BABA test alone might not be convincing enough, because it is possible for the results of that test to reflect ancient gene flow among islands instead of recent, ongoing gene flow. That is why we additionally used Fastsimcoal2 to test 62

models of recent demographic history, which we described in detail starting at line 159. These models can be classified into 2 major types of scenario describing whether or not recent ongoing gene flow exists among the three islands (Supplementary Fig. 5, Supplementary Table 9, Fig. 2b). The gene flow which we report in the second panel of Figure 2 from Fastsimcoal2 analyses was inferred to have started recently, and occurred continuously until the present, rather than transiently, which we now emphasise at lines 168-169. Almost all scenarios assuming recent ongoing gene flow were more probable than those without gene flow (Supplementary Table 9), and this fits with known patterns of human colonisation and inter-island transport in Hawaii. The best-fit models of these 62 models (Fig 2b) supported a stepping stone relationship among these three islands, and more importantly, a pattern of symmetrical gene flow starting within the last 300~400 years and continuing today. The numbers underneath the blue arrows of Fig. 2b are migration rates (per generation) which we now better emphasise in the figure legend (lines 951-952). We believe this result combined with the ABBA-BABA results provide convincing support that recent, ongoing gene flow has been occurring after the crickets colonized all three Hawaiian islands more than 400 years ago, as opposed to a scenario of incomplete sorting of ancestral polymorphism.

Finally, while it is theoretically possible that the continuous gene flow indicated by our Fastsimcoal2 analysis started 300~400 years ago but was interrupted in the most recent 10~15 years, this is a less parsimonious explanation. We now discuss this in a new paragraph at lines 345-351. *T. oceanicus* populations on the Hawaiian islands have, like many island species, lost long-distance flight capabilities, and migration (gene flow) appears to be maintained by human activities instead. In Hawaii, inter-island human activities became more frequent as human settlement increased 300 years ago, so if anything recent gene flow should be even stronger than before.

I think the work is brilliant despite this point and some additional discussion on it is both possible, and would not undermine the fascinating story of parallel evolution and the evidence they provide.

Thank you for the supportive comments. We have added a new Discussion paragraph to acknowledge that a pattern of gene flow interrupted in the last ~15 years cannot be completely excluded, though for the reasons stated above we assess this as unlikely (lines 345-351).

"It is theoretically possible that the continuous gene flow indicated by our Fastsimcoal2 analysis started 300~400 years ago only to be interrupted in the last 10 - 15 years, but this is a less parsimonious explanation. T. oceanicus populations on the Hawaiian islands have, like many island species, lost long-distance flight capability, and migration appears to be maintained by human activities instead. In Hawaii, inter-island human activities became more frequent as human settlement increased 300 years ago, so if anything recent gene flow should be even stronger than before."

One small point: regarding the estimation of phylogenetic trees, the methods section states that neighbor joining was used, whereas in the figures, it states a maximum likelihood tree is presented. ML details were not described. And in addition, 100 bootstrap replicates is probably

too small. It would be better to set that the replicates at 1000 although I don't know what computational constraints there were. I would advise that the authors comb through all the methods used to ensure accuracy of the presentation.

Both the NJ method and ML method were used. The autosomal tree (Fig. 1f) and X chromosomal tree (Supplementary Fig. 3) were established using the NJ method to economize computing resources. The ML method was used to establish phylogenetic trees for the candidate X regions and associated SNPs (Fig. 3c,d, Supplementary Figs. 8-25). In this revised manuscript, we have added more detail to describe these methods clearly (lines 513, 604-605). We have also re-established all phylogenetic trees (NJ trees and ML trees) with 1000 bootstrap replicates (Fig 1f, Fig. 3c,d, Supplementary Figs. 2-3, 8-25). All of these trees yielded qualitatively consistent results. We have also combed through all the methods used to ensure the accuracy of our presentation.

Reviewer #3

General comments:

Zhang et al., aim to compare candidate regions associated with phenotypic shifts in wing morphology across replicate populations of crickets. The authors ask if the same genomic regions contribute to the observed phenotype and whether these regions have a shared (due to gene flow) or independent (due to de novo mutation) origin. The study provides novel insight on the genetic and ecological mechanisms shaping patterns of repeated phenotypic evolution. I believe this study would be of interest to many researchers interested in the predictability of evolutionary trajectories. The authors have conducted a robust study integrating different datasets and combining multiple appropriate analytical methods. Correspondingly, my comments focus largely on the conceptual framework, which I found fairly difficult to follow a times.

We are grateful for the positive assessment and thoughtful response. We have revised the manuscript to tighten the conceptual framework. Our responses to R1 and R2 address some of these issues, and we enumerate the remaining changes below.

Throughout the manuscript I find the use of the terms "convergent" and "parallel" to be distracting and confusing. The use of this jargon (which is also contentiously defined) unnecessarily complicates the conversation. It seems to me that the point of this paper is to 1) determine is there repeated use of the same genes and/or mutations during adaptive wing loss? And 2) ask if any observed 're-use' is due to independent mutations or adaptive gene flow? I would recommend simplifying the framework of the paper. As written, there were several paragraphs where I spent an unreasonable amount of time puzzling through the logic of the argument (some noted with line numbers below).

I would argue that some people would consider the repeated fixation of an adaptive allele

introduced at low frequency by gene flow to be an example of "parallel" evolution, as this mechanism is hardly different from parallel evolution due to standing genetic variation. I don't think the argument that "parallelism is constrained by gene flow" is widely known. It would be worthwhile developing and bolstering this concept.

We agree that there are contentious semantic issues in the field of convergent evolution. It is not our intention to re-litigate these definitional issues in our study, so we have made revisions to shift focus away from terminology, and instead spell out the underlying processes that we studied. Wherever possible, we removed or replaced unnecessarily complex or opaque terminology (e.g. lines 33, 37, 333). We cannot remove all such terms from our study, but we believe the balance is now appropriate.

Regarding the conceptual basis of the study; we consider that the difficulties Reviewer 3 identified might arise from different conventions of language around convergence and parallelism in different subfields – e.g. palaeontology vs. molecular biology. To ensure that readers from diverse disciplines have a unified understanding of our work, we have made a number of revisions to ensure precision of language and a clear conceptual framework. We defined concepts more explicitly at the start of the manuscript, especially making two critical concepts more distinct. The first is to delineate that we are studying convergent evolution at the level of organismal phenotypes (i.e. loss of male song). We emphasise this by defining "convergent phenotypic evolution" (e.g. lines 15-24). The second key clarification is that we more cautiously use the words "allele" vs. "genetic variant", to avoid causing confusion by inadvertently conflating the two. We think this pair of changes, plus our re-writing of several sections and inclusion of additional theory references described below, makes the conceptual basis of the study unambiguous. E.g. at the beginning (lines 15-24):

"Adaptive evolution can be surprisingly repeatable. Convergent phenotypic evolution occurs when similar selection pressures drive the spread of functionally identical traits at the organismal level, but in different populations[1,2]. One way this can occur is through adaptive introgression, by repeatedly introducing the same pre-existing adaptive allele from one population into others, whereupon they are selected. In this scenario, gene flow is predicted to play an important role in moving the same allelic variants, which share a single mutational origin, among populations[3]. In contrast, parallel evolution occurs when traits that are functionally identical at the organismal level nonetheless have independent mutational origins at the genetic level[3,4]. In the latter case, uniquely-arisen adaptive genetic variants are independently selected in different populations, and theory strongly predicts that high levels of gene flow will reduce the likelihood of parallel evolution[5-10]."

Additional theory references

5 Ralph, P. L. & Coop, G. Convergent Evolution During Local Adaptation to Patchy Landscapes. *Plos Genet* 11, e1005630 (2015).

6 Ralph, P. & Coop, G. Parallel adaptation: one or many waves of advance of an advantageous allele? *Genetics* 186, 647-668 (2010).

7 Feder, A. F., Pennings, P. S., Hermisson, J. & Petrov, D. A. Evolutionary Dynamics in Structured Populations Under Strong Population Genetic Forces. *G3 (Bethesda)* 9, 3395-3407 (2019).

8 Patwa, Z. & Wahl, L. M. The fixation probability of beneficial mutations. *J R Soc Interface* 5,

1279-1289 (2008).
9 Aeschbacher, S. & Burger, R. The effect of linkage on establishment and survival of locally beneficial mutations. *Genetics* 197, 317-336 (2014).
10 Bradburd, G. S. & Ralph, P. L. Spatial Population Genetics: It's About Time. *Annual Review of Ecology, Evolution, and Systematics*, Vol 50 50, 427-449 (2019).

We believe these revisions avoid the risk of over-complicating the (contentious) distinctions between convergence and parallelism. They also help clarify the point of the study (which we note is not about wing loss in crickets. It is about the loss of male-specific wing venation, which causes the loss of their sexual advertisement song as described at lines 39-44 and in Fig. 1a). The point of the study is first to establish whether the same, or different, genetic variant underlie(s) the same adaptive loss of song in different Hawaiian island populations of crickets. Gene flow is critical to these scenarios, because it is expected to be an important mover of adaptive alleles from one population to another. Thus, when evolution of the same trait is observed to be recurrent, rapid, and recent, it would be predicted to have arisen due to gene flow rather than independent mutational events in separate populations. We think the reviewer is correct that this is not a widely-appreciated argument, but to us that makes it all the more important to articulate, as we have done at lines 22-24 with additional citations to examples and theoretical arguments from the growing literature on convergent/parallel evolution.

In the results section the interpretation of each result is often very obscured by unnecessary text or jargon. Say explicitly if an observed pattern supports the hypothesis of parallel evolution or convergent evolution (as defined by you). Alternatively, clearly state whether the region is 'shared' or not, or if the pattern is due to gene flow or not. Simplify your observations/conclusions so the reader doesn't have to dig. For example, I am still somewhat uncertain about the final result described in the section about structural variants. The results are finally more clearly stated in the Discussion.

As mentioned above, we made changes throughout the manuscript to tighten the language and make it straightforward for readers to interpret our findings (e.g. lines 168-169, 236-237, 242-247, 352-360). We agree with the reviewer that the structural variants section was confusing as originally written. To streamline it, we edited and moved the text about structural variants to the end of the section (see below).

Lines 427 -439 Are the observed migration rates for your crickets 'high' or 'low' relative to other estimates (From other species/systems)? Perhaps the effective gene flow isn't sufficient to affect selection on novel variants - this merits discussion.

The reviewer raises an important point. Some of our revisions in response to R2 address this by contextualising the levels of inter-island gene flow using Paterson's D (lines 145-154). We also now provide additional references to published literature [1-3] to highlight that gene flow among these Hawaiian island populations is comparatively high. However, and although we view it as unlikely, the possibility that gene flow among these populations was insufficient to affect selection on novel variants cannot be definitively excluded, so we added a discussion paragraph as suggested (lines

367-379).

"We cannot definitively exclude the possibility that the observed level of gene flow in our study might have been insufficient to affect selection on novel variants, however contrasting our results with empirical estimates of gene flow from other studies suggests this is unlikely to be the case[1-3]. Although it can be difficult to compare migration rates across studies due to different sampling strategies, methods used for estimating migration, and different underlying demographic models, we note that the gene flow we detected among three Hawaiian island populations of T. oceanicus *was particularly strong compared to estimates in other species[1-3]. The precise dynamics of mutation, migration and drift in shaping adaptation is a longstanding and complex problem, so in the silent flatwing cricket system and in other systems undergoing rapid convergent evolution, it would be illuminating to perform evolve and resequence experiments to define threshold levels of gene flow beyond which parallel mutations localised within distinct populations become swamped by adaptive variants originating from other populations."*

References

1 Corl, A. *et al.* The Genetic Basis of Adaptation following Plastic Changes in Coloration in a Novel Environment. *Curr Biol* 28, 2970-2977 e2977 (2018).

2 Sprengelmeyer, Q. D. *et al.* Recurrent Collection of Drosophila melanogaster from Wild African Environments and Genomic Insights into Species History. *Mol Biol Evol* 37, 627-638 (2020).

3 Lujan, N. K., Weir, J. T., Noonan, B. P., Lovejoy, N. R. & Mandrak, N. E. Is Niagara Falls a barrier to gene flow in riverine fishes? A test using genome-wide SNP data from seven native species. *Mol Ecol* 29, 1235-1249 (2020).

Additional specifics:

Lines 17 – 20. I had to re-read this sentence multiple times to figure out what the message was. As written, it is confusing because the effect of gene flow is introduced after an adaptation is stated as already having occurred. I would flip the order of the introduced concepts. It would also be useful to clearly define your usage of "convergent evolution" – you don't mention mutation in this definition like you do for "parallel evolution". How do you define convergent at the genotype level? Also what level are you considering for parallelism – mutations in the same gene or the same mutation?

Please see remarks above. We now provide a clearer definition of convergent evolution at lines 15-24, 384-385. Generally, we use the term "convergent" at the phenotypic level (i.e. functionally identical traits), and use the terms "parallel" and "introgression" only at the genetic level.

Lines 126-133 very difficult to follow. Could more clearly state that there is evidence of a shared molecular basis in Kauai and Hilo but that Oahu appears distinct.

We can see how this section was confusing as written and have now modified it (lines 126-128).

Lines 230-233 difficult to interpret. Do you mean that you found associations in each population,

but they were not shared across island or that there were no associations in any of the island populations? Are you only talking about associations with the structural variants? How are no shared associations "consistent with the hypothesis of parallel evolution"?

The text the reviewer referred to was confusing as originally written. To streamline it, we have move discussion of structural variants to the end of the section (lines 239-247), so that it no longer interrupts the description of SNP characterisation and GWAS, and we clarify the questions the reviewer asked:

"Structural variants (SVs) can have a strong influence on adaptive evolution in insects. We identified a total of 6,971 deletions, 4,482 translocations, 3,385 inversions, 211 insertions and 17,670 copy number variation regions (CNVRs) located in the X chromosome among Kauai, Oahu and Hilo populations (Supplementary Fig. 27, Supplementary Table 14). If the same structural variants were associated with flatwing morphology on different islands, this would be inconsistent with independent mutational origins of flatwing in those populations. However, we did not uncover any candidate association between structural variants and the flatwing phenotype within islands except for Kauai. (Supplementary Fig. 27 and Supplementary Tables 15-16), consistent with the hypothesis of parallel evolution."

Line 419 What are the "multiple signals of parallel evolution"? These would be worth stating explicitly.

We now state these explicitly as suggested (lines 352-360).

"Despite evidence for considerable gene flow, our morphometric, genomic and transcriptomic analyses showed multiple signals of independent origins of the silent flatwing male phenotype in different island populations. These were: 1) distinct morphometric patterns of flatwing venation on different islands, 2) entirely non-overlapping flatwing-associated SNPs among all three populations, 3) all phylogenetic trees for flatwing-associated genetic regions falsified a scenario of adaptive introgression 4) greater net nucleotide divergence at flatwing-associated regions 5) lack of shared flatwing-associated structural variants 6) long-range, complete linkage disequilibrium on flatwing-associated scaffolds in Kauai but not Oahu 7) little overall similarity in wingbud gene expression changes associated with flatwing genotypes from different islands."

\Reviewer #1 (Remarks to the Author):

The authors have addressed all of my previous concerns.

However, the revisions have introduced a couple of points that I think should be amended.

Firstly the authors now assert, on line 153, that the D value results are "providing unambiguous evidence for strong gene flow among the three island populations." I think this is incorrect as these results could be due to incomplete lineage sorting during the initial colonisation. Therefore, I think this statement should be removed.

Secondly (in response to one of previous comments) the authors have added "Although it would be problematic to directly compare D values of the X chromosome and autosomes..." I think there should be an explanation of why is it problematic (i.e. because the effective population sizes differ).

I also think that much of the additional discussion about migration between the islands, which has been added in the revision, is unnecessary and distracting. Much of this seems to have been introduced in response to the previous Reviewer 2's comments that they thought the case for migration was not convincing. I agree to some extent, in particular that the ABBA-BABA results on their own are not very convincing, but I think that overall (with the Fastcoal results) the evidence is convincing enough for the conclusions to hold. On the other hand I do agree that these results are not the most interesting or strongest part of the manuscript, and I think the additional emphasis that is now placed on them in the discussion detracts from the more interesting points.

Reviewer #2 (Remarks to the Author):

Zhang et al. study three Hawaiian populations of a cricket, Teleogryllus oceanicus, each of which has a “flatwing” morphological variant that is incapable of song. The authors present an analysis of parallel evolution that is supported by a number of approache. This study will be of interest to a wider readership with regard to the repeated rapid evolution and the genomic perspective.

This is a second review and after thoroughly reading all the reviews and author's responses and edits, I am satisfied that the authors addressed the reviewers concerns, particularly my own with regard to gene flow and ABBA/BABA tests. ABBA/BABA tests are in wide use at this point in time, and have an intuitive appeal. Importantly, as the authors note, they use this method in combination with a demographic modeling approach to bolster their conclusions about gene flow. The authors present the results with appropriate levels of caution. This layer of analysis offers an interesting context for understanding the "re-use" genetic origins story of flatwing.

Reviewer #3 (Remarks to the Author):

This manuscript is a resubmission of a paper I reviewed previously, I am very pleased by the thorough and adequate responses that the authors have given to the critiques put forth by all three prior reviewers (including myself). Upon reading the revised manuscript my initial reservations are satisfied and I do not have any additional revisions to suggest. Again, I think this is a fairly comprehensive study and a neat example of the repeatability of evolutionary trajectories. I think this paper will be of interest to researchers in a variety of evolutionary sub fields.

REVIEWER COMMENTS

Reviewer #1:

The authors have addressed all of my previous concerns.
We are grateful for the reviewers' continued interest in our manuscript.

However, the revisions have introduced a couple of points that I think should be amended.

Firstly the authors now assert, on line 153, that the D value results are "providing unambiguous evidence for strong gene flow among the three island populations." I think this is incorrect as these results could be due to incomplete lineage sorting during the initial colonisation. Therefore, I think this statement should be removed.

We removed this statement as suggested. (line 164)

Secondly (in response to one of previous comments) the authors have added "Although it would be problematic to directly compare D values of the X chromosome and autosomes..." I think there should be an explanation of why is it problematic (i.e. because the effective population sizes differ).

Changed as suggested (line 167).

I also think that much of the additional discussion about migration between the islands, which has been added in the revision, is unnecessary and distracting. Much of this seems to have been introduced in response to the previous Reviewer 2's comments that they thought the case for migration was not convincing. I agree to some extent, in particular that the ABBA-BABA results on their own are not very convincing, but I think that overall (with the Fastcoal results) the evidence is convincing enough for the conclusions to hold. On the other hand I do agree that these results are not the most interesting or strongest part of the manuscript, and I think the additional emphasis that is now placed on them in the discussion detracts from the more interesting points.

We have streamlined these sections of the Discussion to remove redundant text and ensure that the discussion does not stray from our key findings, while retaining information about migration and the strength of gene flow useful for appropriately interpreting our results (lines 372-373, 381-385, 393-395).

Reviewer #2 (Remarks to the Author):

Zhang et al. study three Hawaiian populations of a cricket, Teleogryllus oceanicus, each of which has a "flatwing" morphological variant that is incapable of song. The authors present an analysis of parallel evolution that is supported by a number of approache. This study will be of interest to a wider readership with regard to the repeated rapid evolution and the genomic perspective.

This is a second review and after thoroughly reading all the reviews and author's responses and edits, I am satisfied that the authors addressed the reviewers concerns, particularly my own with regard to gene flow and ABBA/BABA tests. ABBA/BABA tests are in wide use at this point in time, and have an intuitive appeal. Importantly, as the authors note, they use this method in combination with a demographic modeling approach to bolster their conclusions about gene flow. The authors present the results with appropriate levels of caution. This layer of analysis offers an interesting context for understanding the "re-use" genetic origins story of flatwing.

We are grateful for the positive assessment.

Reviewer #3 (Remarks to the Author):

This manuscript is a resubmission of a paper I reviewed previously, I am very pleased by the thorough and adequate responses that the authors have given to the critiques put forth by all three prior reviewers (including myself). Upon reading the revised manuscript my initial reservations are satisfied and I do not have any additional revisions to suggest. Again, I think this is a fairly comprehensive study and a neat example of the repeatability of evolutionary trajectories. I think this paper will be of interest to researchers in a variety of evolutionary sub fields.

Again, we thank the reviewers for their interest in our work.
